# Atomically Dispersed Sn on Core‐Shell MoS_2_ Nanoreactors as Mott‐Schottky Phase Junctions for Efficient Electrocatalytic Hydrogen Evolution

**DOI:** 10.1002/adma.202502977

**Published:** 2025-05-06

**Authors:** Hao Jin, Yan Zhang, Zhuwei Cao, Jian Liu, Sheng Ye

**Affiliations:** ^1^ Agricultural Photocatalysis Laboratory School of Materials and Chemistry Anhui Agricultural University Hefei 230036 China; ^2^ Inner Mongolia Key Laboratory of Rare Earth Catalysis, School of Chemistry and Chemical Engineering Inner Mongolia University Hohhot Inner Mongolia 010021 China; ^3^ DICP‐Surrey Joint Centre for Future Materials University of Surrey Guildford Surrey GU2 7XH UK

**Keywords:** electrocatalytic hydrogen evolution, Mott‐Schottky, nanoreactors, phase junctions, Sn‐S_2_‐Mo

## Abstract

The electrocatalytic hydrogen evolution reaction (HER) plays a pivotal role in electrochemical energy conversion and storage. However, traditional HER catalysts still face significant challenges, including limited activity, poor acid resistance, and high costs. To address these issues, a hollow core‐shell structured 2H@1T‐MoS_2_‐Sn_1_ nanoreactor is designed for acidic HER, where Sn single atoms are anchored on the shell of 2H@1T‐MoS_2_ Mott‐Schottky phase junction. The 2H@1T‐MoS_2_‐Sn_1_ catalyst demonstrates exceptional HER performance, achieving an ultralow overpotential of 9 mV at 10 mA cm^−2^ and a Tafel slope of 16.3 mV dec^−1^ in acidic media—the best performance reported to date among MoS_2_‐based electrocatalysts. The enhanced performance is attributed to the internal electric field at the Mott‐Schottky phase junction, which facilitates efficient electron transfer. Additionally, the Sn single atoms modulate the electronic structure of Mo atoms within the Sn‐S_2_‐Mo motif, inducing a significant shift in the d‐band center and thereby optimizing the dehydrogenation process. This work presents a novel electrocatalyst design strategy that simultaneously engineers interfacial charge transfer and surface catalysis, offering a promising approach for advancing energy conversion technologies.

## Graphical abstract

A hollow core‐shell structured Mott‐Schottky phase junction 2H@1T‐MoS_2_‐Sn_1_ nanoreactor with a definite Sn‐S_2_‐Mo motif is designed, which exhibits a record overpotential of 9 mV at 10 mA cm^−2^. The 2H@1T‐MoS_2_ Mott‐Schottky phase junction promotes charge transfer, while the surface Sn single atom facilitates the reduction of adsorbed H⁺, thus accelerating the catalytic performance.

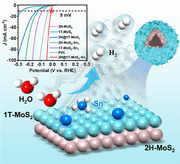

## 1 Introduction

Hydrogen, with its high energy density and environmental friendliness, has emerged as a key player in advancing green energy technologies and achieving carbon neutrality.^[^ ^1^ ^]^ Developing hydrogen energy is thus a critical strategy for sustainable energy solutions. The hydrogen evolution reaction (HER) via electrochemical water splitting is a promising approach for hydrogen production.^[^ ^2^ ^]^ Acidic media are particularly advantageous for water electrolysis due to their higher ionic conductivity and faster hydrogen reaction kinetics,^[^ ^3^ ^]^ which enable higher current densities compared to alkaline media.^[^ ^4^ ^]^ Although noble metal‐based catalysts are considered the benchmark for HER in acidic media, their scarcity and high cost hinder widespread commercial adoption.^[^ ^5^ ^]^ Therefore, the development of cost‐effective, highly active electrocatalysts for acidic HER is essential for advancing the hydrogen economy.^[^ ^6^ ^]^

Molybdenum disulfide (MoS_2_) has emerged as a highly promising alternative to platinum‐based catalysts for HER.^[^ ^7^ ^]^ MoS_2_ exists primarily in two phases: the thermodynamically stable 2H (hexagonal) phase and the metallic 1T (trigonal) phase.^[^ ^8^ ^]^ While the 2H phase exhibits high stability, its HER performance is limited by poor charge transfer capabilities.^[^ ^9^ ^]^ In contrast, the 1T phase demonstrates superior charge transport properties but suffers from relatively low catalytic activity compared with noble metal‐based catalysts.^[^ ^5^, ^10^ ^]^ To address these limitations, various strategies have been explored, including element doping^[^ ^11^ ^]^ and the construction of hetero/phase junctions.^[^ ^12^ ^]^ Among these, Mott‐Schottky hetero/phase junctions, formed between metals and semiconductors, have garnered significant attention due to their ability to generate a space charge region and facilitate spontaneous charge transfer.^[^ ^13^ ^]^ However, despite their excellent charge transfer capabilities, the weak surface catalysis of Mott‐Schottky junctions often limits HER efficiency. Single‐atom catalysts with tunable coordination environments and high atomic utilization show exceptional catalytic activity. Yet the high cost and limited economic viability of noble metal single‐atom catalysts, combined with the relatively poor catalytic activity of transition metal single‐atom catalysts, necessitate the exploration of alternatives with low cost and strong catalytic performance, which offer a promising solution to this challenge.^[^ ^14^ ^]^

In this study, we construct a hollow core‐shell structured Mott‐Schottky phase junction, 2H@1T‐MoS_2_‐Sn_1_, by anchoring single Sn atoms on the shell of 2H@1T‐MoS_2_ to form a well‐defined Sn‐S_2_‐Mo structure. The resulting 2H@1T‐MoS_2_‐Sn_1_ nanoreactor demonstrates outstanding HER performance in acidic media, achieving an ultralow overpotential of 9 mV at 10 mA cm^−2^ and a Tafel slope of 16.3 mV dec^−1^, the best performance reported to date among MoS_2_‐based electrocatalysts. Theoretical and experimental analyses reveal that the core‐shell structured 2H@1T‐MoS_2_ Mott‐Schottky phase junction enhances charge transfer, while the Sn‐S_2_‐Mo motif facilitates the reduction of adsorbed H⁺, significantly accelerating HER kinetics. This work provides a novel strategy for designing efficient HER electrocatalysts by synergistically optimizing charge transfer and surface catalysis.

## 2 Results and Discussion

### 2.1 Synthesis and Characterization of Core‐Shell Structured 2H@1T‐MoS_2_‐Sn_1_ Nanoreactor

**Figure 1:**
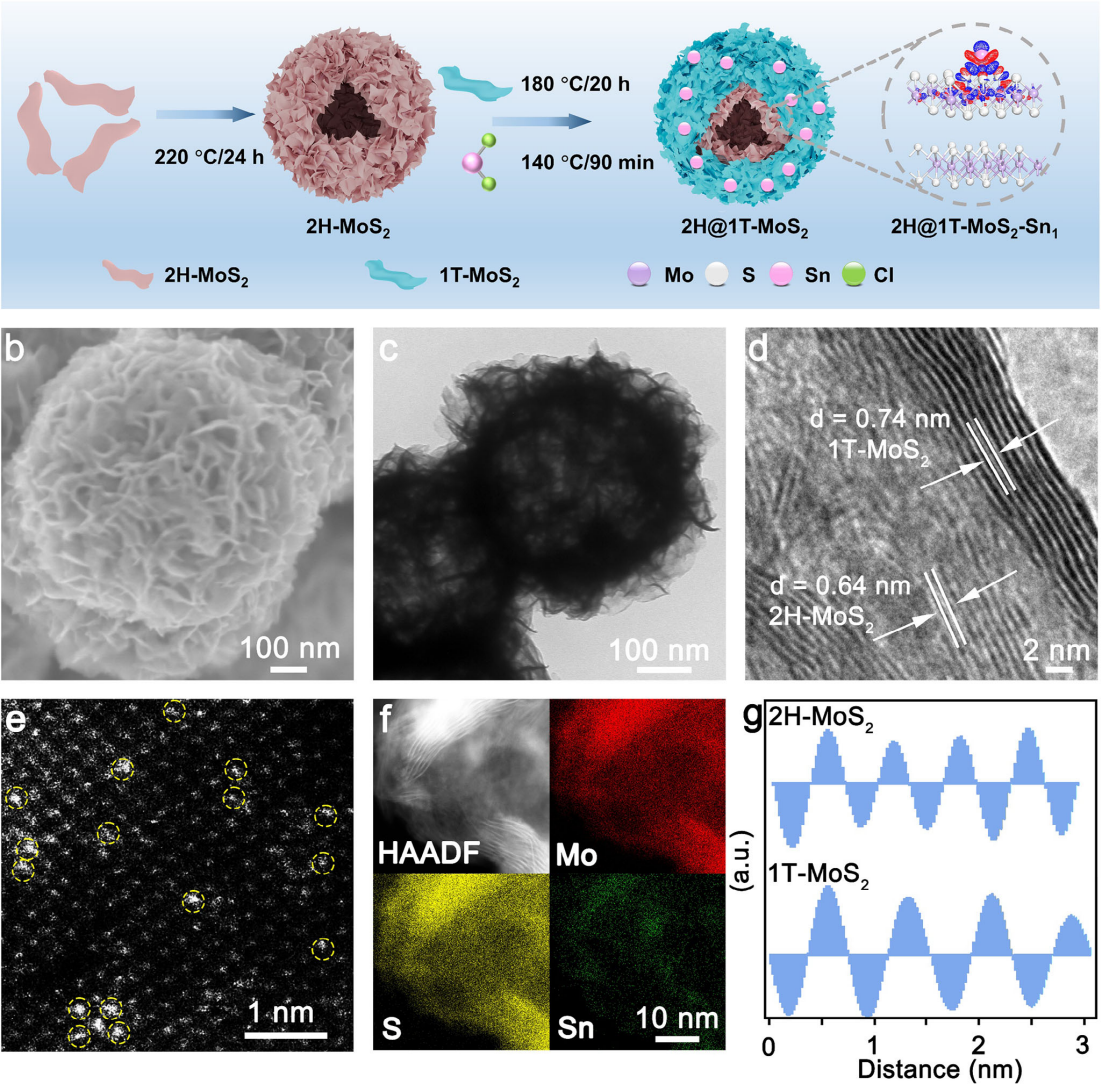
Synthesis and morphology characterization of 2H@1T‐MoS_2_‐Sn_1_. a) Synthesis process of 2H@1T‐MoS_2_‐Sn_1_. b) SEM, c) TEM, d) HRTEM, e) AC‐TEM, f) HAADF and EDS mapping of 2H@1T‐MoS_2_‐Sn_1_, g) Corresponding to different lattice spacing of 2H‐MoS_2_ and 1T‐MoS_2_.

The synthesis process of the core‐shell structured 2H@1T‐MoS_2_‐Sn_1_ nanoreactor, as illustrated in **Figure** **1**a, involves two primary steps. First, a hollow core‐shell structured 2H@1T‐MoS_2_ Mott‐Schottky phase junction is fabricated using the hydrothermal method. Next, Sn single atoms are deposited on the shell of 2H@1T‐MoS_2_ through physical impregnation followed by low temperature calcination. Figure 1b,c show that the 2H@1T‐MoS_2_‐Sn_1_ nanoreactor exhibits a hollow nanoflower ball structure, with lateral dimension ranging from 400–600 nm (Figures S1–S12, Supporting Information).

In Figure 1d,g, the high‐resolution transmission electron microscopy (HRTEM) images and fast Fourier transforms (FFT) reveal the presence of two distinct lattice regions, exhibiting well‐defined interlayer distances of 0.64 nm and 0.74 nm, which correspond to the (002) lattice planes of 2H‐MoS_2_ and 1T‐MoS_2_, respectively. In Figure 1e, aberration‐corrected transmission electron microscopy (AC‐TEM) image reveals distinct bright spots upon the 2H@1T‐MoS_2_ surface, indicating that Sn atoms are in an evenly monodisperse state. As shown in Figure 1f, high‐angle annular dark‐field (HAADF) images and energy‐dispersive spectroscopy (EDS) analysis confirm the homogeneous distribution of S, Mo, and Sn atoms throughout 2H@1T‐MoS_2_‐Sn_1_ nanoreactor. The weight percentage of Sn element in 2H@1T‐MoS_2_‐Sn_1_ nanoreactor is measured to be 1.60% using inductively coupled plasma mass spectrometry (ICP‐MS) (Table S1, Supporting Information).

X‐ray diffraction (XRD) pattern of 2H@1T‐MoS_2_‐Sn_1_ nanoreactor shows two peaks at 32.6°and 9.16°, which correspond to the (100) lattice plane of 2H‐MoS_2_ and the (001) lattice plane of 1T‐MoS_2_, respectively. Furthermore, the Raman spectrum of 2H@1T‐MoS_2_‐Sn_1_ nanoreactor shows two peaks at 380 and 403 cm^−1^ corresponding to the E^1^_2g_ and A_1g_ vibration modes of 2H‐MoS_2_. Prominent peaks at 147, 234, 284, and 336 cm^−1^ are associated with the J_1_, J_2_, E_1g_, and J_3_ modes of 1T‐MoS_2_ (Figure S13, Supporting Information). The results indicate the successful synthesis of 2H@1T‐MoS_2_‐Sn_1_ nanoreactor.

### 2.2 Electronic Structure Characterization of 2H@1T‐MoS_2_‐Sn_1_ Nanoreactor

**Figure 2:**
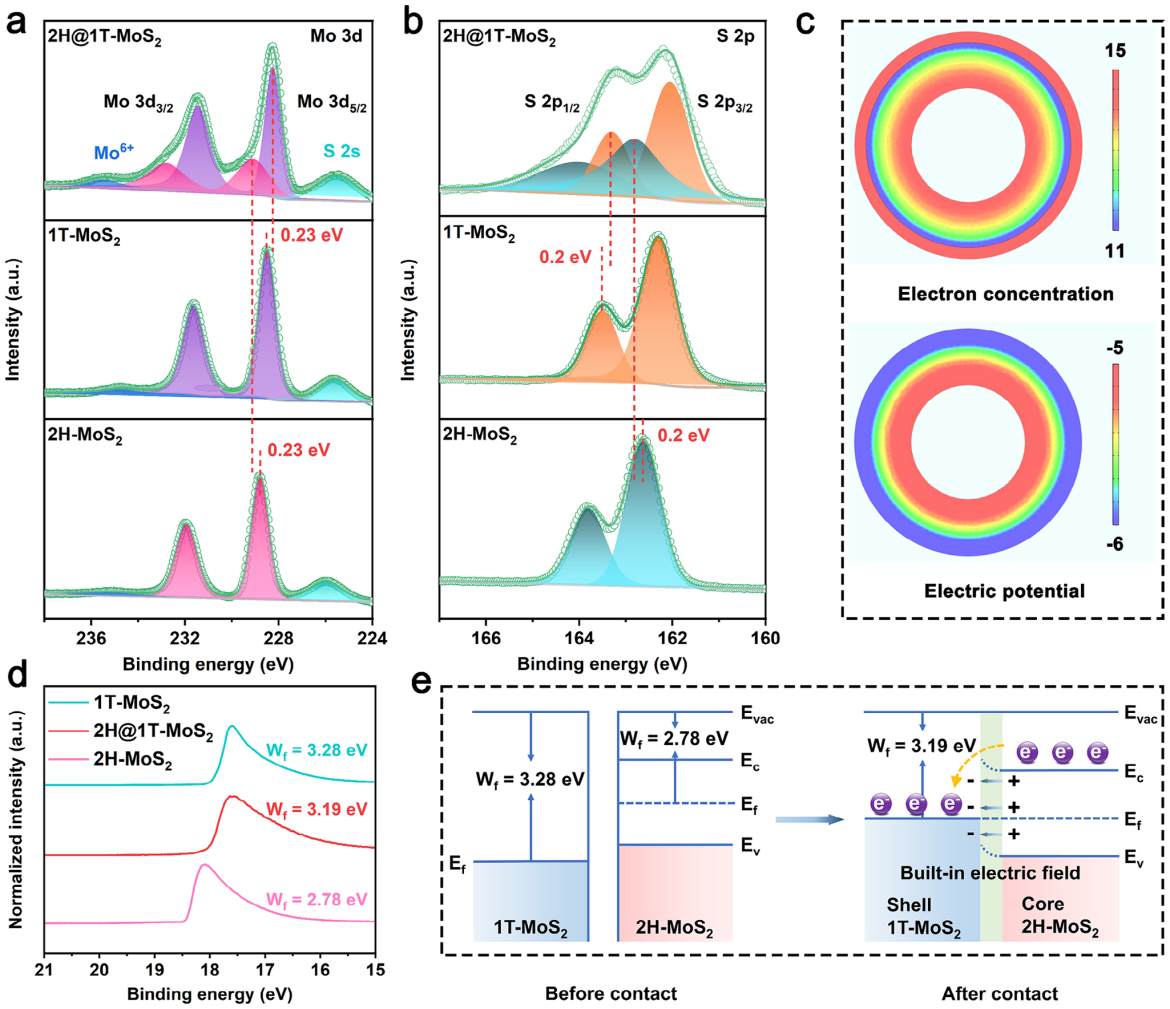
Core‐shell structured Mott‐Schottky phase junction. a) Mo 3d and b) S 2p XPS of the 2H‐MoS_2_, 1T‐MoS_2_ and 2H@1T‐MoS_2_. c) Electron concentration (graph above) and electric potential (graph below) differences of 2D electric field distribution on 2H@1T‐MoS_2_. d) UPS of 2H‐MoS_2_, 1T‐MoS_2_, and 2H@1T‐MoS_2_ in the cutoff energy region. e) Band diagram of n‐type semiconductor 2H‐MoS_2_ and metalic 1T‐MoS_2_ before and after contact.

To investigate the transfer path of electron between 2H‐MoS_2_ and 1T‐MoS_2_, shown in **Figure** **2**a,b, X‐ray photoelectron spectroscopy (XPS) shows that the Mo 3d peak contains four peaks at 228.8 eV, 231.9 eV, 228.5 eV, and 231.6 eV, corresponding to Mo^4+^ 3d_5/2_ and 3d_3/2_ of the 2H phase and 1T phase. In 2H@1T‐MoS_2_, Mo 3d peak of the 2H phase shifts to higher energy (0.23 eV), while Mo 3d peak of the 1T phase shifts to lower energy (0.23 eV). In the S 2p spectrum, the S 2p peak of the 2H phase exhibits a positive shift (0.2 eV), while the 1T phase manifests a negative shift (0.2 eV). Such changes imply electron transfer from 2H‐MoS_2_ to 1T‐MoS_2_.^[^ ^15^ ^]^

To understand the Mott‐Schottky phase junction effect on the local electric field, finite element simulations (FEA) are carried out to analyze the 2D electric field distribution of core‐shell structured 2H@1T‐MoS_2_ by utilizing the COMSOL multiphysics solver. Figure 2c illustrates that the local charge density on the outer surface of 1T‐MoS_2_ is considerably higher than that of 2H‐MoS_2_, and the electric potential of 1T‐MoS_2_ is lower than that of 2H‐MoS_2_, both of which indicate the transfer of electrons from the 2H phase to the 1T phase. Meanwhile, ultraviolet photoelectron spectroscopy (UPS) is used to determine the work function (WF), which signifies the minimal energy necessary for electron transfer from the Fermi level to the vacuum level (Figures S14 and S15, Supporting Information).

In Figure 2d, the WF of 2H‐MoS_2_ (2.78 eV) is relatively lower than that of 1T‐MoS_2_ (3.28 eV). Energy difference is formed between two phases, leading to electron transfer from 2H‐MoS_2_ to 1T‐MoS_2_.^[^ ^16^ ^]^ Figure 2e shows the correlation between metalic WF and semiconductor energy band (Figure S16, Supporting Information). Charge redistribution occurs at the interface between 1T‐MoS_2_ and 2H‐MoS_2_ upon contact, appearing positive charges in 2H‐MoS_2_ and negative charges in 1T‐MoS_2_, with downward bending of the energy band and the formation of Mott‐Schottky contact.^[^ ^17^ ^]^ Such establishment of space charge region can facilitate the directional motion of electrons. The built‐in electric field (BEF) within 2H@1T‐MoS_2_‐Sn_1_ is systematically investigated by Kelvin probe force microscopy (KPFM) and Zeta potential measurement. The KPFM analysis discloses that 2H@1T‐MoS_2_‐Sn_1_ attains a maximum surface potential of 276.5 mV. This value substantially surpasses those of its counterparts, namely 2H@1T‐MoS_2_ (235.0 mV), 1T‐MoS_2_ (109.1 mV), and 2H‐MoS_2_ (85.7 mV). The trend observed in KPFM results is further corroborated by Zeta potential measurement. Among all the materials tested, 2H@1T‐MoS_2_‐Sn_1_ displays the most negative Zeta potential value (− 40.6 mV). Collectively, the KPFM outcomes and Zeta potential values clearly demonstrate that the BEF in 2H@1T‐MoS_2_‐Sn_1_ is capable of enhancing the driving force for electron transport (Figures S17–S19, Supporting Information).^[^ ^18^ ^]^

### 2.3 Coordination Environment Confirmation of 2H@1T‐MoS_2_‐Sn_1_ Nanoreactor

**Figure 3:**
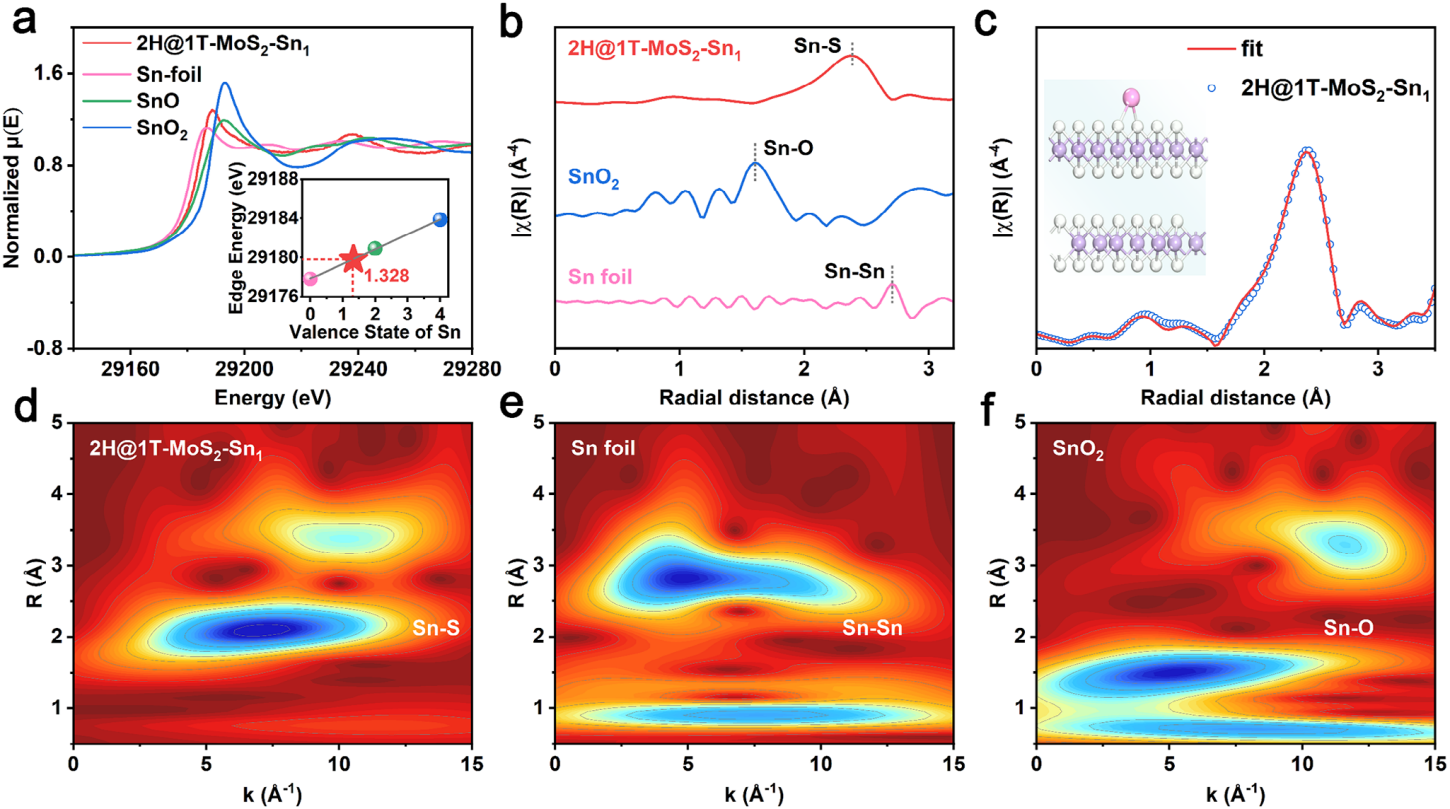
Structure information of 2H@1T‐MoS_2_‐Sn_1_ nanoreactor. a) Normalized K‐edge XANES of Sn, b) EXAFS spectra of Sn K‐edge, c) fitting curves of the EXAFS spectrum of Sn in the R space, d–f) WT of Sn K‐edge.

To comprehend the local microstructure of 2H@1T‐MoS_2_‐Sn_1_ nanoreactor, X‐ray absorption near‐edge structures (XANES) and extended X‐ray absorption fine structures (EXAFS) are conducted to analyze the absorption edges of 2H@1T‐MoS_2_‐Sn_1_ nanoreactor, as well as Sn foil, SnO, and SnO_2_. In **Figure** **3**a, the absorption edge of the 2H@1T‐MoS_2_‐Sn_1_ nanoreactor is positioned between that of Sn foil and SnO, suggesting that the valence state of Sn in 2H@1T‐MoS_2_‐Sn_1_ nanoreactor is between 0 and + 2. In addition, the Sn 3d_5/2_ XPS peak also illustrates the mixed valence nature of Sn, which is consistent with XANES results (Figure S20, Supporting Information). Based on the linear relationship between valence state and absorbed edge energy, the exact valence state of Sn in 2H@1T‐MoS_2_‐Sn_1_ nanoreactor is determined to be + 1.328.^[^ ^19^ ^]^

XPS of 2H@1T‐MoS_2_‐Sn_1_ nanoreactor shows that the anchoring of Sn on 2H@1T‐MoS_2_ induces a positive shift of 0.2 eV in the binding energy of Mo and S. These results suggest a decrease in electron density on the 2H@1T‐MoS_2_ substrate and electron transfer from MoS_2_ to the Sn (Figure S21, Supporting Information). In Figure 3b, the Fourier‐transform EXAFS (FT‐EXAFS) shows that Sn exists in the form of single atoms instead of SnO_2_ and Sn nanoparticles. Meanwhile, the corresponding correlation between K space and R space is plotted to validate the chemical arrangements. The fitting results indicate that the Sn atom coordinates with the S_2_‐Mo structure to form Sn‐S_2_‐Mo motif. Meanwhile, the synchrotron radiation photoemission spectroscopy of 2H@1T‐MoS_2_‐Sn_1_ further reveals the formation of Sn‐S bonds (Figure S22, Supporting Information). As shown in Figure 3c, the bond length of S‐Sn in 2H@1T‐MoS_2_‐Sn_1_ nanoreactor is 2.556 Å. In Figure 3d–f, the wavelet transforms present the structure information of 2H@1T‐MoS_2_‐Sn_1_ nanoreactor and support the presence of Sn‐S bonds, indicating the coordination between Sn and S atoms. Further analysis of S K‐edge data reveals that the introduction of Sn causes a + 0.3 eV shift in the absorbed energy of the S K‐edge. This is due to the redistribution of electron density induced by Sn‐S bonds, where Sn accepts electrons and S loses electrons, resulting in a positive shift of the S K‐edge (Figure S23, Supporting Information).

In order to clarify the local chemical environment, we systematically investigate the hydrogen distribution and acidity properties of the 2H@1T‐MoS_2_‐Sn_1_ nanoreactor surface. Time‐of‐flight secondary ion mass spectrometry and reflection electron energy loss spectroscopy reveal a relatively low hydrogen distribution (≈10%) in both 2H@1T‐MoS_2_ and 2H@1T‐MoS_2_‐Sn_1_ (Figures S24 and S25, Supporting Information). Combined with hydrogen nuclear magnetic resonance and isotopic tracing results, this hydrogen content is attributed to surface‐adsorbed hydroxyl groups and residual amino groups from the synthetic precursors, indicating that protonic acid sites have no contribution to the catalytic system (Figures S26 and S27, Supporting Information). Ammonia temperature‐programed desorption measurements show that, compared with the 2H@1T‐MoS_2_, the accessible acid site density of the 2H@1T‐MoS_2_‐Sn_1_ nanoreactor is significantly reduced, which is attributed to that the introduction of Sn atoms reduces the Lewis acidity of the Mo center (Figure S28, Supporting Information).

### 2.4 Electrocatalytic HER Performance in Acidic Media

**Figure 4:**
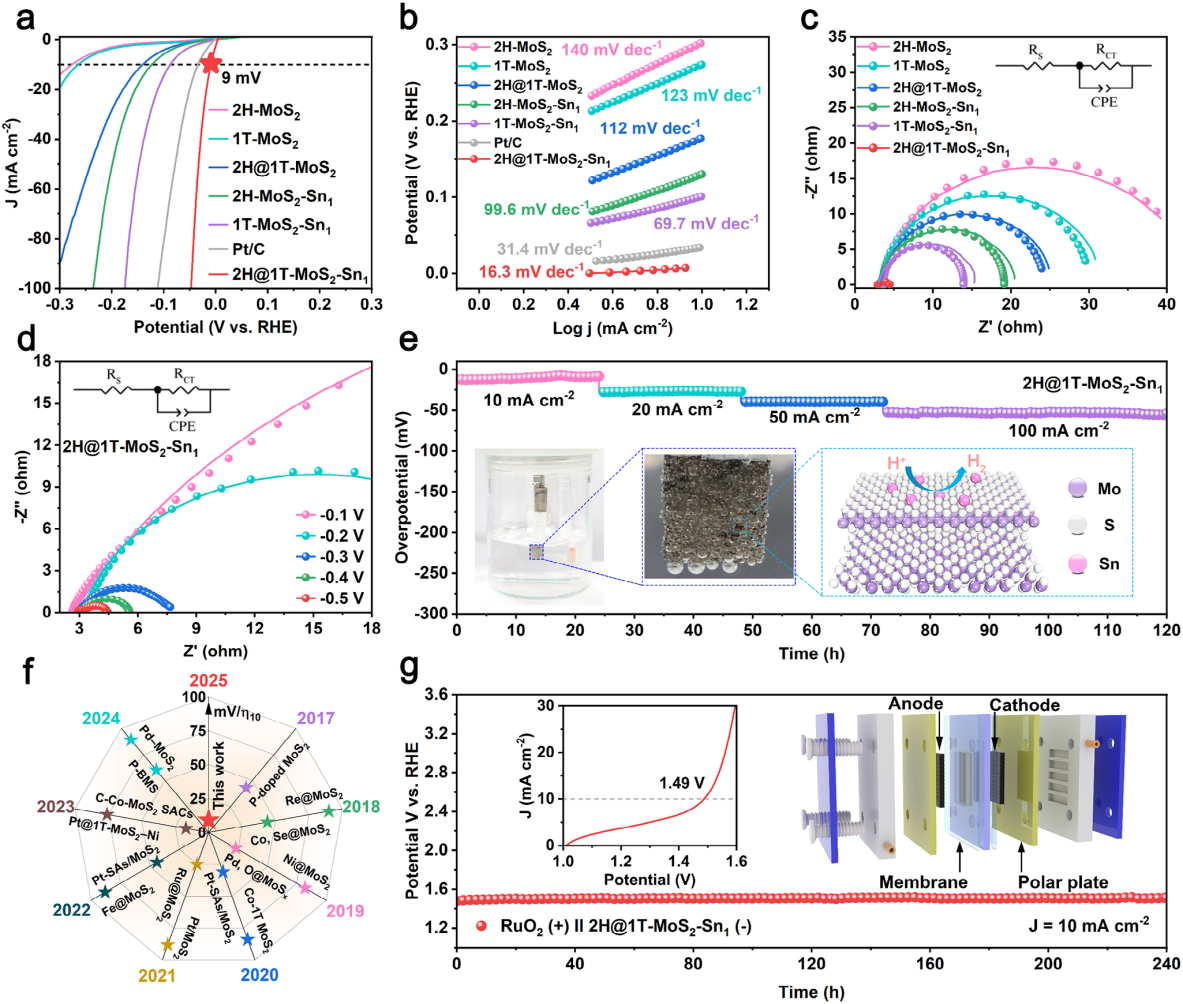
Electrochemical measurement of nanoreactors in acid. a) LSV curves of HER, b) Tafel diagram, c) Nyquist diagram, d) Nyquist diagram of 2H@1T‐MoS_2_‐Sn_1_ nanoreactor at different voltages, e) Temporal potential responses of 2H@1T‐MoS_2_‐Sn_1_ nanoreactor at different current densities, f) Comparison of overpotentials for MoS_2_‐based electrocatalysts, g) PEM device performance (10 mA cm^−2^) using 2H@1T‐MoS_2_‐Sn_1_ nanoreactor and as commercial RuO_2_ catalysts in the PEM electrolyser. Inset: Polarization curve of the overall water splitting.

In a typical three‐electrode setup, we investigated the electrochemical performance of 2H@1T‐MoS_2_‐M_1_ (M stands for single atom = Fe, Co, Ni, V, Mn, Lu, Sm, Er, Dy, Gd, Pt, Ru, Ir, Pd, Rh, Sn) for HER in a 0.5 m H_2_SO_4_ solution. It is found that the performance of Sn single atoms is the best among many single atoms (Figures S29–S35, Supporting Information). In **Figure** **4**a, 2H@1T‐MoS_2_‐Sn_1_ nanoreactor exhibits an impressive overpotential of 9 mV at 10 mA cm^−2^, surpassing commercially available Pt/C catalysts. To our best knowledge, Figure 4f shows that the HER performance of 2H@1T‐MoS_2_‐Sn_1_ nanoreactor is superior to all MoS_2_‐based electrocatalysts reported previously (Tables S2–S4 Supporting Information). The 2H@1T‐MoS_2_‐Sn_1_ nanoreactor achieves a Tafel slope (16.3 mV dec^−1^) in Figure 4b, which is lower than Pt/C catalyst, indicating a faster kinetic process.

Electrochemical impedance spectroscopy (EIS) is employed to probe the charge transfer dynamics involved in HER processes. The results in Figure 4c reveal a reduction in charge transfer resistance (R_ct_) from 20.94 to 1.42 Ω cm^2^ (Table S5 Supporting Information). In situ EIS analysis reveals the adsorption/desorption kinetics of intermediates at Sn active sites under varying potentials. The 2H@1T‐MoS_2_‐Sn_1_ nanoreactor exhibits gradually decreased Nyquist semicircles between − 0.1 V and − 0.5 V, demonstrating enhanced reactant adsorption capacity during HER, as depicted in Figure 4d.^[^ ^20^ ^]^ Bode analysis distinguishes interfacial charge transfer (low‐frequency) from inner‐layer electron transport (high‐frequency). The rapid phase angle attenuation of the 2H@1T‐MoS_2_‐Sn_1_ at low frequencies confirms accelerated charge transfer dynamics that effectively promote HER kinetics (Figure S36, Supporting Information). These results confirm effective charge transfer at the interface and validate rapid electron transfer and HER kinetics.^[^ ^21^ ^]^

**Figure 5:**
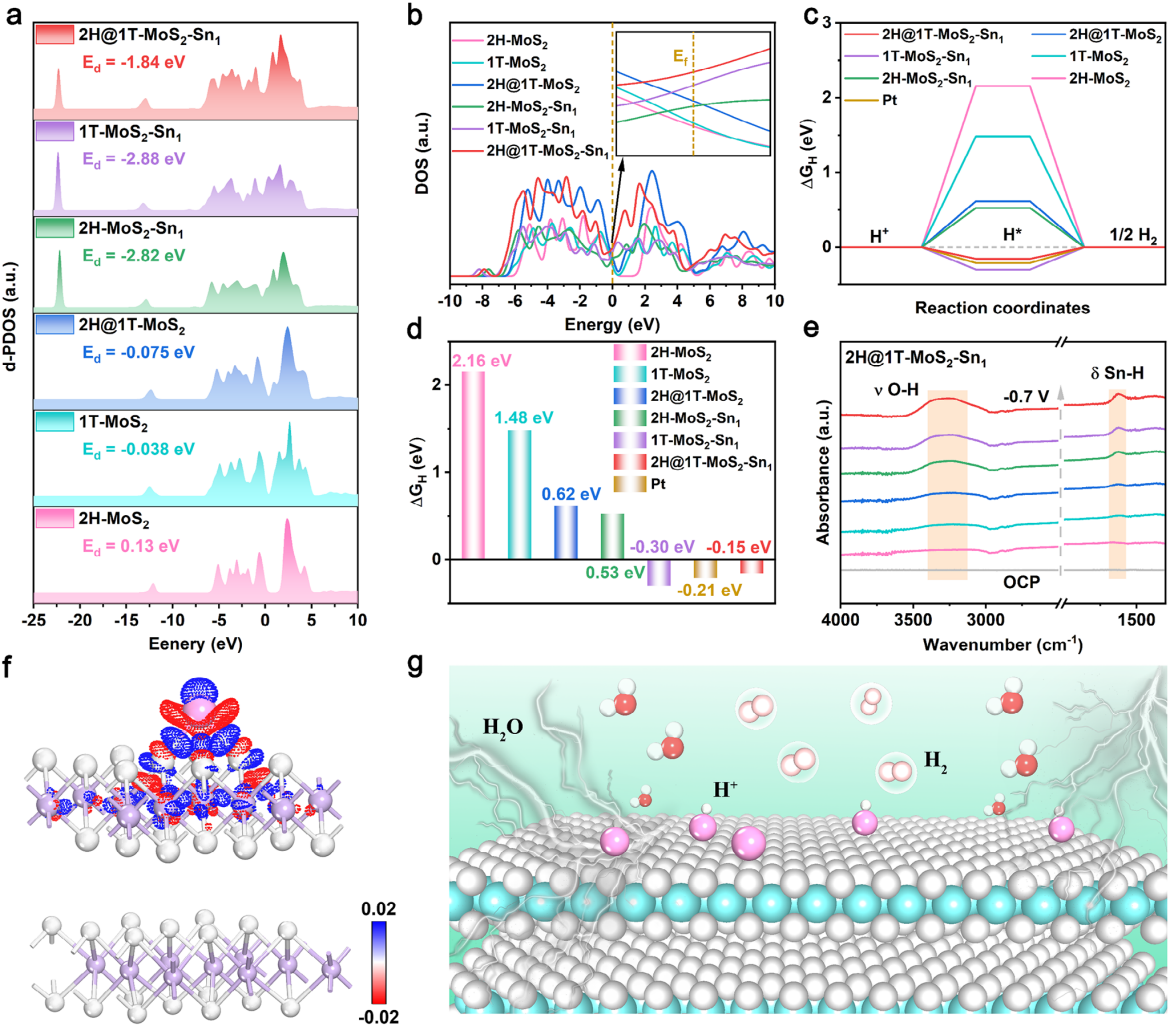
Theoretical calculation of HER. a) PDOS diagram, b) TDOS diagram, c) Adsorption Gibbs free energy of H*, and d) free energy comparison for the 2H‐MoS_2_, 1T‐MoS_2_, 2H@1T‐MoS_2_, 2H‐MoS_2_‐Sn_1_, 1T‐MoS_2_‐Sn_1_, and 2H@1T‐MoS_2_‐Sn_1_. e) The in situ infrared spectra of 2H@1T‐MoS_2_‐Sn_1_ nanoreactor at various applied cathodic potentials during acidic HER. f) EDDs structure and g) surface reaction mechanism diagram of 2H@1T‐MoS_2_‐Sn_1_ nanoreactor.

The intrinsic activity of the HER is evaluated using cyclic double‐layer capacitance (C_dl_) and electrochemical active surface area (ECSA) analyses (Figures S37–S39, Supporting Information). The C_dl_ value for the 2H@1T‐MoS_2_‐Sn_1_ nanoreactor is 13.5 mF cm^−2^, which is 2.4‐fold that of 2H@1T‐MoS_2_, manifesting a significant enhancement in catalytic activity. 2H@1T‐MoS_2_‐Sn_1_ nanoreactor exhibits a higher turnover frequency (TOF) of 0.5 s^−1^ at 50 mV, surpassing the performance of other samples (Figure S40, Supporting Information). The 2H@1T‐MoS_2_‐Sn_1_ nanoreactor is continuously tested at 10, 20, 50, and 100 mA cm^−2^ to investigate the durability. Figure 4e shows only slight decay in overpotential after continuous operation for 120 h, confirming excellent stability. In addition, the proton exchange membrane (PEM) electrolyzer is assembled by integrating 2H@1T‐MoS_2_‐Sn_1_ nanoreactor as a cathodic catalyst for HER with commercial RuO_2_ as an anodic catalyst for OER. It is observed that the battery voltage of 2H@1T‐MoS_2_‐Sn_1_ electrode is 1.49 V at 10 mA cm^−2^ (**Figure** **5**g, inset), and there is no significant change in overpotential during 240 h test at 10 mA cm^−2^, indicating the potential of practical application in Figure 4g. Furthermore, a series of characterizations such as XRD, SEM, TEM and XPS analysis after the cycling reaction and in situ XRD investigation during the reaction process confirm the superior stability of 2H@1T‐MoS_2_‐Sn_1_ nanoreactor (Figures S41–S45, Supporting Information). The overpotential shift at 10 mA cm^−2^ is merely 2 mV after cycling, while the difference at 50 mA cm^−2^ is limited to 1 mV. Even at a high current density of 100 mA cm^−2^, the overpotential remains nearly identical to the initial value (Figure S46, Supporting Information). These results collectively demonstrate the exceptional stability of 2H@1T‐MoS_2_‐Sn_1_ nanoreactor.

### 2.5 HER Mechanism Exploration

DFT calculations are employed to assess electronic structures, Sn‐S_2_‐Mo sites, and kinetics process in the 2H@1T‐MoS_2_‐Sn_1_ nanoreactor. In Figure 5a, the electronic structure evolution is analyzed by using predicted partial state densities (PDOS), where the d‐band center serves as a descriptor to assess the interaction between Sn single atoms and 2H@1T‐MoS_2_. The d‐band center value for the 2H@1T‐MoS_2_‐Sn_1_ nanoreactor exhibits a significant downward change compared to the 2H@1T‐MoS_2_.

The PDOS suggests that upon introduction of Sn atoms, the 2H@1T‐MoS_2_‐Sn_1_ nanoreactor has an increased electron occupancy in the antibonding state, leading to a decreased affinity for H* adsorption and the enhancement of the dehydrogenation process.^[^ ^3^, ^22^ ^]^ The placement of values in the 2H@1T‐MoS_2_‐Sn_1_ nanoreactor (− 1.84 eV) falls between those of the 2H‐MoS_2_‐Sn_1_ nanoreactor (− 2.82 eV) and the 1T‐MoS_2_‐Sn_1_ nanoreactor (− 2.88 eV), indicating a moderate capability for adsorbing active intermediates. Moreover, the total density of states (TDOS) indicates that introducing Sn single atoms can elevate the carrier density of the material as depicted in Figure 5b.

We calculated the H* free adsorption energy (ΔG_H_), a key parameter of HER activity of different catalysts in acidic media. In Figure 5c,d, the value of ΔG_H_ for the 2H@1T‐MoS_2_‐Sn_1_ nanoreactor is − 0.15 eV, significantly lower than 2H‐MoS_2_ (2.16 eV), 1T‐MoS_2_ (1.48 eV), and 2H@1T‐MoS_2_ (0.62 eV), displaying that the presence of Sn enhances the desorption of H_2_ on 2H@1T‐MoS_2_. Remarkably, the free energy of the 2H@1T‐MoS_2_‐Sn_1_ nanoreactor is closer to zero than that of Pt, indicating its superior ability in H_2_ desorption compared to Pt. Examination of the electronic structure is performed to elucidate the atomic interactions during the reaction, as illustrated in Figure 5f. The electron density difference (EDD) analysis shows that charge accumulation occurs around Sn single atom, and charge loss occurs around Mo atom, indicating that the introduction of Sn leads to the redistribution of electrons on 2H@1T‐MoS_2_‐Sn_1_ nanoreactor and the directional transfer of electrons from Mo to S and then to Sn. This leads to a greater electron concentration for Sn with strong reduction ability, thereby aiding in the H^+^ reduction.

To further explore the catalytic process of 2H@1T‐MoS_2_‐Sn_1_ nanoreactor in acidic HER, in situ attenuated total reflection surface‐enhanced infrared absorption spectroscopy (ATRSEIRAS) is used to monitor the produced intermediates. As shown in Figure 5e, the 3200–3600 cm^−1^ broad peak attributed to the stretching (ν O‐H) mode of interfacial water molecules, enhances with the increase of the applied bias, indicating enhanced H_2_O adsorption, consistent with the Volmer step. Moreover, a new small peak at ≈1 621 cm^−1^, attributed to the Sn‐H stretching vibration (δ Sn‐H),^[^ ^23^ ^]^ verifies that Sn atoms are more likely to adsorb H* during acidic hydrogen production. The HER mechanism of the 2H@1T‐MoS_2_‐Sn_1_ nanoreactor in acidic electrolytes is depicted in Figure 5g. In general, the DFT calculations indicate that the 2H@1T‐MoS_2_‐Sn_1_ nanoreactor could serve as a superior catalyst for acidic electrochemical HER.

## 3 Conclusion

In summary, we successfully constructed a hollow core‐shell structured 2H@1T‐MoS_2_‐Sn_1_ nanoreactor by anchoring single Sn atoms on the shell of a Mott‐Schottky phase junction. The 2H@1T‐MoS_2_‐Sn_1_ nanoreactor demonstrates exceptional performance for acidic HER, achieving an ultralow overpotential of 9 mV at 10 mA cm^−2^ and a Tafel slope of 16.3 mV dec^−1^. Experimental and theoretical analyses confirm that the core‐shell structured 2H@1T‐MoS_2_ Mott‐Schottky phase junction facilitates efficient electron transfer, while the Sn single atoms bridge neighboring S atoms connected to inner‐layer Mo atoms, forming a Sn‐S_2_‐Mo motif. This unique structural configuration optimizes water dissociation and H* adsorption, significantly enhancing electrocatalytic performance. This work provides a novel strategy for designing high‐performance electrocatalysts by simultaneously engineering interfacial charge transfer and surface catalysis, offering new insights for advancing acidic hydrogen evolution technologies.

## 4 Experimental Section

Experimental details are provided in the Supporting Information.

## Conflict of Interest

The authors declare no conflict of interest.

## Supporting information



Supporting Information

## Data Availability

The data that support the findings of this study are available from the corresponding author upon reasonable request.
