# Supplementary material for: Atomically Dispersed Sn on Core‐Shell MoS2 Nanoreactors as Mott‐Schottky Phase Junctions for Efficient Electrocatalytic Hydrogen Evolution
Source: Adv Mater. 2025 May 6;37(33):2502977. doi: 10.1002/adma.202502977 (PMC12369680; doi:10.1002/adma.202502977)
Supplement: Supplementary file 1 — Supporting Information [file ADMA-37-2502977-s001.docx]

Supporting Information

**Atomically Dispersed Sn on Core-Shell MoS_2_ Nanoreactors as Mott-Schottky Phase Junctions for Efficient Electrocatalytic Hydrogen Evolution**

*Hao Jin, Yan Zhang, Zhuwei Cao, Jian Liu^*^, Sheng Ye^*^*

# Table of Contents

## 1.Experimental Section.

### 1.1 Materials.

### 1.2 Synthesis of 2H-MoS_2_.

### 1.3 Synthesis of 1T-MoS_2_.

### 1.4 Synthesis of 2H@1T-MoS_2_.

### 1.5 Synthesis of 2H@1T-MoS_2_-Sn_1_ nanoreactor.

### 1.6 Synthesis of 2H-MoS_2_-Sn_1_ and 1T-MoS_2_-Sn_1_.

### 1.7 Synthesis of 2H@1T-MoS_2_-M_1_ nanoreactor.

### 1.8 Characterization measurements.

### 1.9 Fabrication and measurement of the electrodes.

### 1.10 Supporting Figures 1-32

#### Figure S1. 2H-MoS_2_ SEM images.

#### Figure S2. 2H-MoS_2_-Sn_1_ SEM images.

#### Figure S3. 1T-MoS_2_ SEM images.

#### Figure S4. 1T-MoS_2_-Sn_1_ SEM images.

#### Figure S5. 2H@1T-MoS_2_ SEM images.

#### Figure S6. 2H@1T-MoS_2_-Sn_1_ SEM images.

#### Figure S7. 2H-MoS_2_ TEM images.

#### Figure S8. 2H-MoS_2_-Sn_1_ TEM images.

#### Figure S9. 1T-MoS_2_ TEM images.

#### Figure S10. 1T-MoS_2_-Sn_1_ TEM images.

#### Figure S11. 2H@1T-MoS_2_ TEM images.

#### Figure S12. 2H@1T-MoS_2_-Sn_1_ TEM images.

#### Figure S13. XRD and Raman.

#### Figure S14. Valence band image of 2H-MoS_2_.

#### Figure S15. Valence band image of 2H@1T-MoS_2_-Sn_1_.

#### Figure S16. Mott-Schottky plots.

#### Figure S17. KPFM.

#### Figure S18. Surface contact potential difference.

#### Figure S19. Zeta potential.

#### Figure S20. XPS of Sn 3d.

#### Figure S21. XPS of Mo 3d and S 2p.

#### Figure S22. SRPES.

#### Figure S23. Normalized K-edge XANES of S.

#### Figure S24. TOF-SIMS.

#### Figure S25. REELS.

#### Figure S26. ^1^H NMR.

#### Figure S27. D_2_ isotopic labeling experiments.

#### Figure S28. NH_3_-TPD.

#### Figure S29. LSV curves and overpotentials of different 2H@1T-MoS_2_ ratios.

#### Figure S30. LSV curves and Tafel slope diagram of Fe, Co, Ni, V and Mn.

#### Figure S31. LSV curves and Tafel slope diagram of Lu, Sm, Er, Dy, and Gd.

#### Figure S32. XPS of Mo 3d, S 2p, Sn 3d. LSV curves and Tafel slope diagram of Pt, Ru, Ir, Pd and Rh.

#### Figure S33. LSV curves and overpotentials of different contents Sn.

#### Figure S34. LSV curves and overpotentials at different annealing temperatures.

#### Figure S35. LSV curves and overpotentials at different annealing times.

#### Figure S36. Bode phase plots.

#### Figure S37. CV curves.

#### Figure S38. C_dl_ curves.

#### Figure S39. Electrochemically active surface area and normalized curves.

#### Figure S40. TOF values.

#### Figure S41. XRD of 2H@1T-MoS_2_-Sn_1_ reaction.

#### Figure S42. SEM images of 2H@1T-MoS_2_-Sn_1_ reaction.

#### Figure S43. TEM images of 2H@1T-MoS_2_-Sn_1_ reaction.

#### Figure S44. XPS of Mo 3d, S 2p, Sn 3d.

#### Figure S45. In situ XRD pattern.

#### Figure S46. The LSV polarization curves of the 2H@1T-MoS_2_-Sn_1_ nanoreactor before and after cycling test.

### 1.11 Supporting Tables 1-5

#### Table 1. ICP-MS data of Sn.

#### Table 2. Comparison of overpotential and Tafel slope of MoS_2_-based electrocatalysts in acidic solutions.

#### Table 3. Comparison of overpotential and Tafel slope of MoS_2_-based electrocatalysts in neutral solutions.

#### Table 4. Comparison of overpotential and Tafel slope of MoS_2_-based electrocatalysts in alkaline solutions.

#### Table 5. Parameters of EIS.

1.Experimental Section

1.1 Materials

Chemicals: All chemicals can be used directly without further purification. hexahydrate cobalt nitrate (Co(NO_3_)_2_·6H_2_O), hexahydrate nickel nitrate (Ni(NO_3_)_2_·6H_2_O), nonahydrate iron nitrate (Fe(NO_3_)_3_·9H_2_O), vanadium chloride (VCl_3_), manganese chloride tetrahydrate (MnCl_2_·4H_2_O), tin chloride dihydrate (SnCl_2_·2H_2_O), hexahydrate gadolinium nitrate (Gd(NO_3_)_3_·6H_2_O), samarium nitrate (Sm(NO_3_)_3_), hexahydrate dysprosium nitrate (Dy(NO_3_)_3_·6H_2_O), hexahydrate erbium nitrate (Er(NO_3_)_3_·6H_2_O), hexahydrate lutetium nitrate (Lu(NO_3_)_3_·6H_2_O), Chloroplatinic acid (H_2_PtCl_6_), Chloroiridic acid (H_2_IrCl_6_), Ruthenium chloride (RuCl_3_), Palladium chloride (PdCl_2_), Rhodium Chloride (RhCl_3_) hexadecyl trimethyl ammonium bromide (CTAB), anhydrous sodium molybdate (MoNa_2_O_4_), anhydrous glucose (C_6_H_12_O_6_), n-butanol (C_4_H_10_O), ethylene glycol (C_2_H_6_O_2_), ethanol (C_2_H_6_O), concentrated hydrochloric acid (HCl), L-ascorbic acid (C_6_H_8_O_6_), ammonium molybdate tetrahydrate ((NH_4_)_6_Mo_7_O_24_·4H_2_O), thiourea (CS(NH_2_)_2_), all purchased from Sinopharm Chemical Reagent Co. Ltd. (Shanghai, China).

1.2 Synthesis of 2H-MoS_2_

2H-MoS₂ is synthesized by the micelle-constrained microemulsion method. Generally, 0.206 g of MoNa_2_O_4_ and 0.6 g of C_6_H_12_O_6_ are dissolved in 300 mL of H_2_O. Then the aqueous solution is added into an oil solution of CTAB (5.4650 g dissolved in 100 mL of C_4_H_10_O) and stirred for 2 h. 50 mL of C_2_H_6_O_2_ and 0.5 mL of HCl are added in above emulsion and stirred for 1 h. Then, 1.1420 g of CS(NH_2_)_2_ is added and stirred for 3 h. Afterward, the mixture is stored at 220 °C for 24 h, and then precipitation separation is carried out and it is dried at 60 °C for 24 h. Finally, the prepared powder was heat-treated at 900 °C under an Ar atmosphere for 5 h (Anhui Kemi Machinery Technology Co., ltd) to obtain a hollow-structured 2H-MoS_2_.^[1]^

**1.3** **Synthesis of 1T-MoS_2_**

1.236 g of (NH_4_)_6_Mo_7_O_24_·4H_2_O and 1.0657 g of CS(NH_2_)_2_ are dissolved in 40 mL of H_2_O. During stirring, 0.03 g of C_6_H_8_O_6_ is added. The solution is heated to 180 °C for 20 h which moved to high-pressure autoclave (100 mL). After cooling to room temperature, the resulting product is collected by centrifugation, washed several times with C_2_H_6_O and H_2_O to remove impurities, and dried at 60 °C to obtain the black powder.^[49]^

**1.4** **Synthesis of 2H@1T-MoS_2_**

Typically, fully annealed 2H-MoS_2_ is added to the precursor solution of 1T-MoS_2_. Then the black mixture solution is poured into high-pressure autoclave and kept at 180 °C for 20 h. After the resulting product is centrifugated and dried. Finally, under Ar atmosphere, the prepared powder is heat-treated at 400 °C for 3 h to obtain a black powder of 2H@1T-MoS_2_.

**1.5** **Synthesis of 2H@1T-MoS_2_-Sn_1_ nanoreactor**

The mixture consisted of black powder containing 2H@1T-MoS_2_ with a mass of 100 mg. This powder is then added to a solution consisting of C_2_H_6_O_2_ (1 mL) and H_2_O (9 mL). Afterwards, SnCl_2_·2H_2_O is introduced into the solution. The resulting mixture is heated at 60 °C for 10 h. After that, it underwent centrifugation followed by washing and subsequent drying. Finally, the prepared powder went through heat treatment under an Ar atmosphere at 140 °C for 90 min to produce 2H@1T-MoS_2_-Sn_1_ nanoreactor.

**1.6** **Synthesis of 2H-MoS_2_-Sn_1_ and 1T-MoS_2_-Sn_1_**

The synthesis methods mentioned above are similar, with the only distinction being the inclusion of 2H-MoS_2_ and 1T-MoS_2_, respectively.

1.7 **Synthesis of 2H@1T-MoS_2_-M_1_ nanoreactor**

The mixture consisted of black powder containing 2H@1T-MoS_2_ with a mass of 100 mg. This powder is then added to a solution consisting of C_2_H_6_O_2_ (1 mL) and H_2_O (9 mL). Afterwards, Fe(NO_3_)_3_·9H_2_O, Co(NO_3_)_2_·6H_2_O, Ni(NO_3_)_2_·6H_2_O, VCl_3_, MnCl_2_·4H_2_O, Lu(NO_3_)_3_·6H_2_O, Sm(NO_3_)_3_, Er(NO_3_)_3_·6H_2_O, Dy(NO_3_)_3_·6H_2_O, Gd(NO_3_)_3_·6H_2_O, H_2_PtCl_6_, H_2_IrCl_6_, RuCl_3_, PdCl_2_ and RhCl_3_ are used as the salts of Fe, Co, Ni, V, Mn, Lu, Sm, Er, Dy, Gd, Pt, Ir, Ru, Pd, and Rh, respectively, and are introduced into the solution. The resulting mixture is heated at a temperature of 60 °C for a duration of 10 h. After that, it underwent centrifugation followed by washing and subsequent drying in a vacuum drying oven set at 60 °C for 12 h. Finally, the prepared powder went through heat treatment under an Ar atmosphere at 140 °C for 90 min to produce hollow-structured 2H@1T-MoS_2_-M_1_ nanoreactor.

1.8 **Characterization measurements**

The structures of the materials were characterized by using scanning electron microscopy (SEM, S-4800, Japan), transmission electron microscopy (TEM, HT-7700, Japan). high-angle annular dark field (HAADF) and the corresponding energy-dispersive spectroscopy (EDS) mapping analyses were performed on a spherical aberration-corrected transmission electron microscopy (AC-TEM, JEM-ARM200F, Japan) at an acceleration voltage of 200 kV. X-ray diffraction (XRD) was employed to characterized the material structures by using Cu Kα radiation (40 kV, 200 mA, 5° min^-1^) with a powder diffractometer (Rigaku D/Max-2500/PC). In situ XRD system was built through the combination of the electrochemical workstation (CS310M), the D8 Advance, and the three-electrode pool. X-ray photoelectron spectroscopy (XPS) were collected on a Thermo Scientific Escalab (ESCALAB 250Xi, Massachusetts) with monochromatized Al Kα X-ray as the excitation source. Raman scattering spectra (Raman) were measured at room temperature on a Raman spectrometer system (LabRAM HR Evolution, France) with an excitation wavelength of 532 nm in backscattering geometry. The Shanghai Synchrotron Radiation Facility is used to measure the X-ray absorption near-edge structure (XANES) and extended X-ray absorption fine structure (EXAFS). Ultraviolet photoelectron spectroscopy (UPS) measurements are conducted on Thermo Scientific ESCALab 250Xi using a gas discharge lamp with helium gas and the HeI (21.22 eV) emission line was employed.

1.9 **Fabrication and measurement of the electrodes**

All electrochemical measurements are carried out at CS310M electrochemical workstation at room temperature. A working electrode is made by loading the catalyst onto the Ni foam (0.5 × 0.5 cm^2^, 0.5 mL, 2 mg mL^-1^). Next, 30 μL of a Nafion-water solution with a concentration of 0.5 wt.% was evenly distributed across the electrode surface. The reference electrode consisted of a saturated calomel electrode, while the counter electrode is a Pt foil electrode. All polarization curves are measured in a 0.5 M H_2_SO_4_ (pH = 0) solution at scan rate of 5 mV s^-1^. The potentials are calibrated by a reversible hydrogen electrode (RHE). Commercial Pt/C (20 wt.%) is used as the benchmark electrocatalyst. The Closed CV of all catalysts in the scan rate range of 20 ~ 200 mV s^-1^. All EIS curves are measured at the open-circuit voltages. The recorded potentials are adjusted by the equation:

E_RHE_ = E_SCE_ + 0.059 × pH + 0.242.

The values of ECSA are calculated based on previous reports:

$$\text{ESCA = }\frac{\text{C}_{\text{dl}}}{\text{C}_{\text{s}}}$$

Where, the Cs is the specific capacitance in the range of 0.020-0.090 mF cm^-2^. The capacitance of 0.040 mF cm^-2^ was used to calculate the ECSA based on typical reported values.
The TOF was calculated by the following equation:

$$\text{TOF = }\frac{\text{j × A}}{\text{m × F × n}}$$

where, J (mA cm^-2^) is the measured current density at given overpotential; A is the surface area of Ni foam electrode; m is the number of electrons (OER m = 4 and HER m = 2); F is faraday constant (96485 C mol^-1^) and n is the total mole of the metal atoms on the electrode (assuming that every metal atom is involved in the catalysis).

1.10 **Supporting Figures S1-S32**


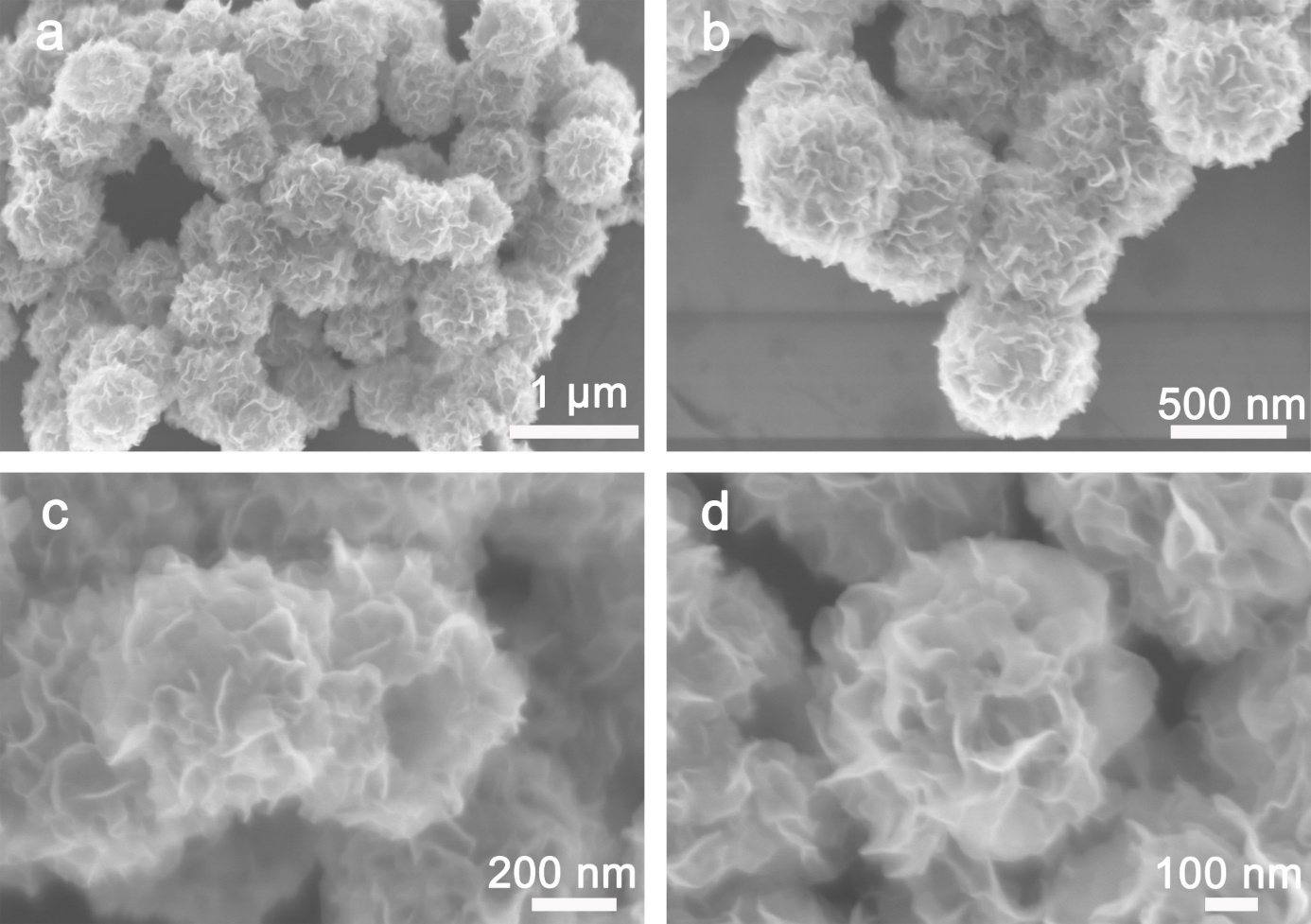


**Figure S1.** (a-d) Scanning electron microscopy (SEM) images of the 2H-MoS_2_ at different magnifications. 2H-MoS_2_ exhibits a nanosphere structure with dimensions ranging from 400 nm to 600 nm.


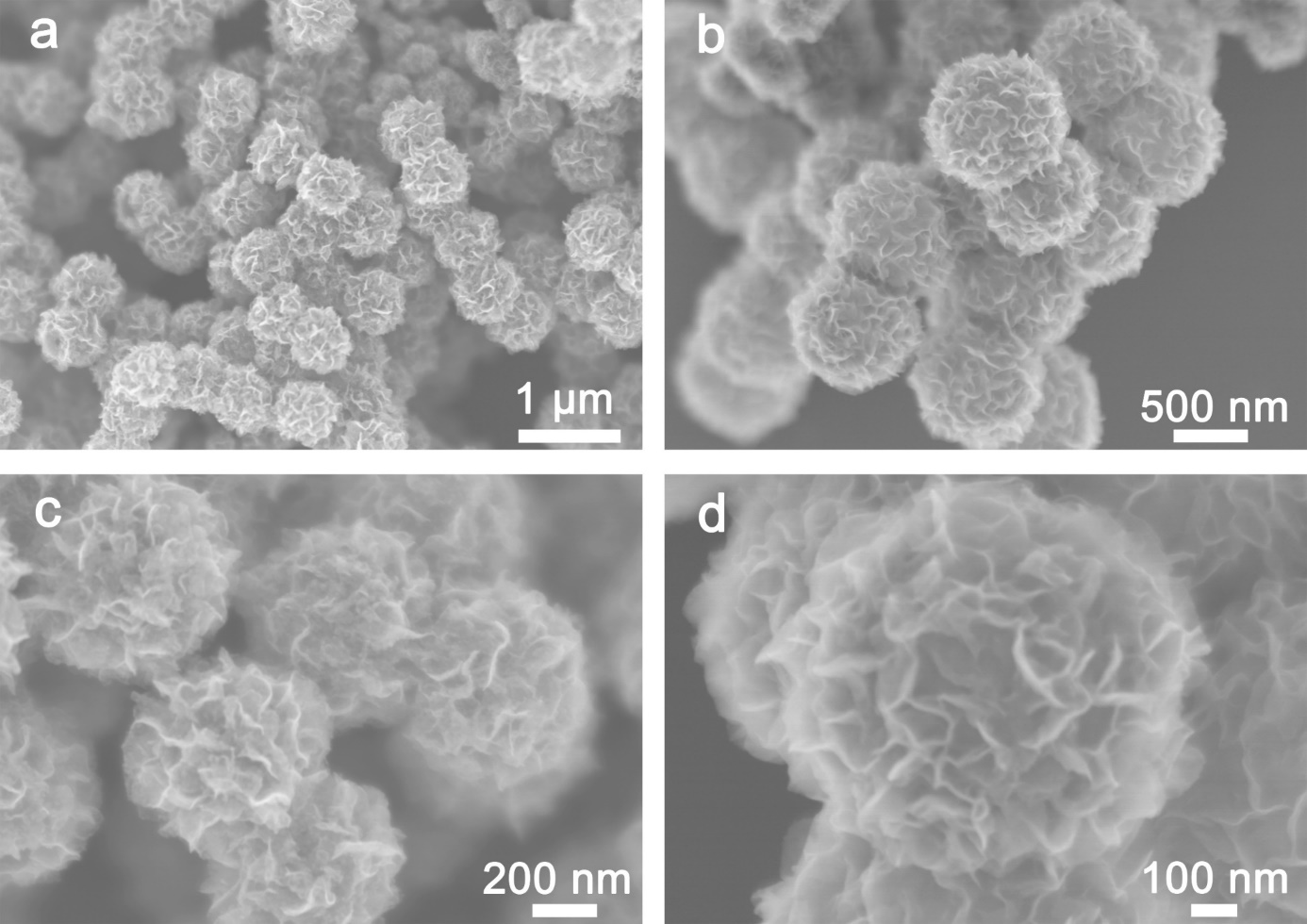


**Figure S2.** (a-d) The SEM images of the 2H-MoS_2_-Sn_1_ at different magnifications. Sn single atoms are anchored on the 2H-MoS_2_ surface without forming particles.


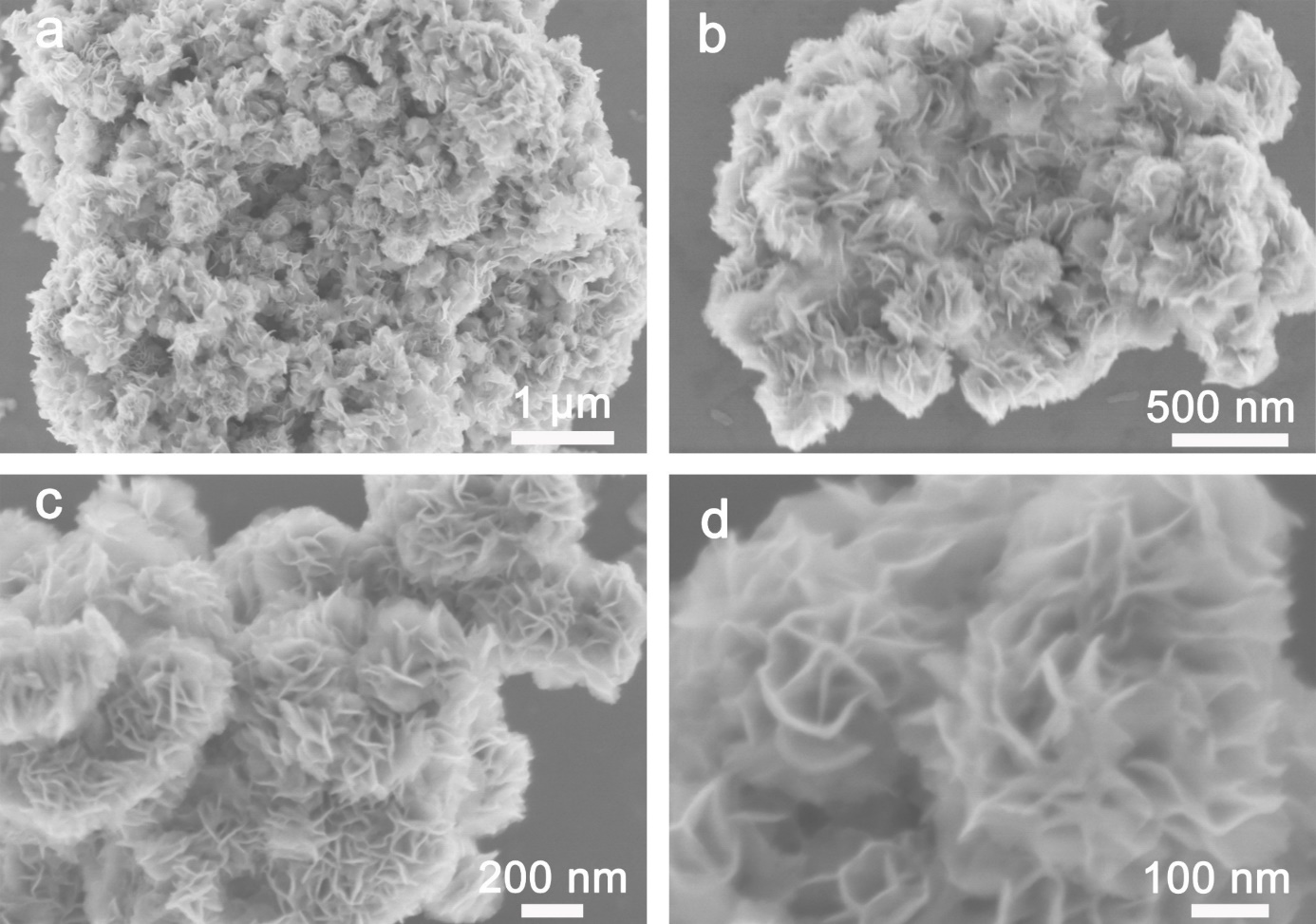


**Figure S3.** (a-d) The SEM images of the 1T-MoS_2_ at different magnifications. 1T-MoS_2_ appears as a flower-shaped sphere with a size of 300 nm, composed of ultra-thin nanosheets.


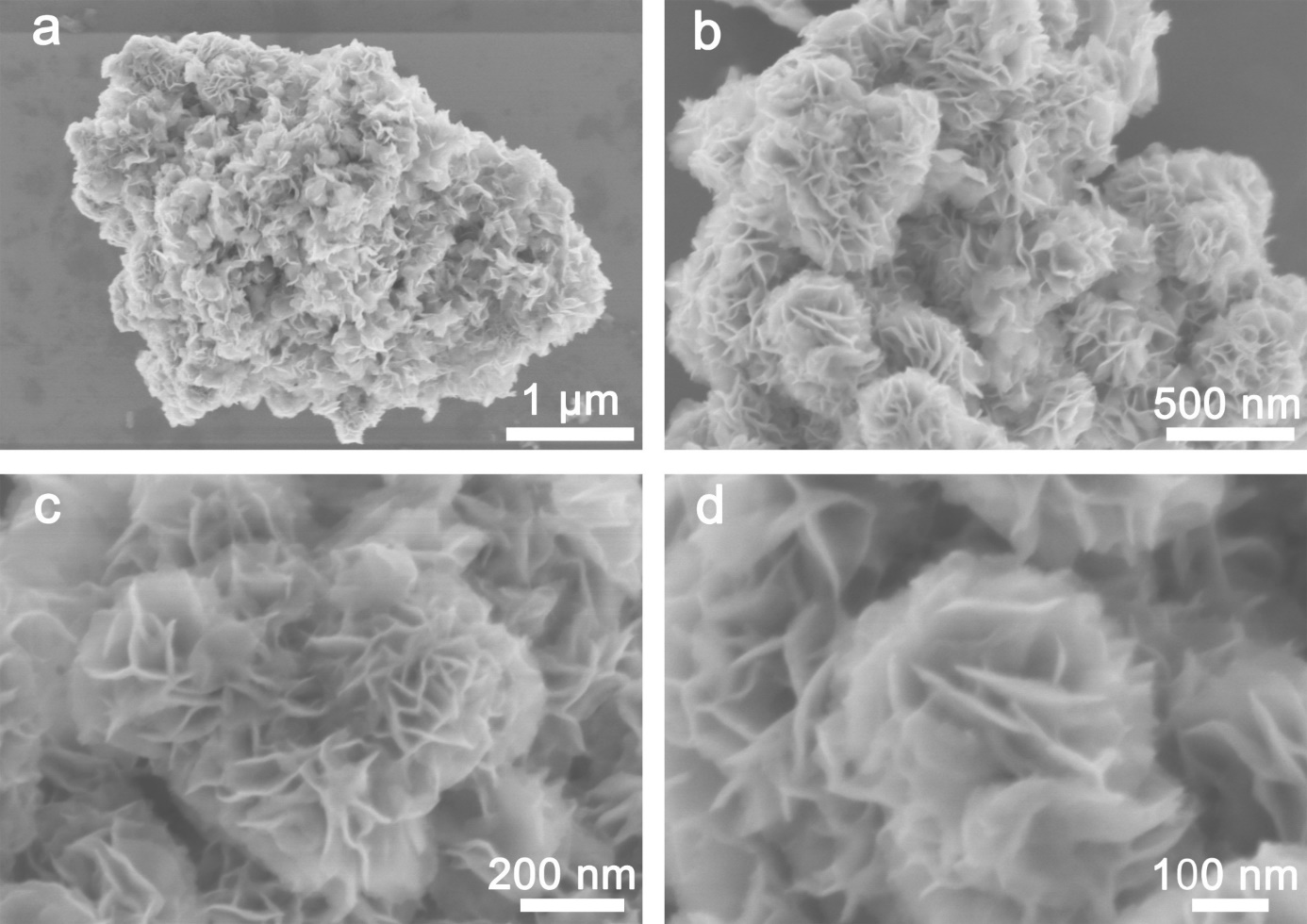


**Figure S4.** (a-d) The SEM images of the 1T-MoS_2_-Sn_1_ at different magnifications. Sn single atoms are anchored on the surface of 1T-MoS_2_ without altering their original morphology.


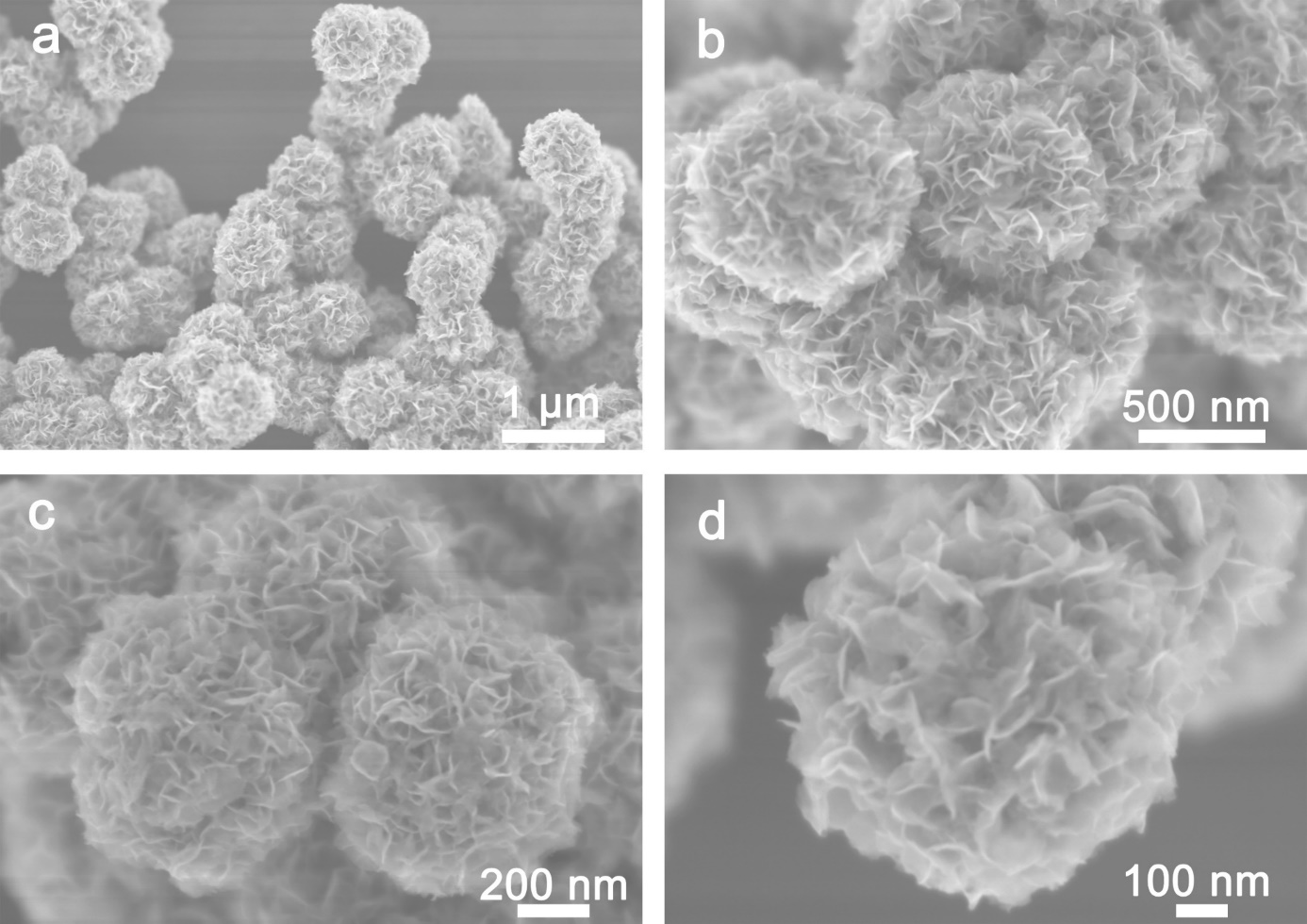


**Figure S5.** (a-d) The SEM images of the 2H@1T-MoS_2_ at different magnifications. The growth of 1T-MoS_2_ on the surface of 2H-MoS_2_ is forming rough nanospheres.


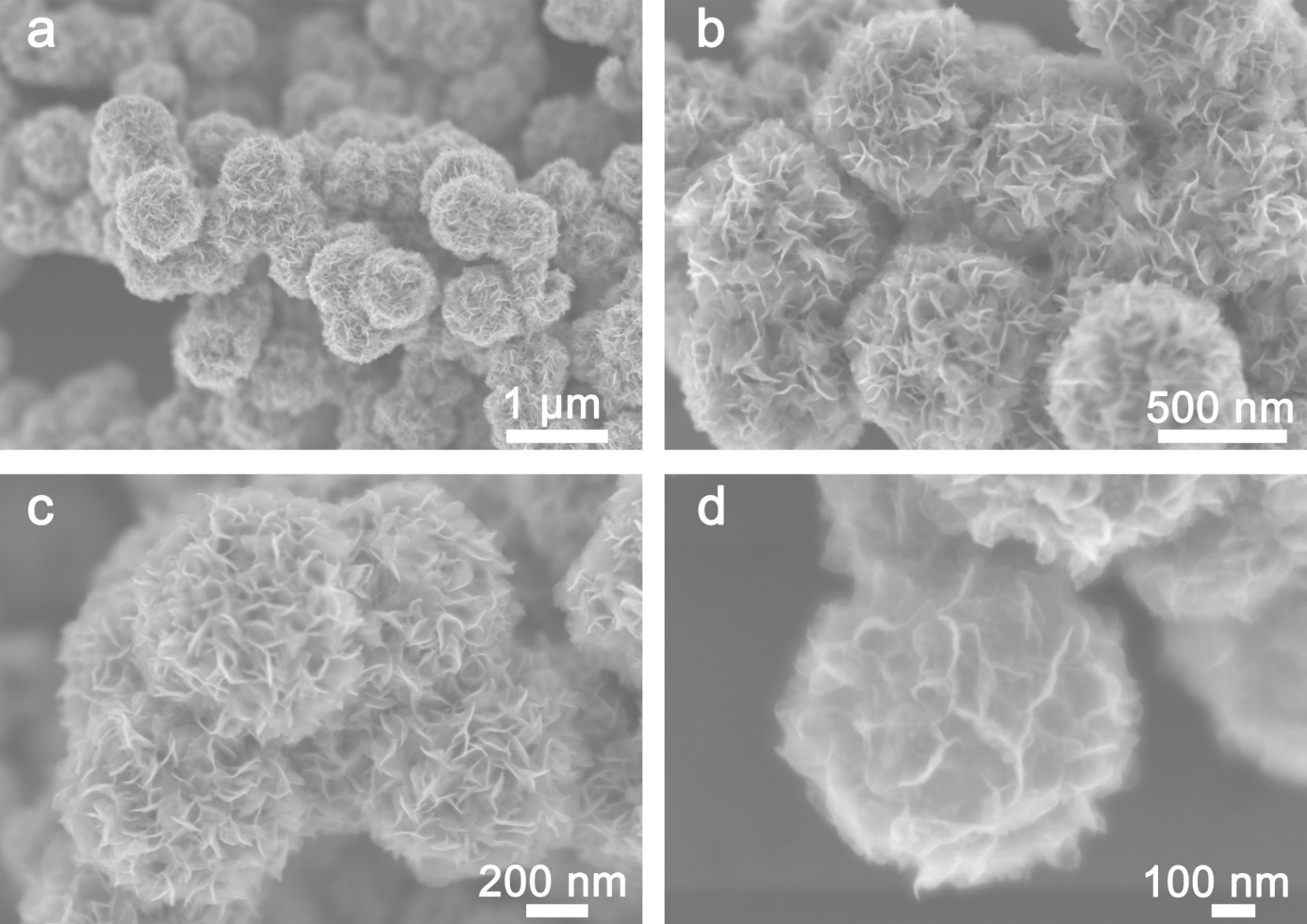


**Figure S6.** (a-d) The SEM images of the 2H@1T-MoS_2_-Sn_1_ nanoreactor at different magnifications. It is observed that the morphology of 2H@1T-MoS_2_ with Sn remains consistent with that of 2H@1T-MoS_2_. This suggests that the introduction of Sn without altering the morphology of 2H@1T-MoS_2_.


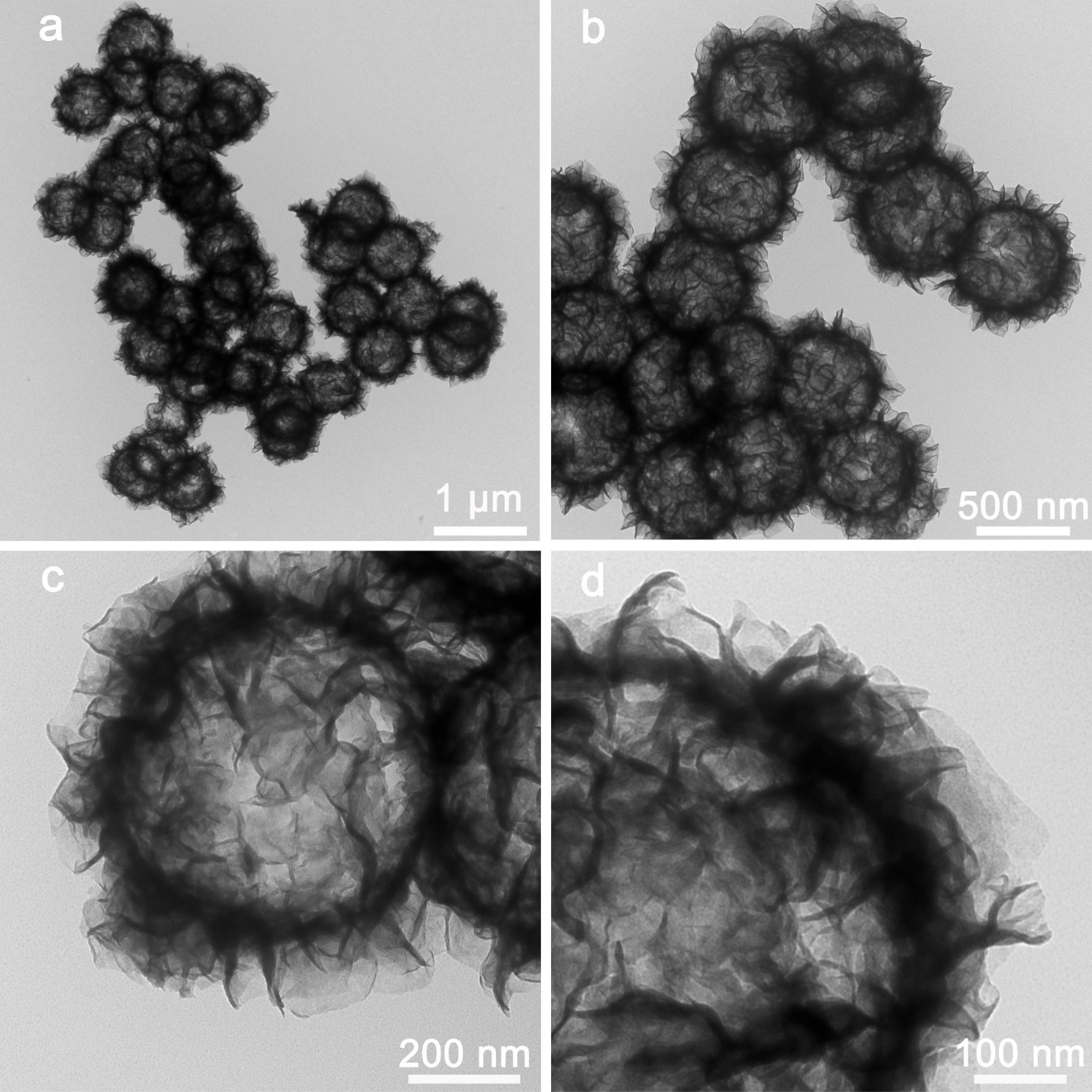


**Figure S7.** (a-d) Transmission electron microscopy (TEM) images of the 2H-MoS_2_ at different magnifications. 2H-MoS_2_ displays hollow nano flower balls with a shell size of 50 nm.


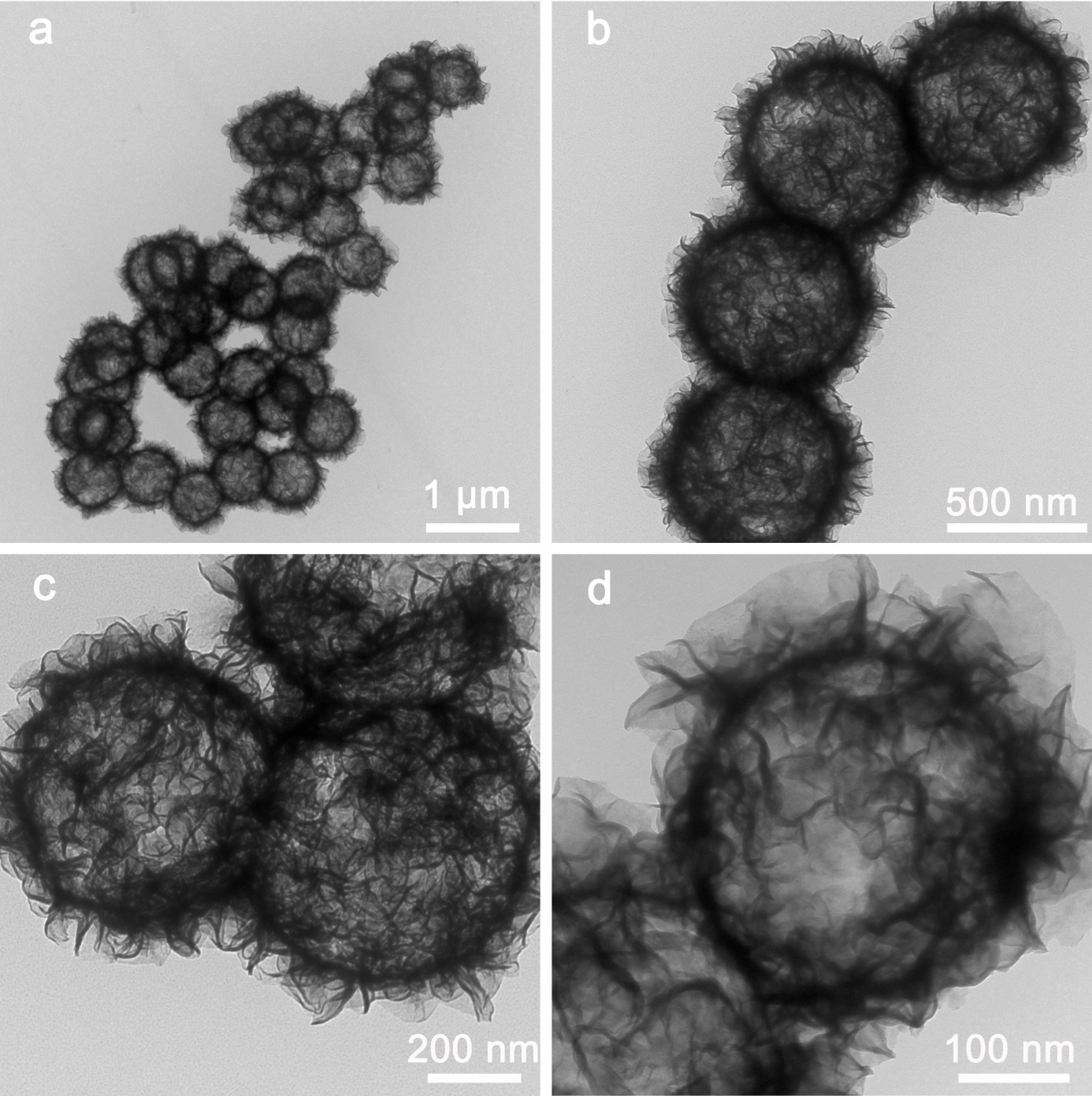


**Figure S8.** (a-d) The TEM images of the 2H-MoS_2_-Sn_1_ at different magnifications. The morphology of 2H-MoS_2_-Sn_1_ appears to be unchanged compared to that of 2H-MoS_2_, suggesting that the addition of Sn without affecting the morphology of 2H-MoS_2_.


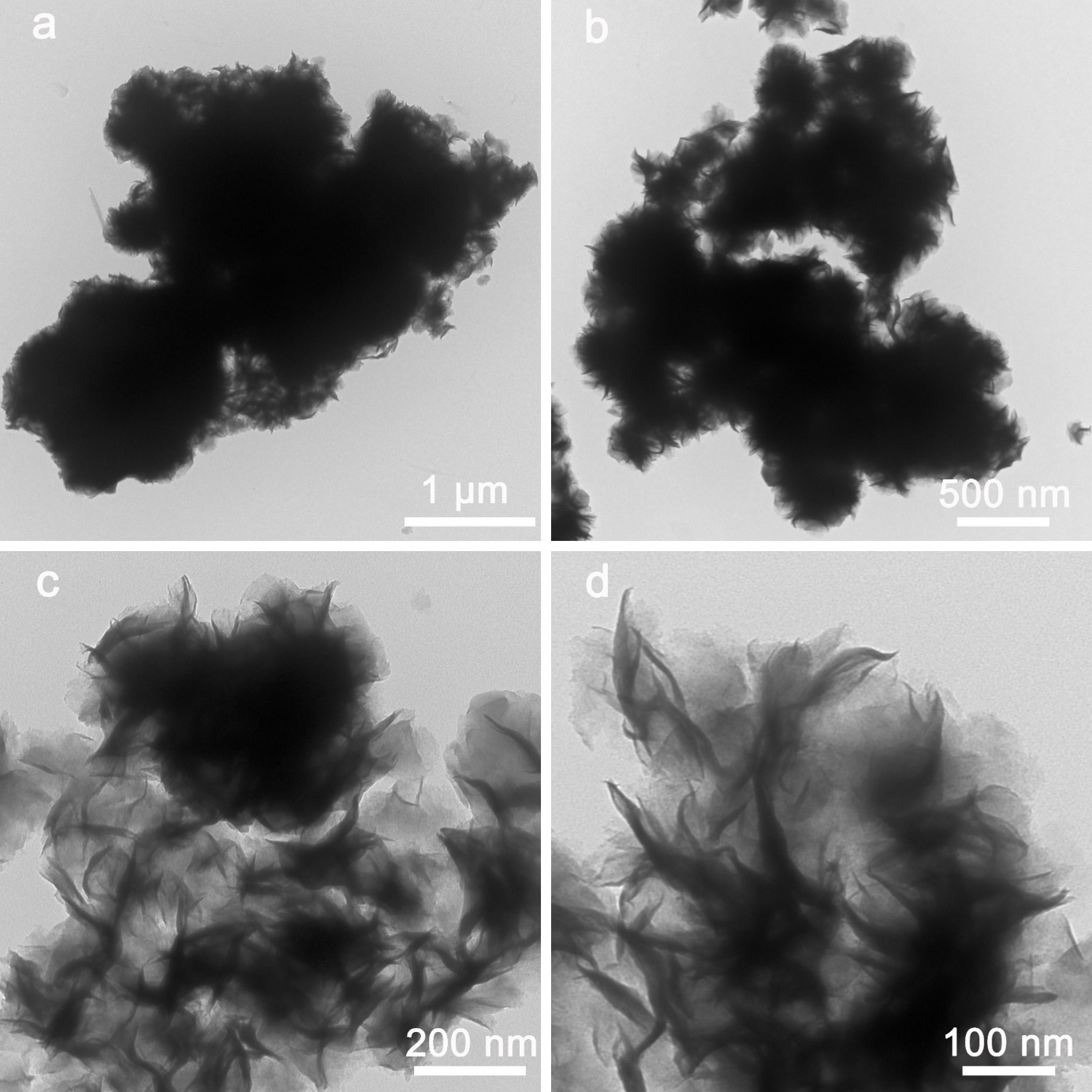


**Figure S9.** (a-d) The TEM images of the 1T-MoS_2_ at different magnifications. 1T-MoS_2_ takes the form of a flower-shaped sphere with a size of 300 nm and is composed of ultra-thin nanosheets.


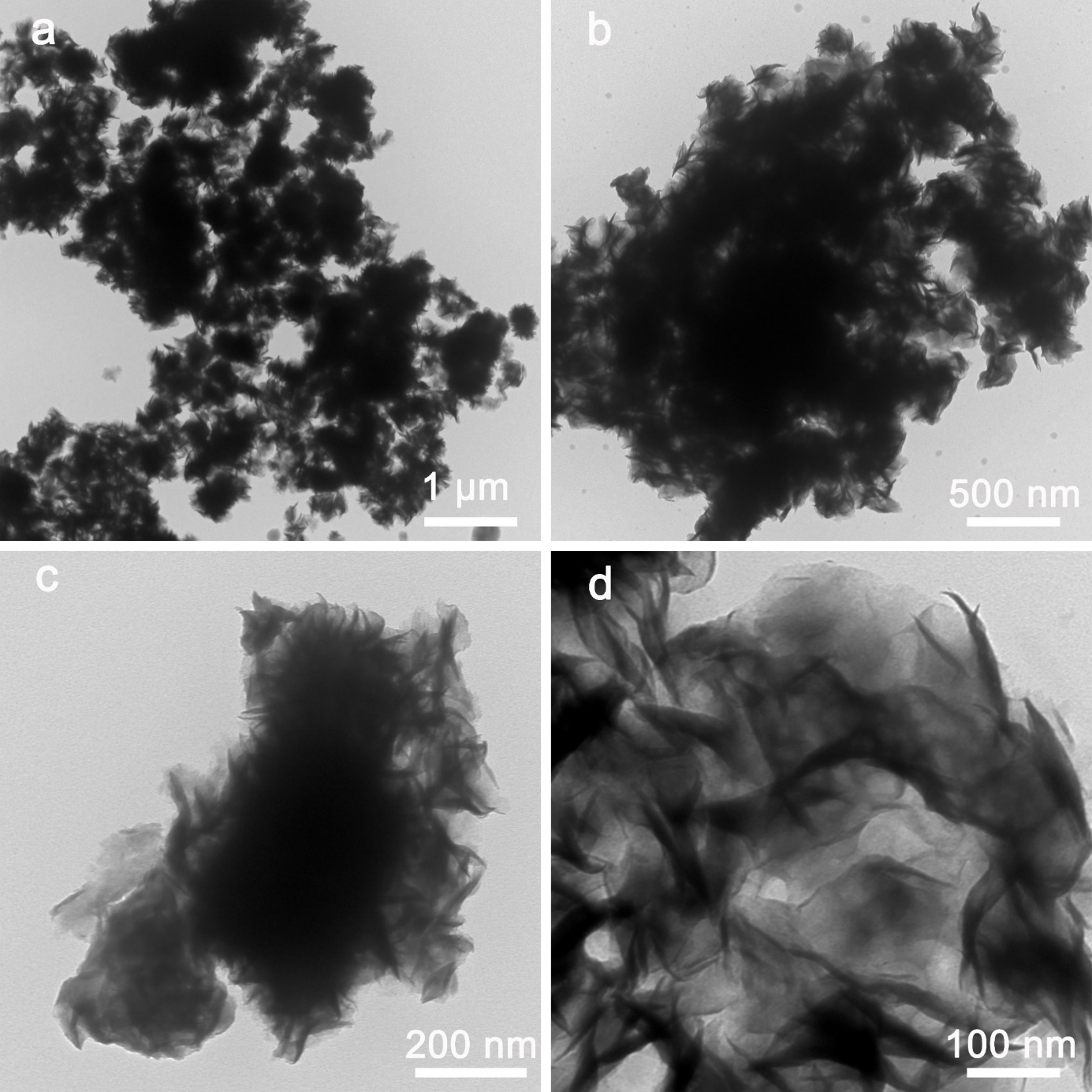


**Figure S10.** (a-d) The TEM images of the 1T-MoS_2_-Sn_1_ at different magnifications. Sn single atoms are anchored on the surface of 1T-MoS_2_ without causing any change to its original morphology.


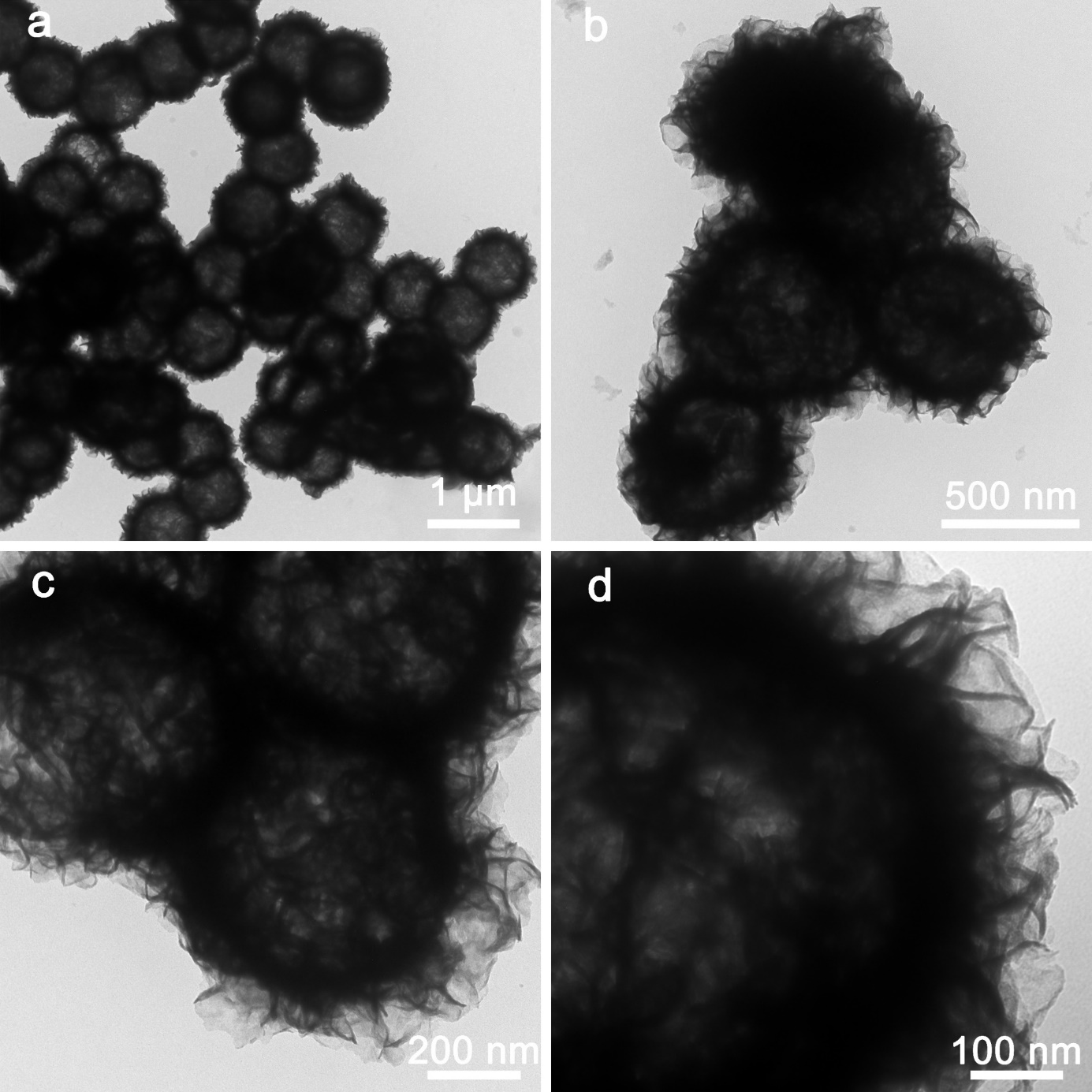


**Figure S11.** (a-d) The TEM images of the 2H@1T-MoS_2_ at different magnifications. 2H@1T-MoS_2_ exhibits a morphology of hollow flower-shaped structure, while 1T-MoS_2_ is observed to grow on the surface of 2H-MoS_2_ with a thickness of 100 nm.


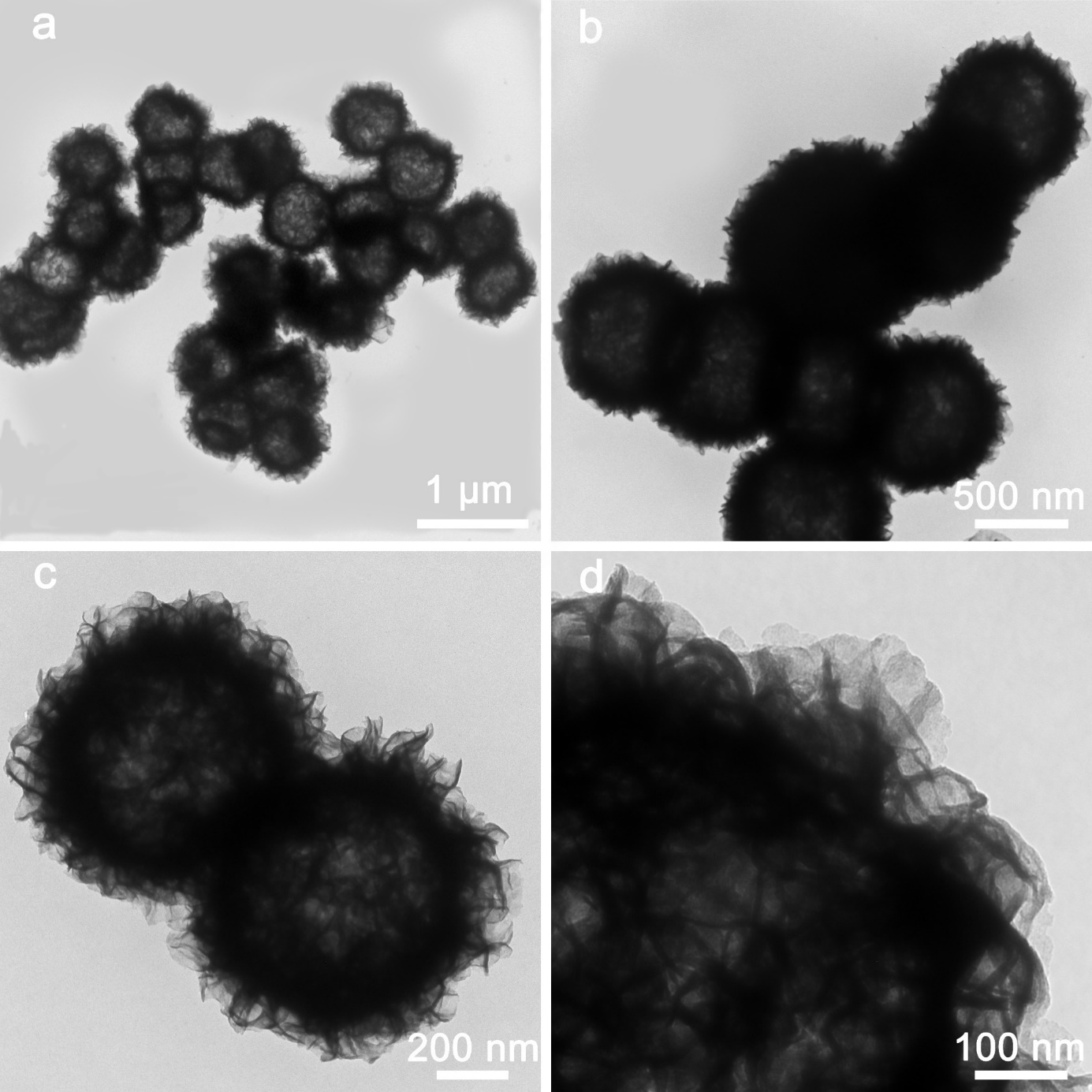


**Figure S12.** (a-d) The TEM images of the 2H@1T-MoS_2_-Sn_1_ nanoreactor at different magnifications. The morphology of 2H@1T-MoS_2_ when combined with Sn single atoms remain unchanged compared to that of 2H@1T-MoS_2_.


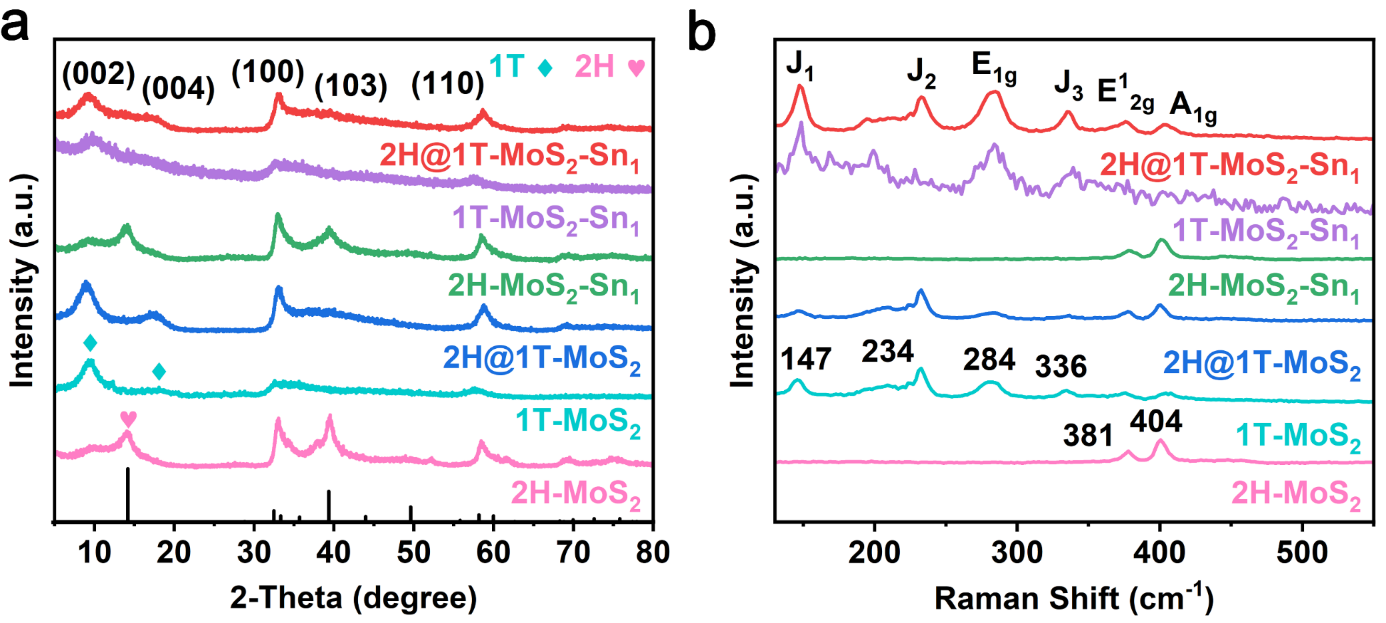


**Figure S13****.** (a) XRD patterns and (b) Raman spectra of the 2H-MoS_2_, 1T-MoS_2_, 2H@1T-MoS_2_, 2H-MoS_2_-Sn_1_, 1T-MoS_2_-Sn_1_ and 2H@1T-MoS_2_-Sn_1_.

Through XRD and Raman spectroscopy analysis, no additional peaks corresponding to Sn are detected, indicating that the incorporation of Sn single atoms has no impact on the inherent structure.


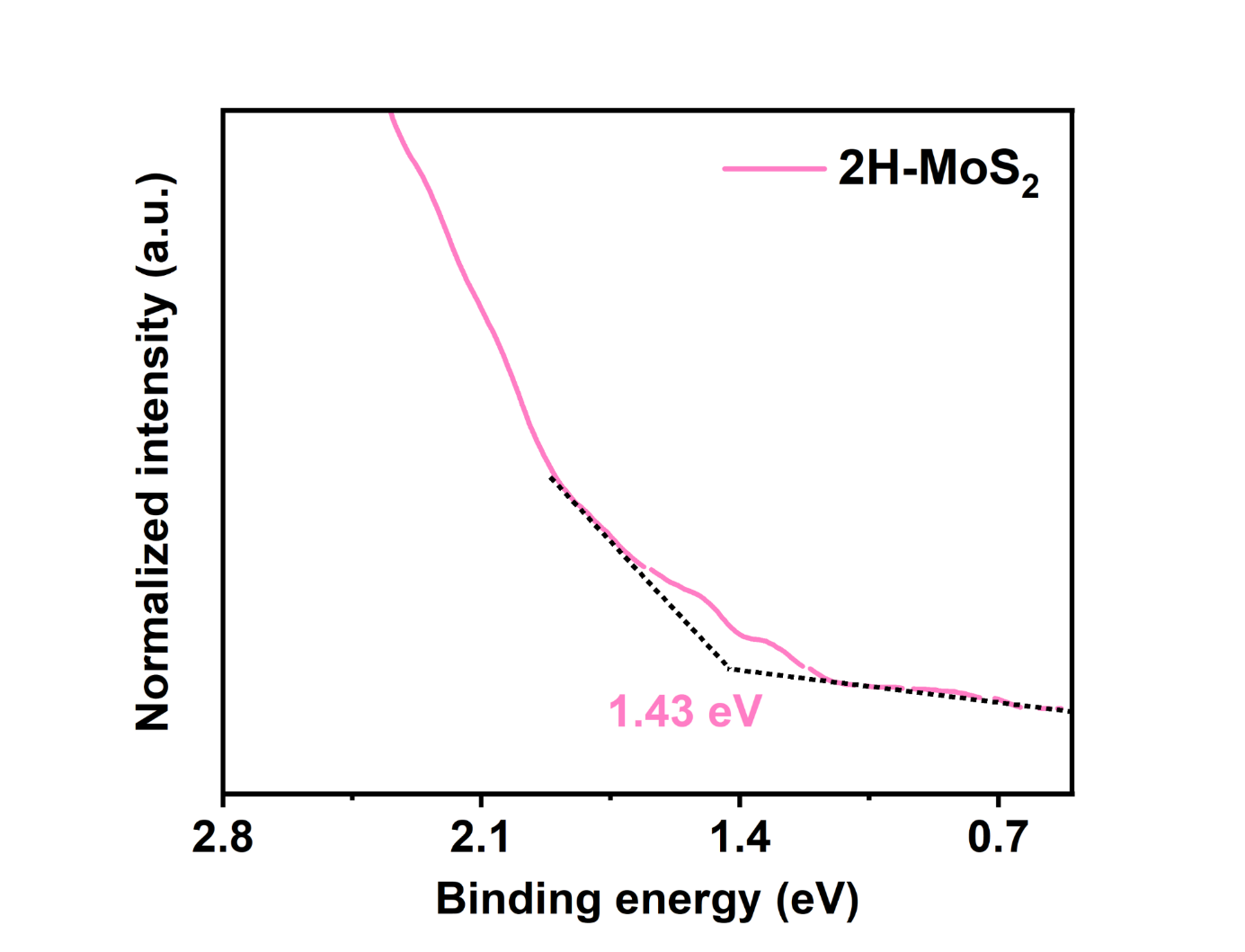


**Figure S14.** Valence band (VB) structure of 2H-MoS_2_.

UPS measures the energy distribution of valence shell electrons to provide insights into the valence electron structure, encompassing the valence band spectrum, work function, and state density distribution of materials.


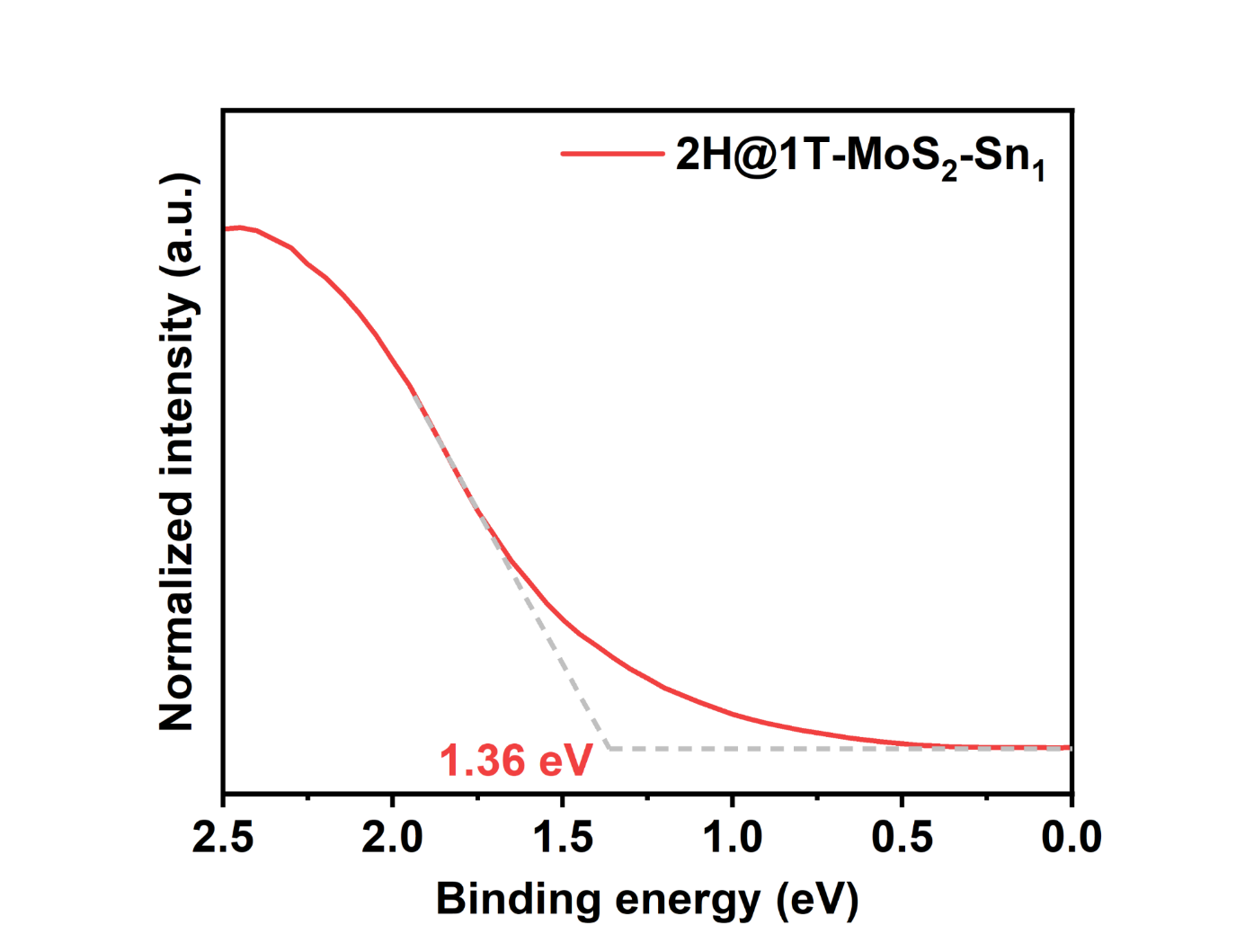


**Figure S15.** The VB of 2H@1T-MoS_2_-Sn_1_ is determined to be 1.36 eV based on ultra-sensitive surface-based UPS.


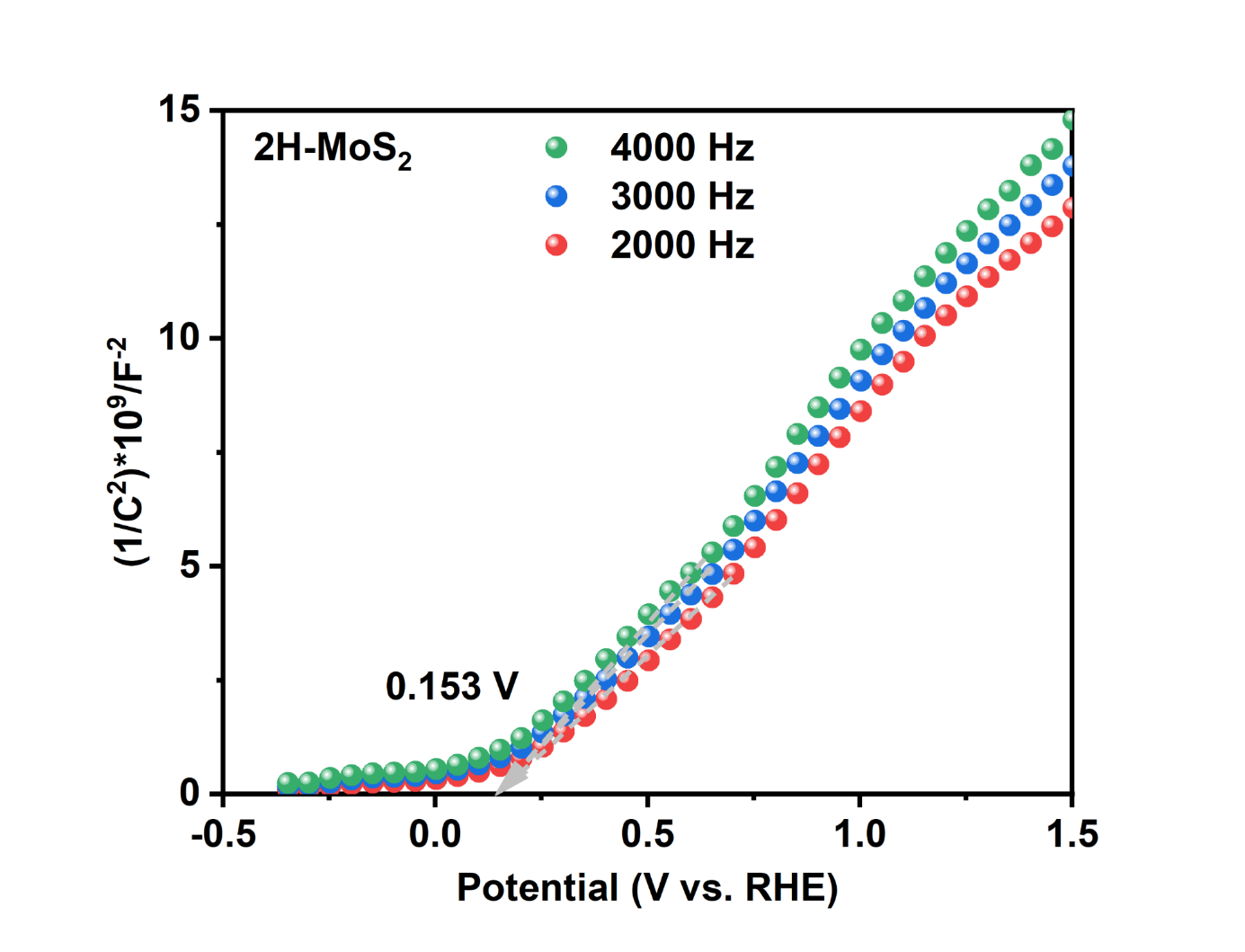


**Figure S16.** Mott-Schottky plots of 2H-MoS_2_.

As shown in the figure, 2H-MoS_2_ exhibits n-type semiconductor characteristics at frequencies of 2000 Hz, 3000 Hz, and 4000 Hz, with a flat band potential of 0.153 V.


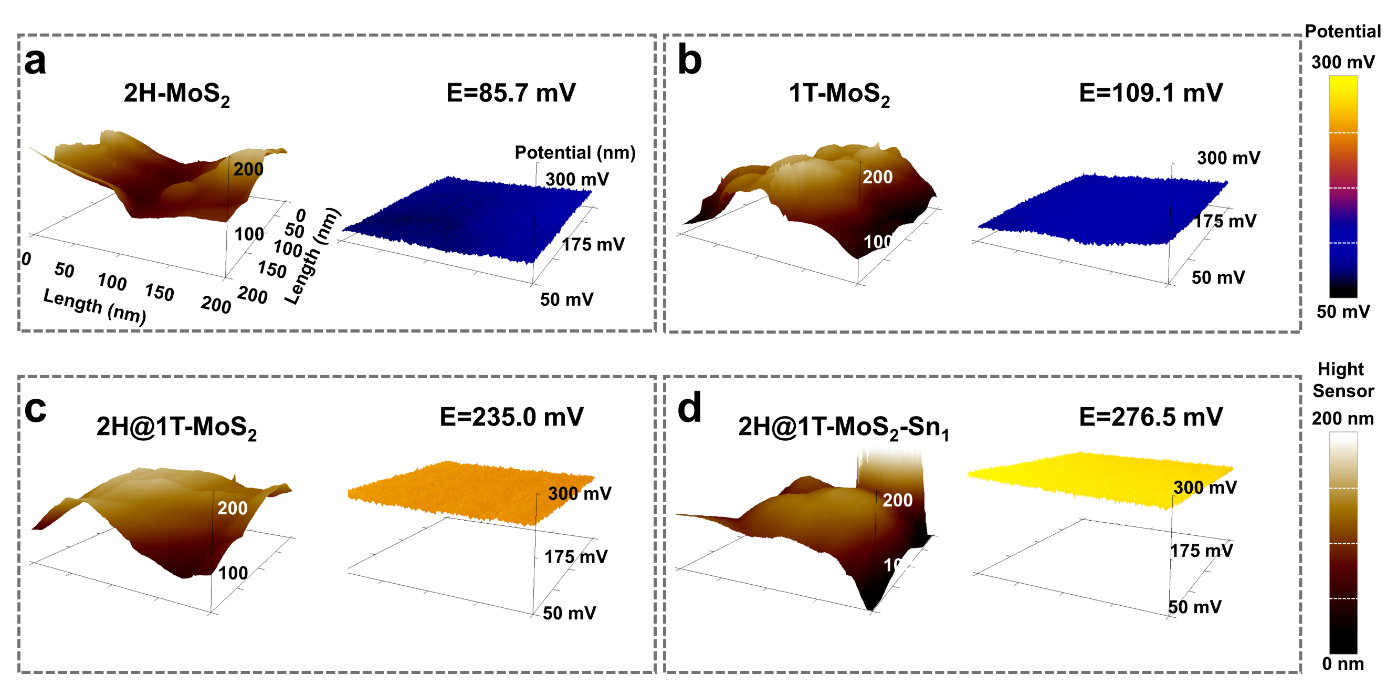


**Figure S17.** Surface potential measured by KPFM for (a) 2H-MoS_2_, (b) 1T-MoS_2_, (c) 2H@1T-MoS_2_, (d) 2H@1T-MoS_2_-Sn_1_.

The KPFM analysis discloses that 2H@1T-MoS_2_-Sn_1_ attains a maximum surface potential of 276.5 mV. This value substantially surpasses those of its counterparts, namely 2H@1T-MoS_2_ (235.0 mV), 1T-MoS_2_ (109.1 mV), and 2H-MoS_2_ (85.7 mV).


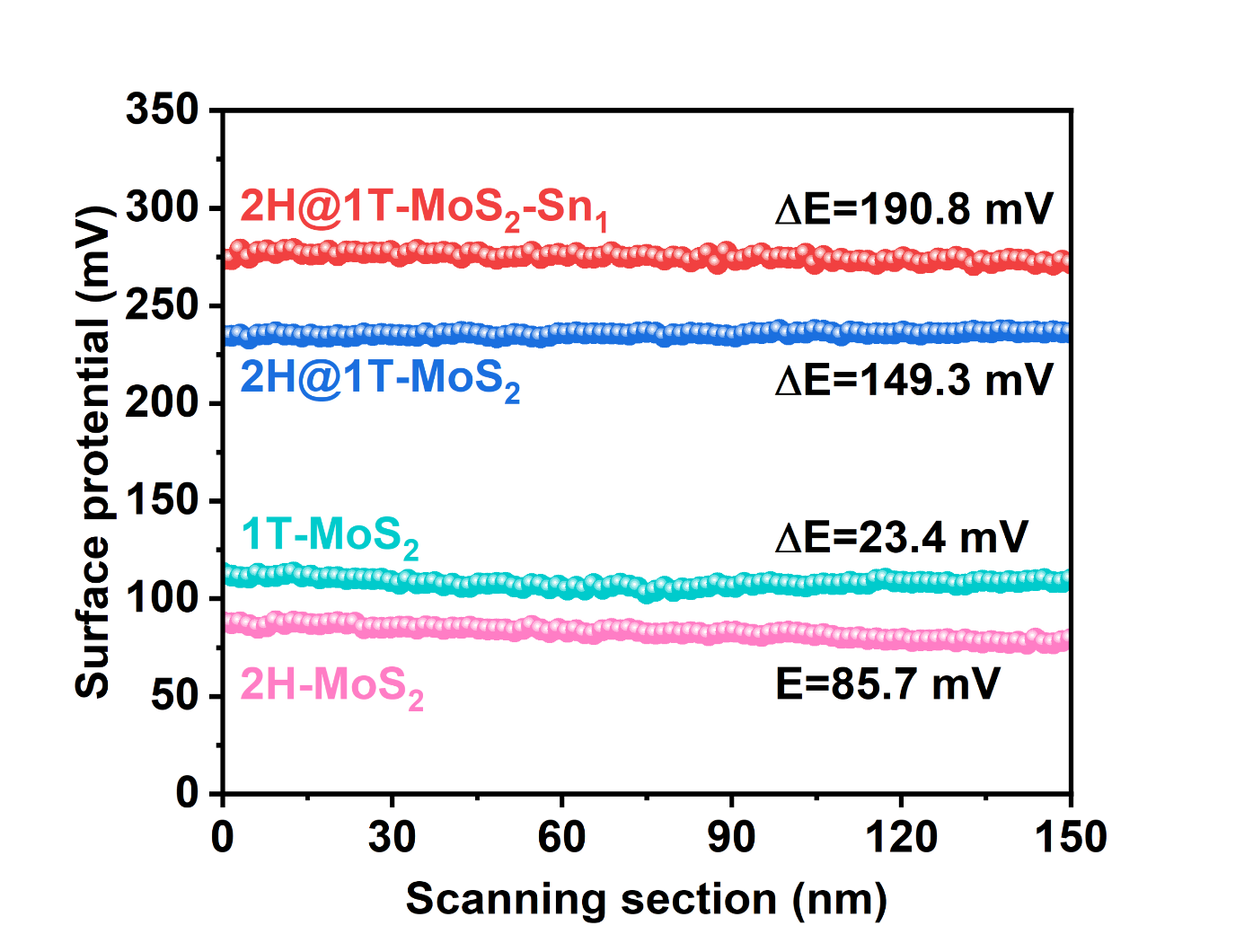


**Figure S18.** Surface contact potential differences of 2H-MoS_2_, 1T-MoS_2_, 2H@1T-MoS_2_ and 2H@1T-MoS_2_-Sn_1_ are measured by KPFM.

The results show that the surface potential of 2H@1T-MoS_2_-Sn_1_ is 190.8 mV higher than that of 2H-MoS_2_.


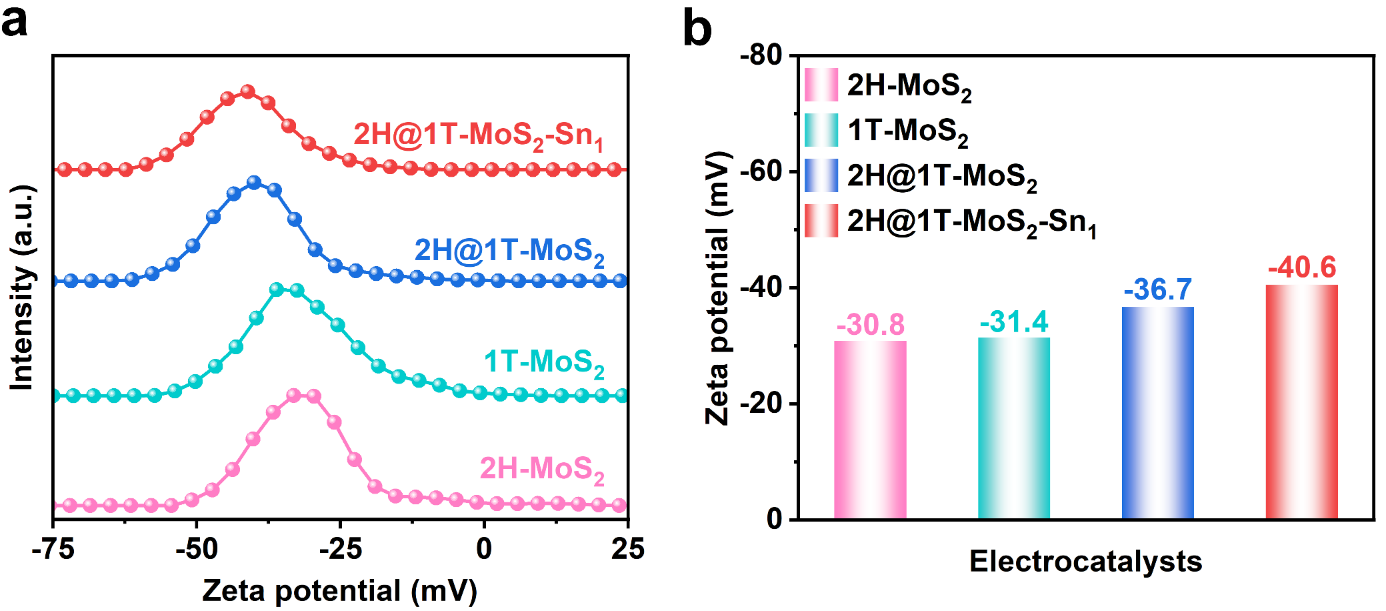


**Figure S19.** (a) The Zeta potential curves, (b) Zeta potential values of 2H-MoS_2_, 1T-MoS_2_, 2H@1T-MoS_2_ and 2H@1T-MoS_2_-Sn_1_ under neutral condition.


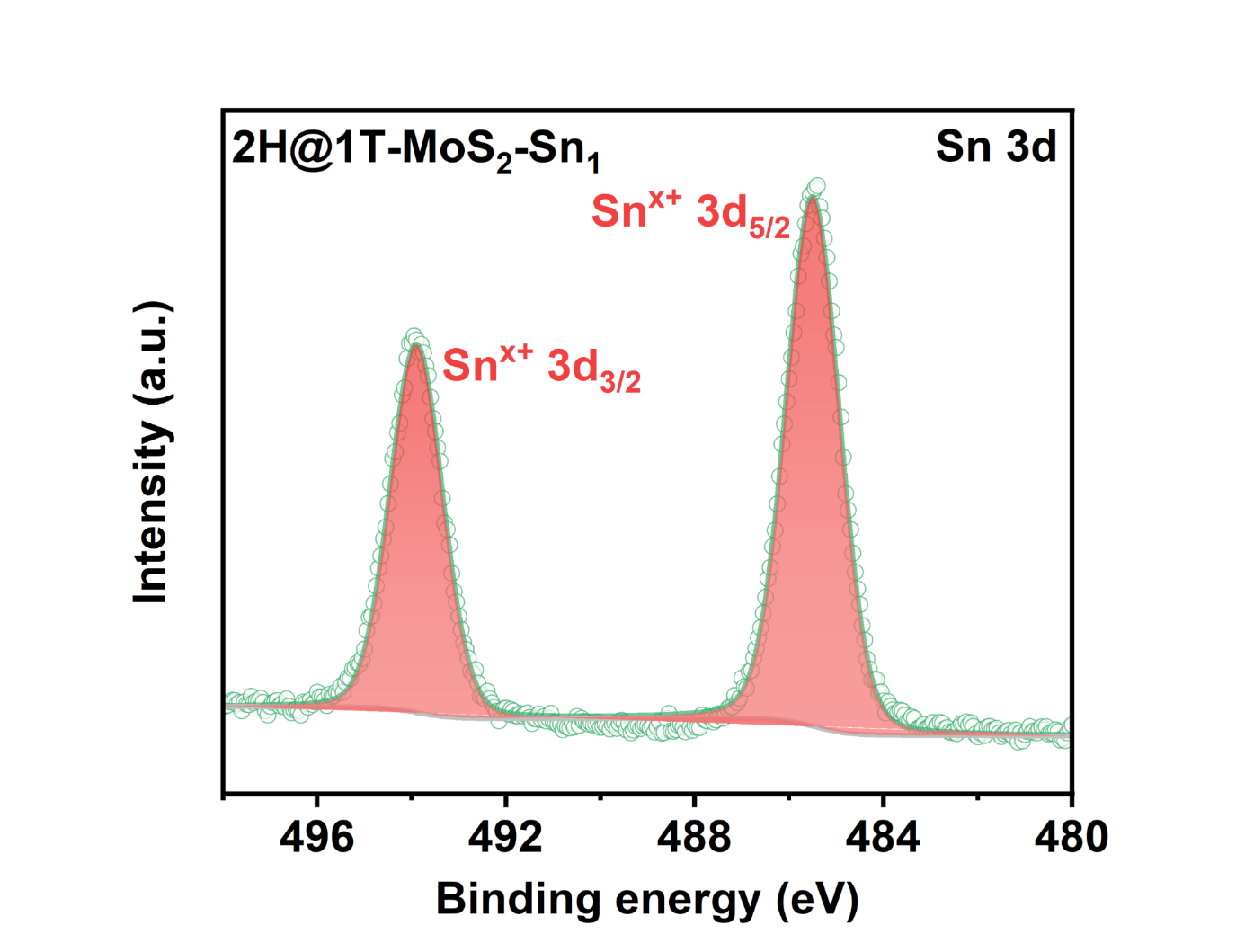


**Figure S20.** Sn 3d XPS of 2H@1T-MoS_2_-Sn_1_.

The results show that the binding energy of Sn is between Sn^0^ and Sn^2+^, indicating that the valence state of Sn is between 0 and +2.


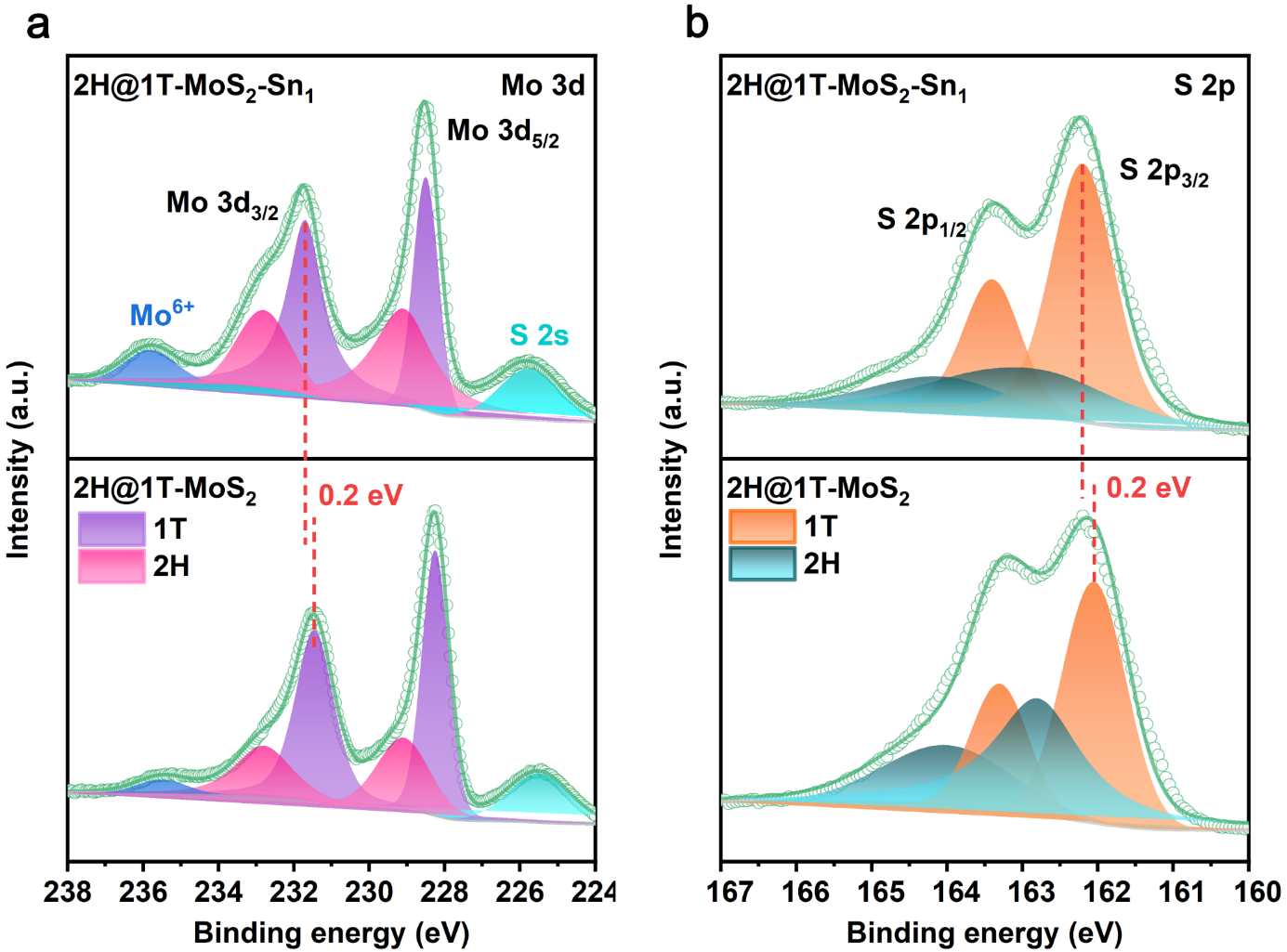


**Figure S21.** (a) Mo 3d XPS and (b) S 2p XPS of 2H@1T-MoS_2_, and 2H@1T-MoS_2_-Sn_1_.

XPS of 2H@1T-MoS_2_ and 2H@1T-MoS_2_-Sn_1_, after the introduction of Sn single atom, Mo 3d peak and S 2p peak have positive shift of 0.2 eV, indicating that the binding energy of 2H@1T-MoS_2_ increases and electrons are transferred from 2H@1T-MoS_2_ to Sn.


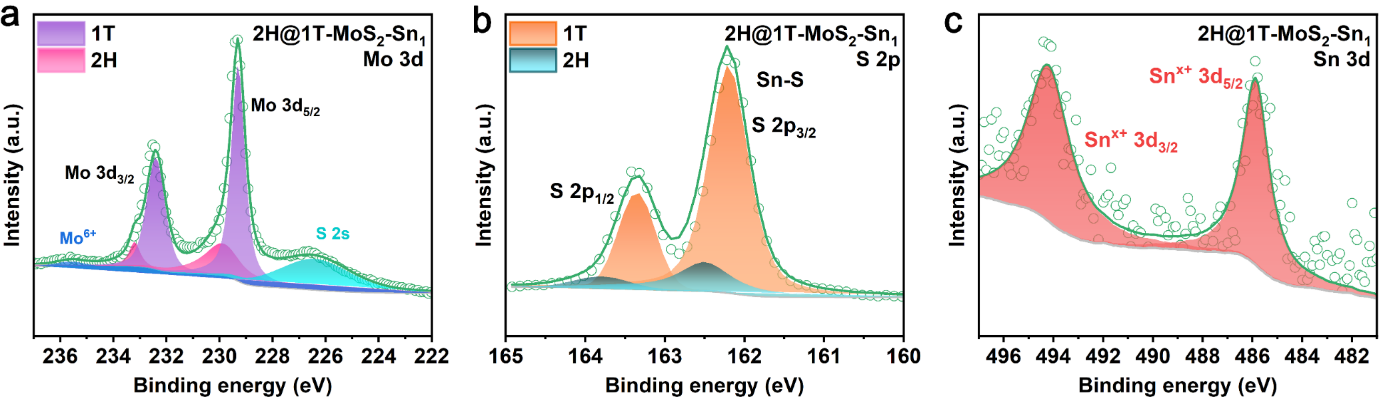


**Figure S22.** (a) Mo 3d, (b) S 2p and (c) Sn 3d synchrotron radiation photoemission spectroscopy (SRPES) of 2H@1T-MoS_2_-Sn_1_.

The SRPES of 2H@1T-MoS_2_-Sn_1_ further indicates the formation of Sn-S bonds.


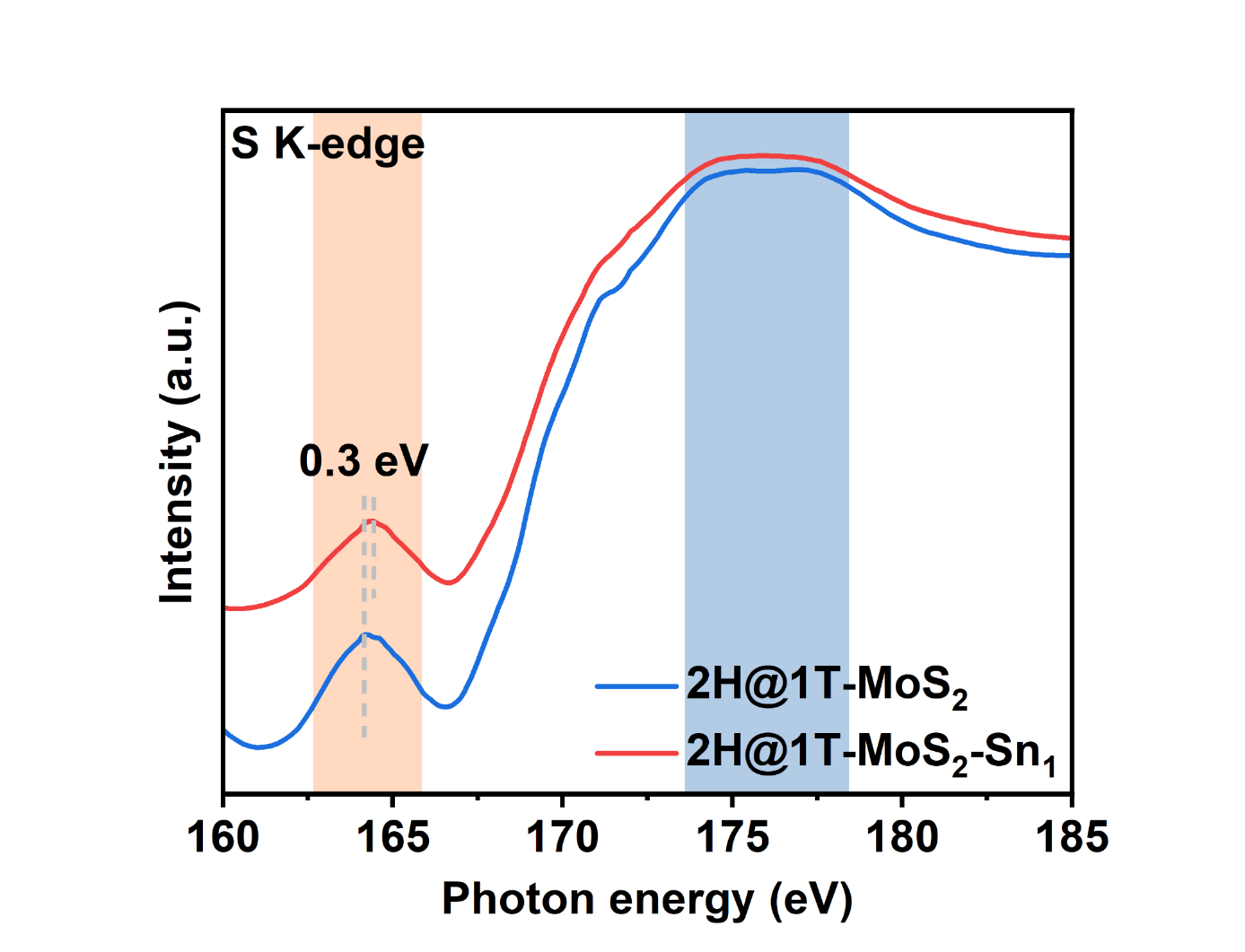


**Figure S23.** (a) Normalized K-edge XANES of S.

The results show that Sn introduction induces a +0.3 eV S K-edge shift via Sn-S bond-mediated electron transfer from S to Sn.


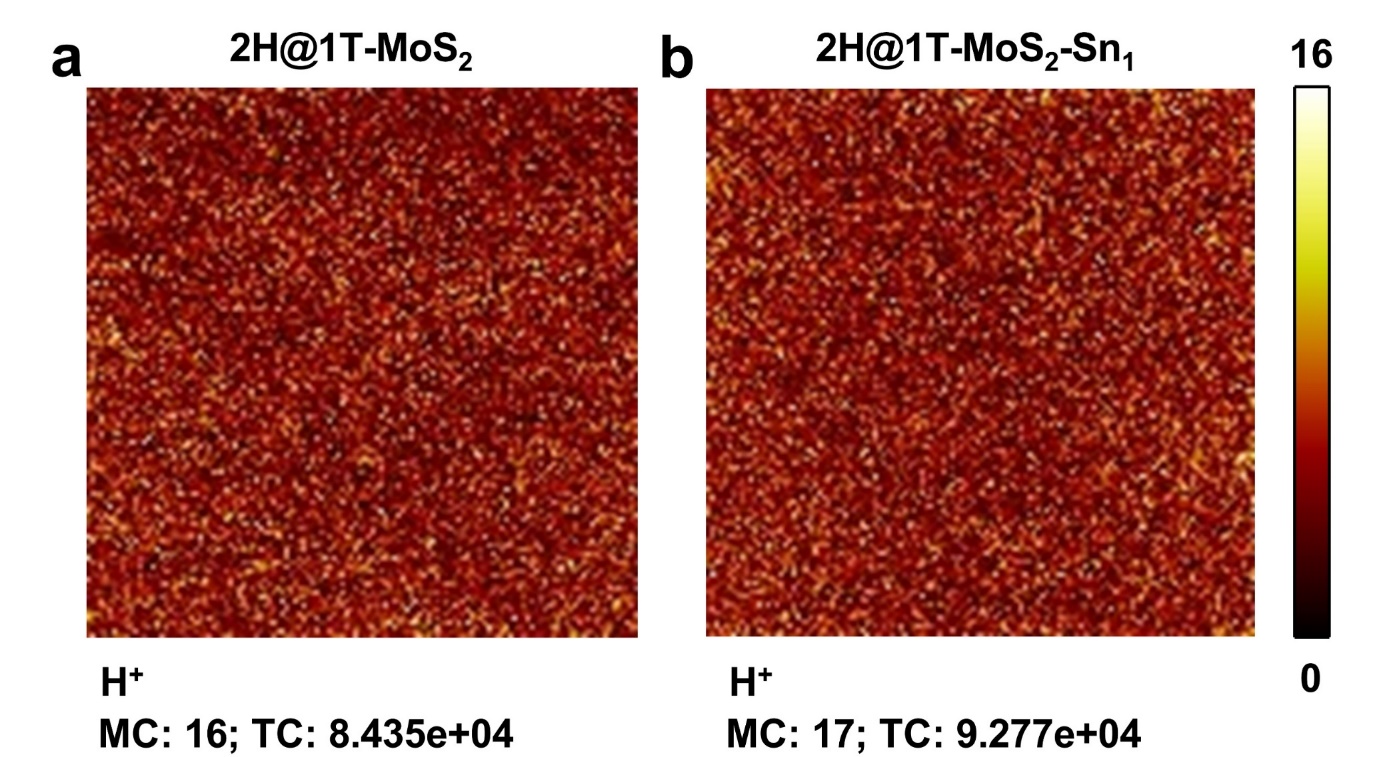


**Figure S24.** Hydrogen distribution on (a) 2H@1T-MoS_2_ and (b) 2H@1T-MoS_2_-Sn_1_ analyzed by time-of-flight secondary ion mass spectrometry (TOF-SIMS).


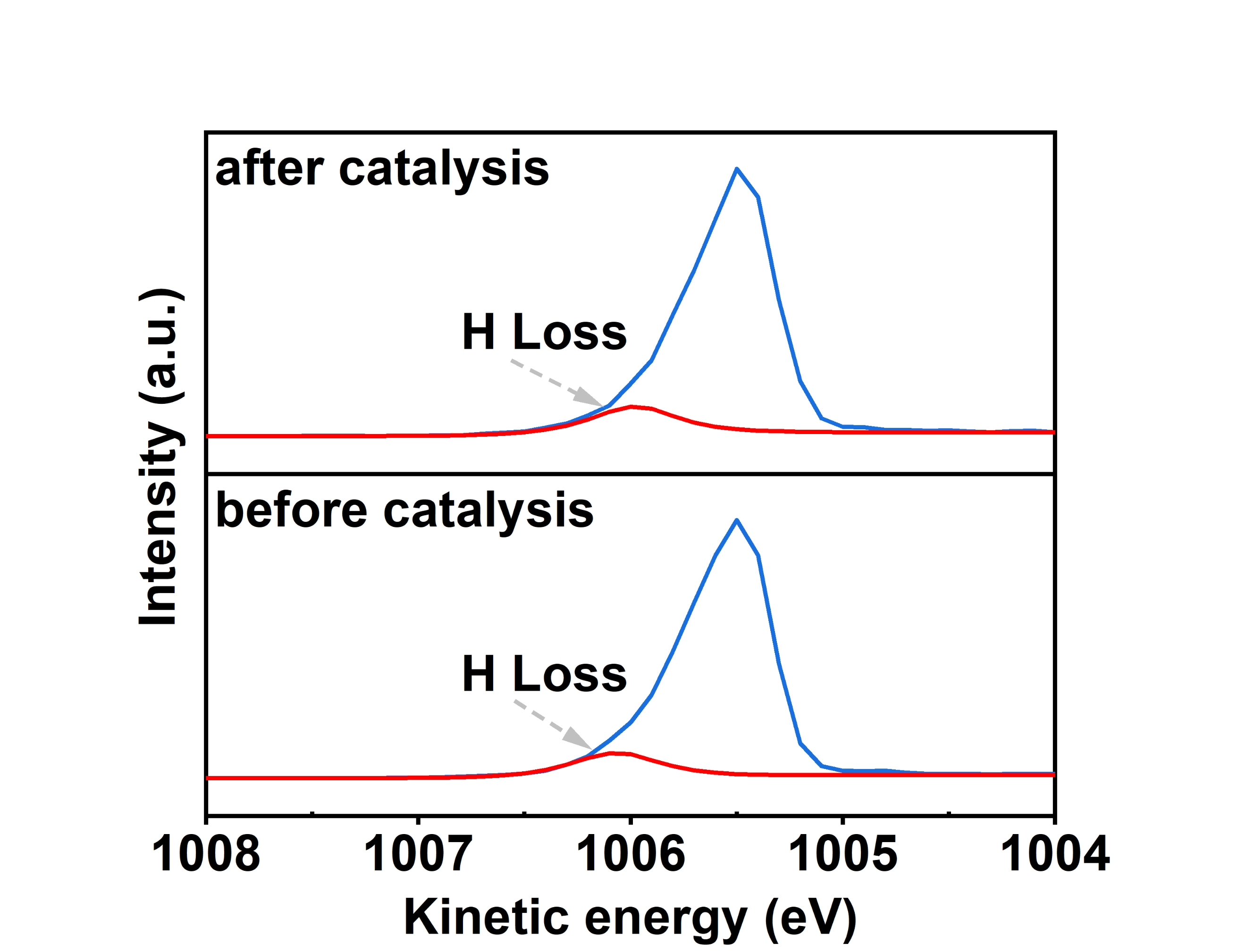


**Figure S25.** Hydrogen content in 2H@1T-MoS_2_-Sn_1_ nanoreactor before and after reaction analyzed by reflection electron energy loss spectroscopy (REELS).

The experimental data indicate that the hydrogen content remains stable at approximately 10% in the 2H@1T-MoS_2_-Sn_1_ nanoreactor both before and after the reaction.


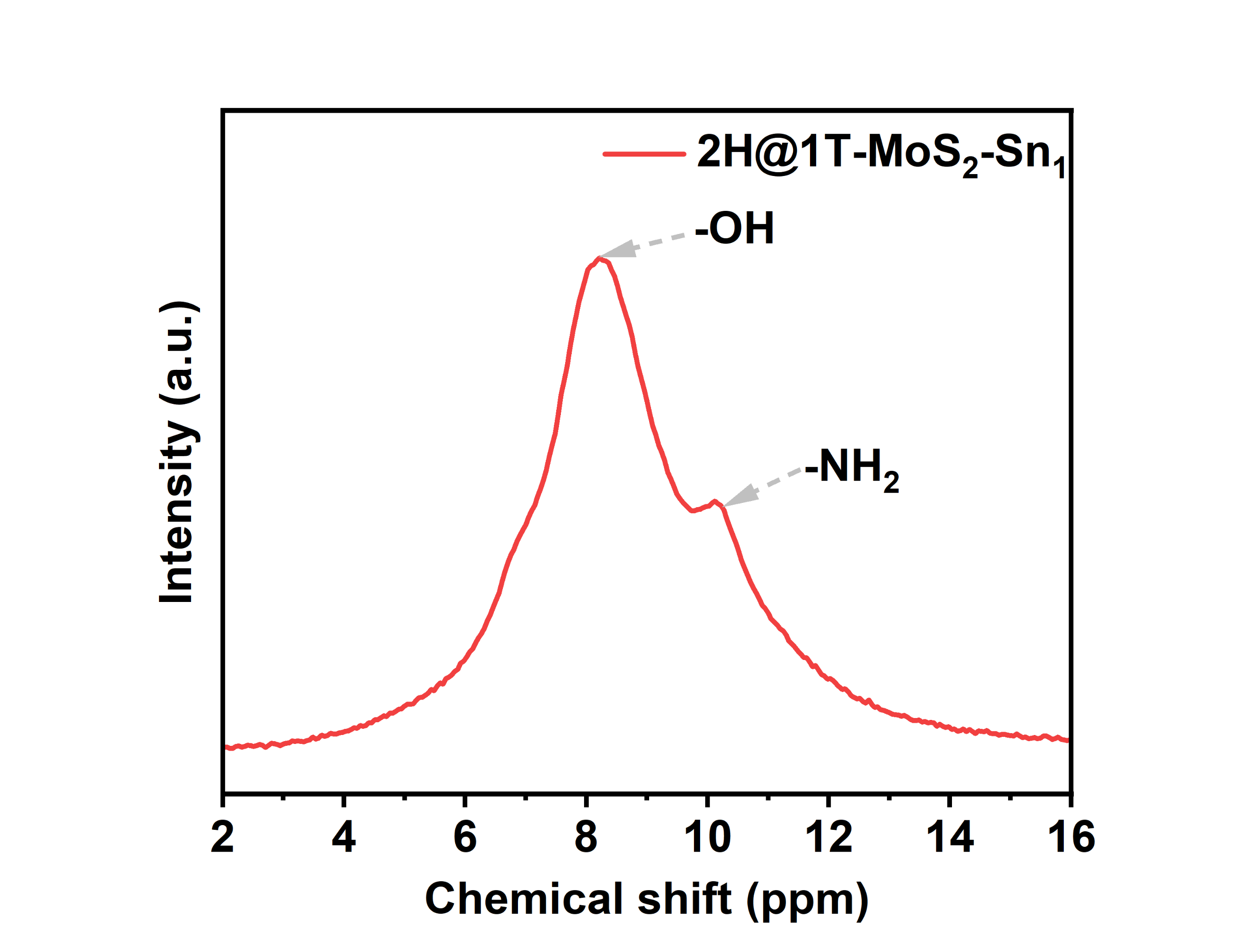


**Figure S26.** Hydrogen nuclear magnetic resonance (^1^H NMR) of 2H@1T-MoS_2_-Sn_1_. The results show that the surface adsorption peaks of hydroxyl (-OH) and residual amino (-NH_2_) are 8.8 ppm and 10.2 ppm, respectively.


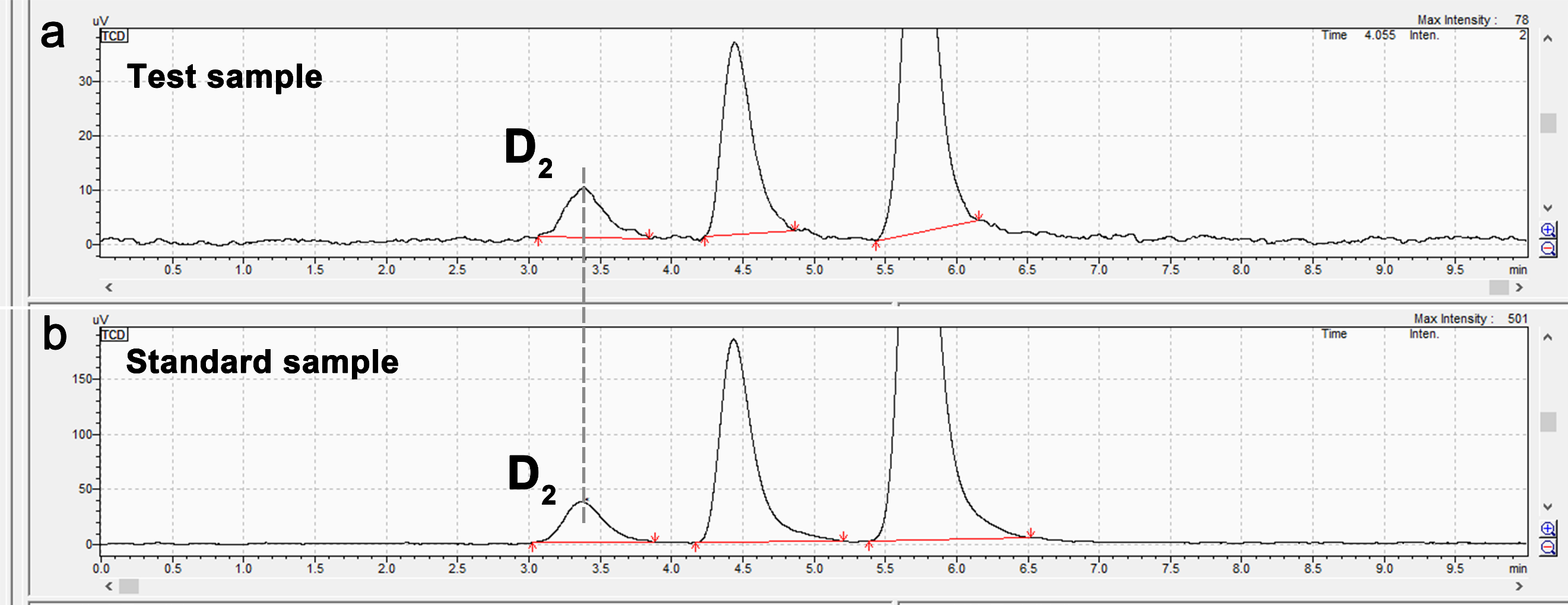


**Figure S27.** D_2_ isotopic labeling experiments for probing HER mechanisms via gas chromatography (GC). (a) Electrocatalytic D_2_ generation data under applied potential, (b) the chromatogram of D_2_ reference (calibration standard).


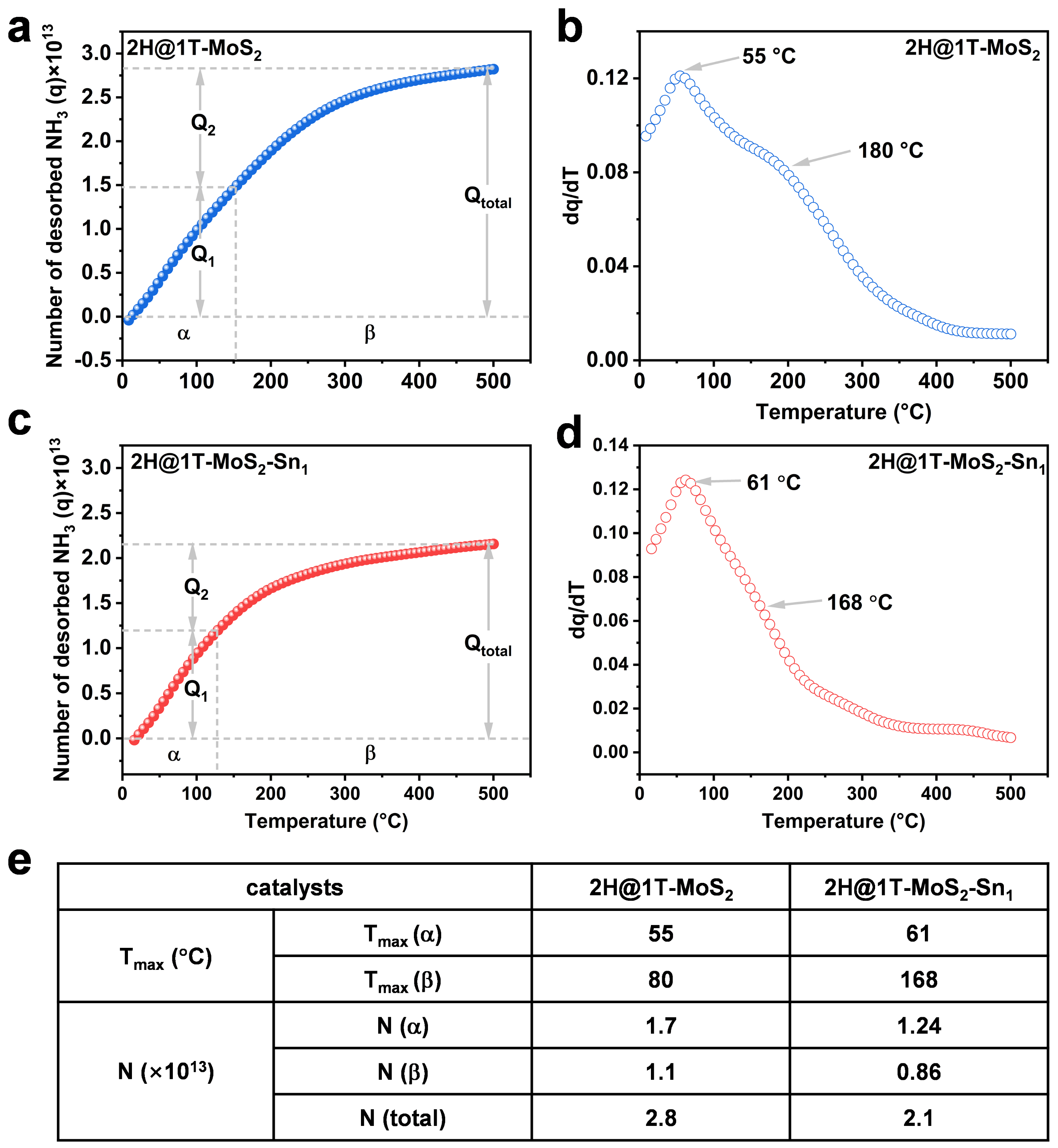


**Figure S28.** Ammonia temperature-programmed desorption (NH_3_-TPD) analysis for surface acidity of 2H@1T-MoS_2_ and 2H@1T-MoS_2_-Sn_1_. (a) NH_3_ desorption profiles of 2H@1T-MoS_2_; (b) Temperature-dependent desorption rate of 2H@1T-MoS_2_; (c) NH_3_ desorption profiles of 2H@1T-MoS_2_-Sn_1_; (d) Temperature-dependent desorption rate of 2H@1T-MoS_2_-Sn_1_; (e) Comparative analysis of NH_3_ desorption capacity and temperature between 2H@1T-MoS_2_ and 2H@1T-MoS_2_-Sn_1_.


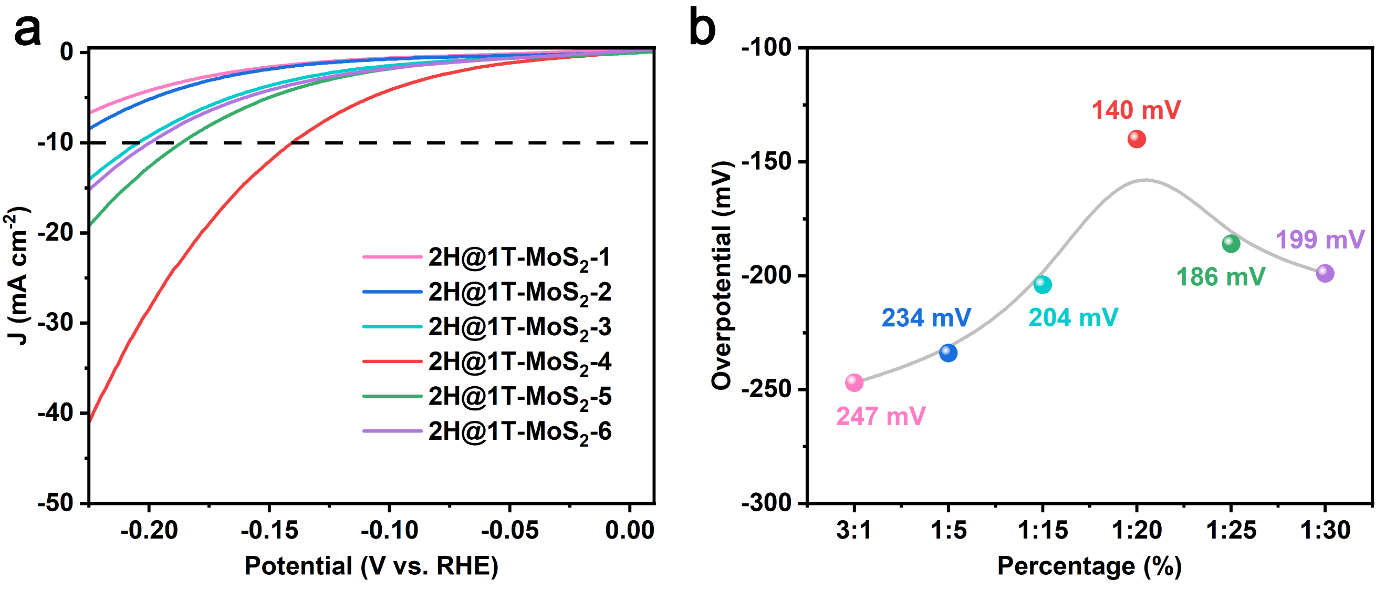


**Figure S29.** (a) The obtained LSV curves of 2H@1T-MoS_2_ are analyzed for different proportions. (b) The overpotential at a current density of 10 mA cm^-2^ is examined.

The test ratios corresponded to the 1T-MoS_2_:2H-MoS_2_ proportions of 3:1, 1:5, 1:15, 1:20, 1:25, and 1:30. The overpotential values for each ratio were as follows: 247 mV for the 3:1 ratio, 234 mV for the 1:5 ratio, 204 mV for the 1:15 ratio, 140 mV for the 1:20 ratio, 186 mV for the 1:25 ratio, and 199 mV for the 1:30 ratio. Notably, a distinct trend is observed, with the 1:20 ratio of 2H@1T-MoS_2_ demonstrating exceptional performance.


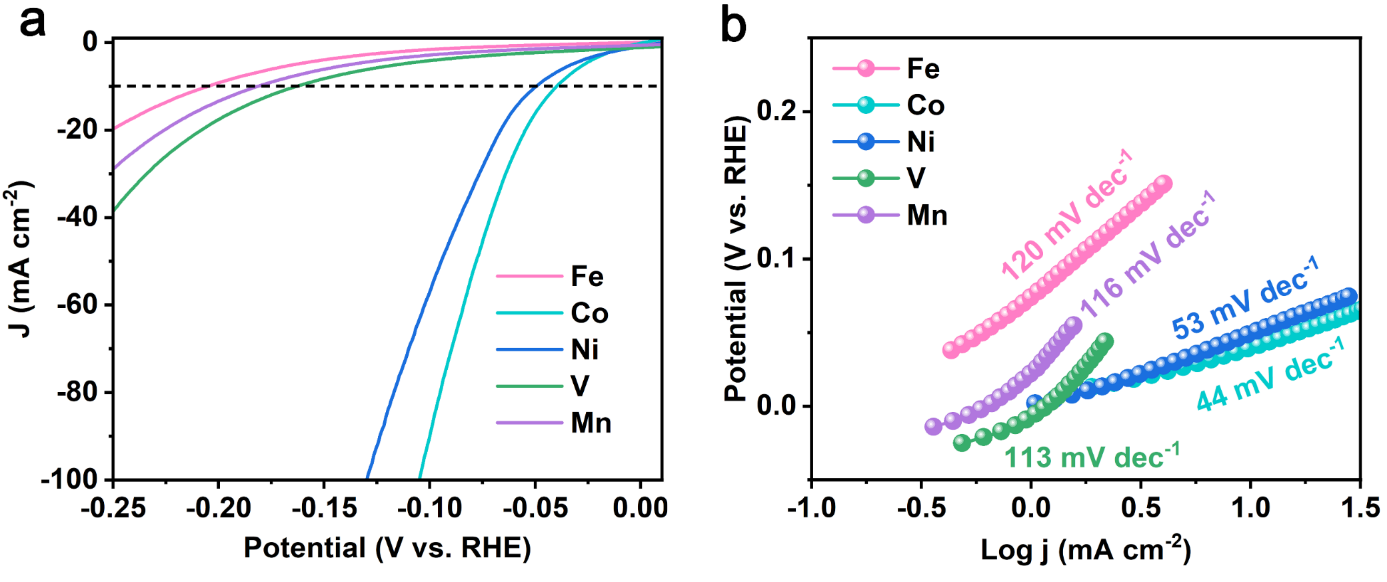


**Figure S30.** (a) LSV curves of 2H@1T-MoS_2_ with different single atoms of Fe, Co, Ni, V and Mn. (b) The Tafel slope diagram.

The HER performance of Fe, Co, Ni, V and Mn single atoms loaded on 2H@1T-MoS_2_ is compared in the range of transition metals. The overpotentials and Tafel slopes of each nanoreactor are as follows: The overpotentials of Fe, Co, Ni, V and Mn are 205 mV, 40 mV, 50 mV, 163 mV, 181 mV. The Tafel slopes of Fe, Co, Ni, V and Mn are 120 mV dec^-1^, 44 mV dec^-1^, 53 mV dec^-1^, 113 mV dec^-1^, 116 mV dec^-1^, respectively. The performance of all these single atoms is inferior to that of Sn.


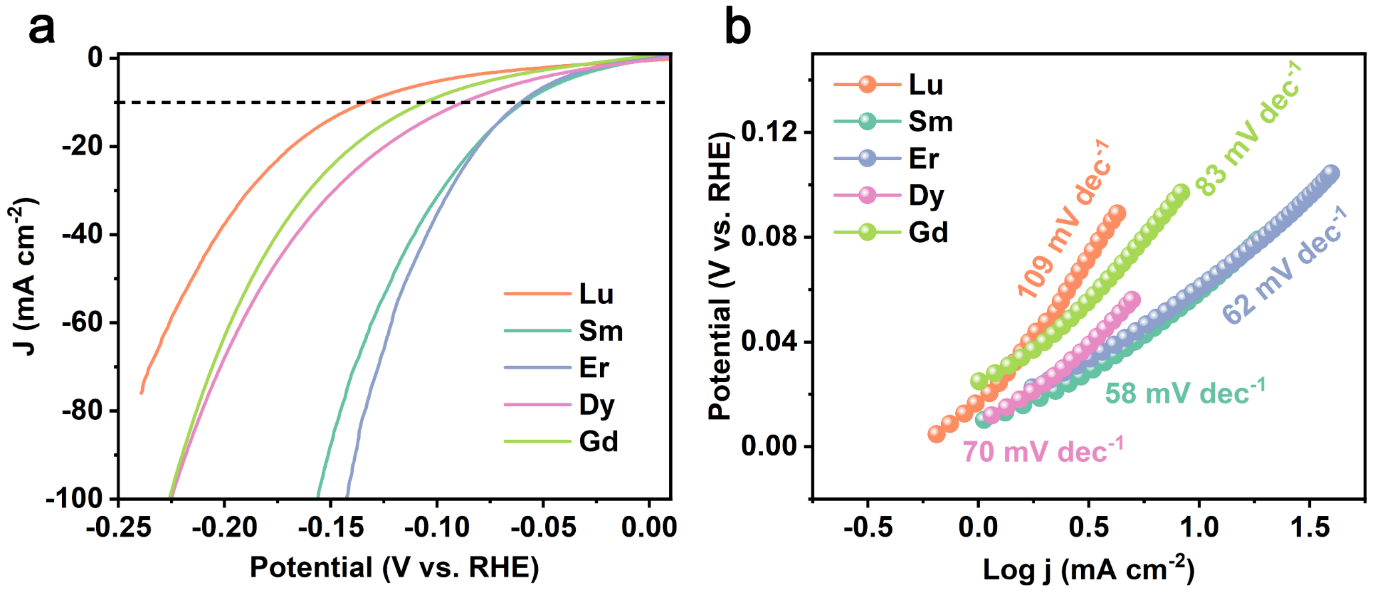


**Figure S31.** (a) Comparative LSV curves are obtained for 2H@1T-MoS_2_ modified with individual atoms of Lu, Sm, Er, Dy, and Gd. (b) The corresponding Tafel slope analysis is conducted.

The HER performance of single atoms of Lu, Sm, Er, Dy and Gd loaded on 2H@1T-MoS₂ is compared in the range of lanthanide metals. The overpotentials and Tafel slopes for each nanoreactor are as follows: The overpotentials of Lu, Sm, Er, Dy and Gd are 134 mV, 59 mV, 60 mV, 88 mV, 106 mV. The Tafel slopes of Lu, Sm, Er, Dy and Gd are 109 mV dec^-1^, 58 mV dec^-1^, 62 mV dec^-1^, 70 mV dec^-1^, 83 mV dec^-1^, respectively. The performance of all these single atoms is inferior to that of Sn.


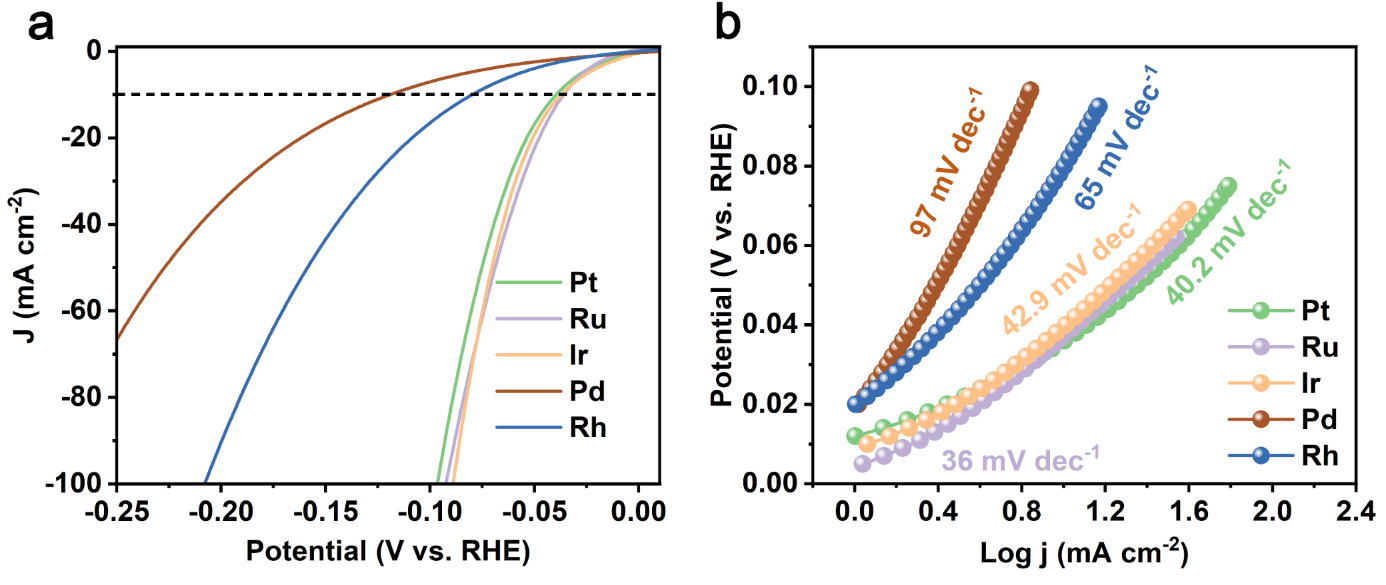


**Figure S32.** (a) LSV curves of 2H@1T-MoS_2_ with different single atoms of Pt, Ru, Ir, Pd and Rh. (b) The Tafel slope diagram.

The performance of single atoms of Pt, Ru, Ir, Pd, and Rh loaded on 2H@1T-MoS₂ is compared in the range of precious metals. The overpotentials and Tafel slopes for each nanoreactor are as follows: The overpotentials of Pt, Ru, Ir, Pd, and Rh are 40 mV, 36 mV, 37 mV, 119 mV, 80 mV. The Tafel slopes of Pt, Ru, Ir, Pd, and Rh are 40.2 mV dec^-1^, 36 mV dec^-1^, 42.9 mV dec^-1^, 97 mV dec^-1^, 65 mV dec^-1^, respectively. The performance of all these single atoms is inferior to that of Sn.


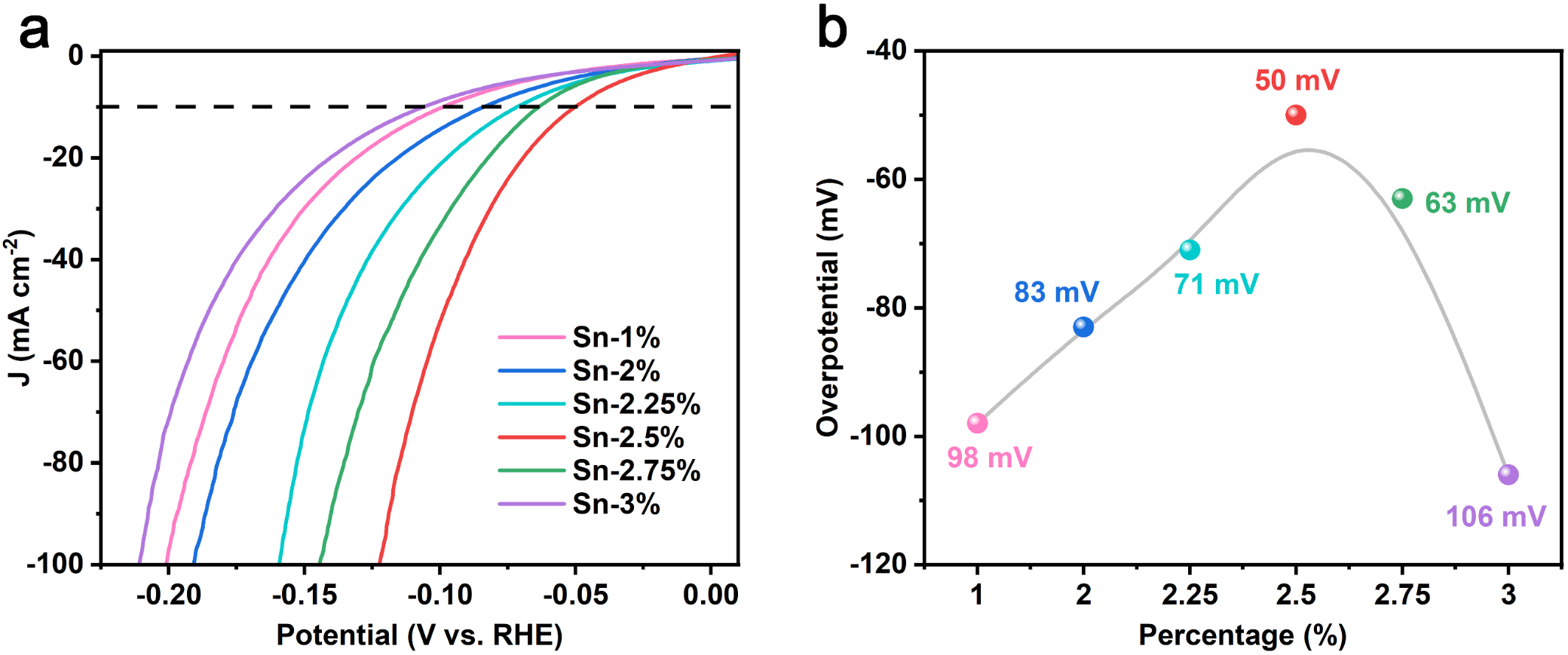


**Figure S33.** (a) LSV curves of Sn single atom content at 1:20 ratio of 2H@1T-MoS_2_ loaded with different mass ratios, (b) overpotentials at current densities of 10 mA cm^-2^.

By comparing different loads, it is found that the performance varies with the input ratio of Sn. Specifically, at an input ratio of 1%, the overpotential is 98 mV. As the ratio increases to 2%, the overpotential declines to 83 mV, and the overpotential is 71 mV at 2.25%. At an input ratio of 2.5%, the overpotential reaches its optimal value of 50 mV, corresponding to the peak of the volcanic curve. However, beyond this optimal ratio, the overpotential improves to 63 mV at 2.75% and 106 mV at 3%. Therefore, the highest performance of Sn single atoms is achieved at an input ratio of 2.5%.


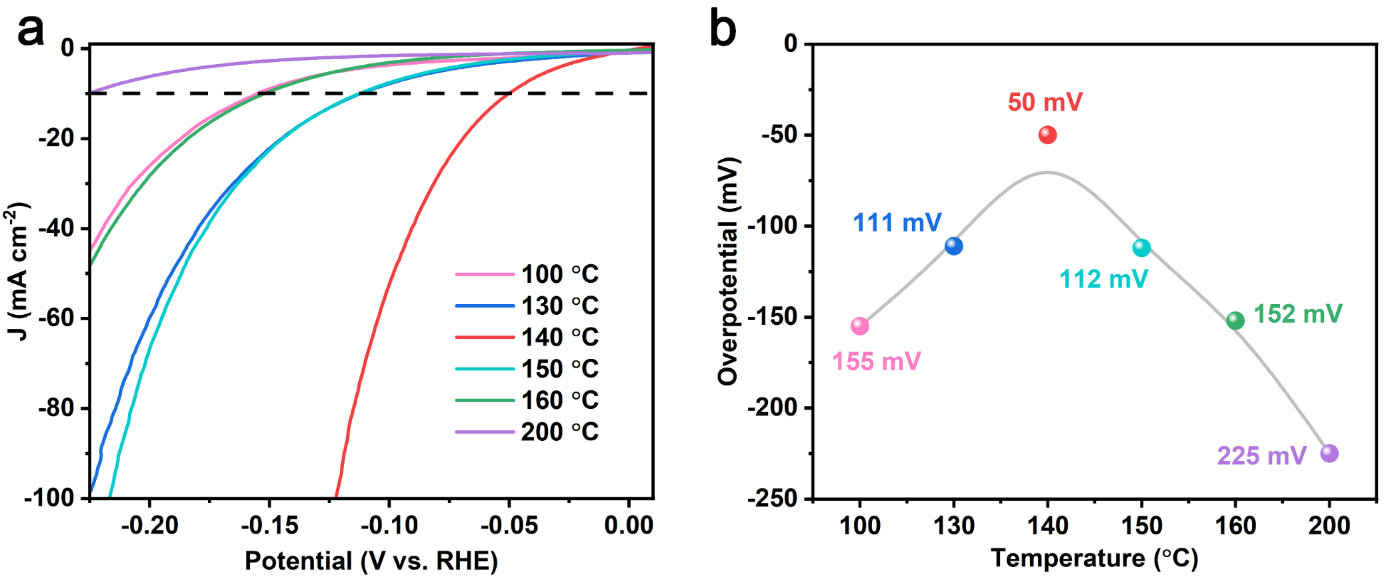


**Figure S34.** (a) LSV curves of 2H@1T-MoS_2_ with 2.5% Sn content treated at different annealing temperatures for 60 min, (b) overpotentials at current densities of 10 mA cm^-2^.

The catalytic performance is evaluated under six different annealing temperatures. At 100 °C, the overpotential is measured at 155 mV. Increasing the annealing temperature to 130 °C reduced the overpotential to 111 mV, while further raising it to 140 °C achieved the best performance with an overpotential of 50 mV. Beyond this optimal temperature, the overpotential increased to 112 mV at 150 °C, 152 mV at 160 °C, and 225 mV at 200 °C. These results confirm that annealing at 140 °C for 60 min is the optimal condition for achieving the lowest overpotential.


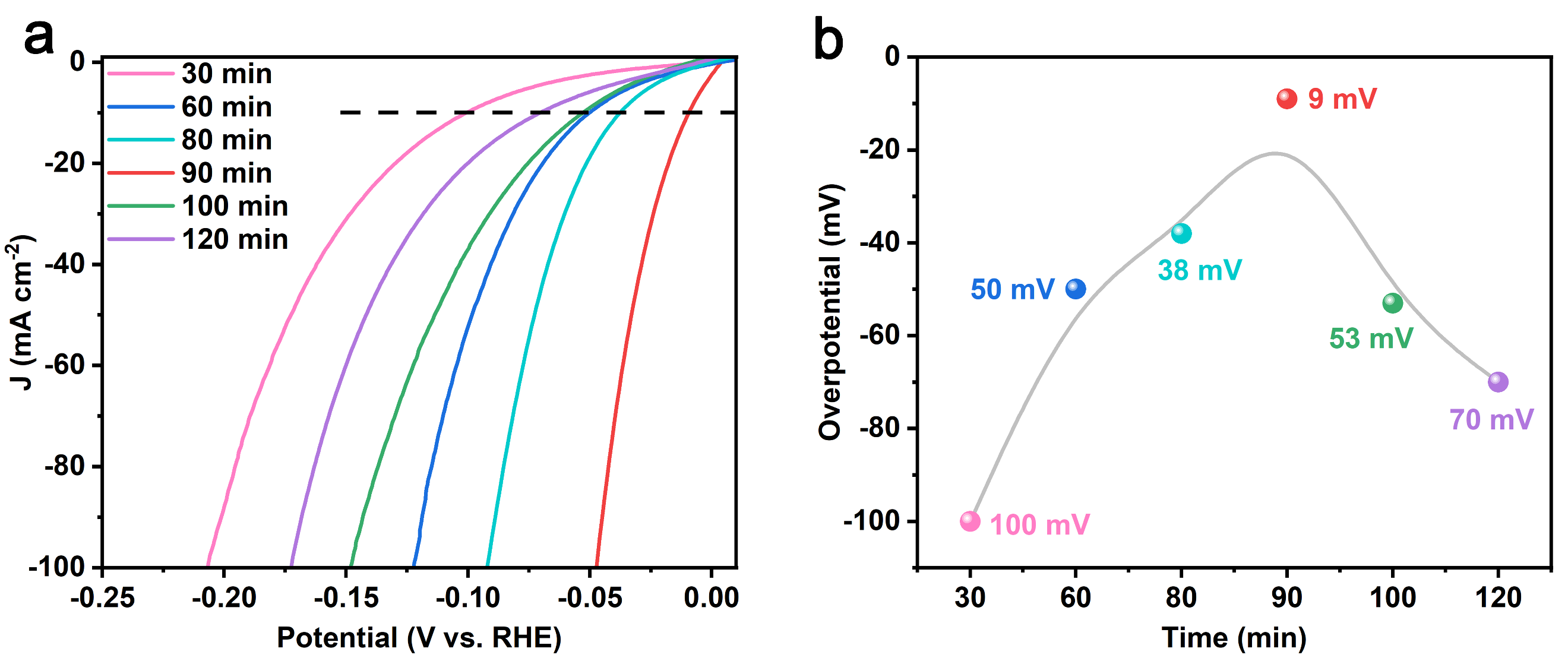


**Figure S35.** (a) LSV curves at different annealing times at 2H@1T-MoS_2_ 140 ℃ with a Sn content of 2.5%, (b) overpotentials at 10 mA cm^-2^.

By testing annealing durations at 140 °C, a clear performance trend emerges. At 30 min, performance is 100 mV, improving to 50 mV at 60 min and 38 mV at 80 min. The optimal performance of 9 mV is achieved at 90 min. Beyond this, performance declines to 53 mV at 100 min and 70 mV at 120 min, indicating a critical time with 90 min as the optimal duration.


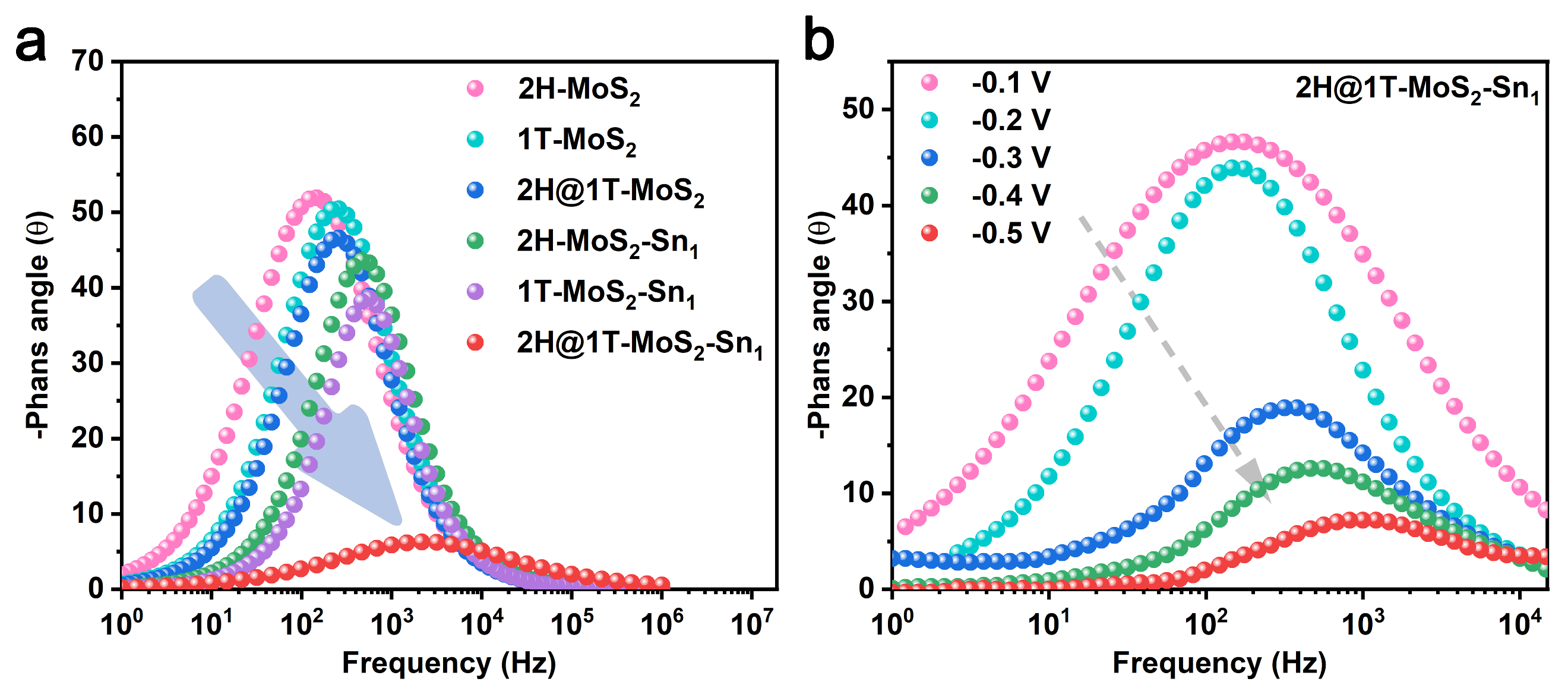


**Figure S36.** (a) Nyquist diagram; (b) Bode phase diagram of 2H@1T-MoS_2_-Sn_1_ at different voltages.

The results show that the responses in the low frequency and high frequency regions come from the interface charge transfer and inner layer electron transfer of 2H@1T-MoS_2_-Sn_1_ in Bode diagram.


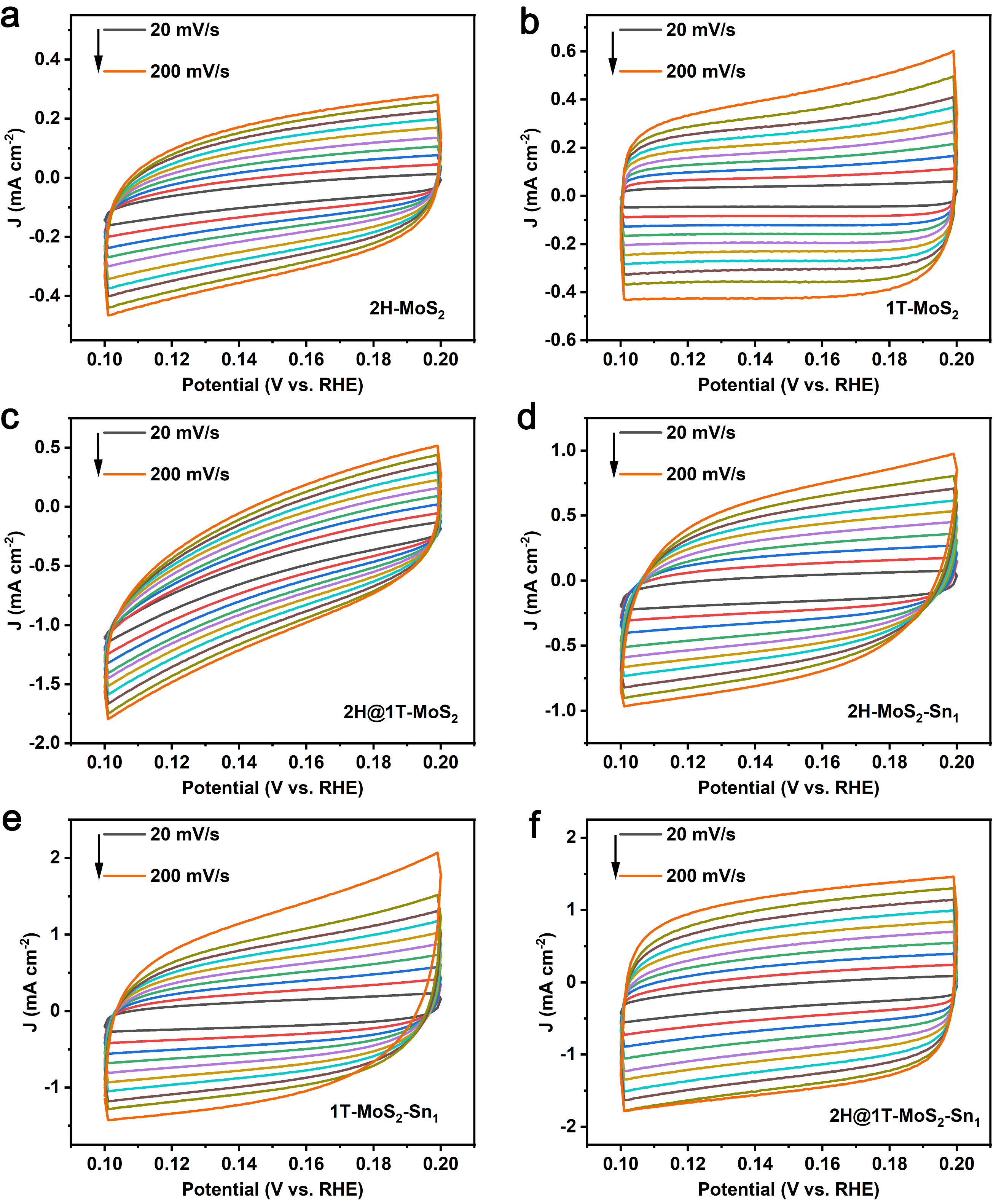


**Figure S37.** The closed CV curves of (a) 2H-MoS_2_, (b) 1T-MoS_2_, (c) 2H@1T-MoS_2_, (d) 2H-MoS_2_-Sn_1_, (e) 1T-MoS_2_-Sn_1_, (f) 2H@1T-MoS_2_-Sn_1_, the capacitive currents of all samples are collected at 0.15 V.

The CV test revealed that the current density of 2H-MoS_2_ is the lowest, while that of 2H@1T-MoS_2_-Sn_1_ is the highest over a range of scanning speeds from 0.1 to 0.2 V_RHE_.


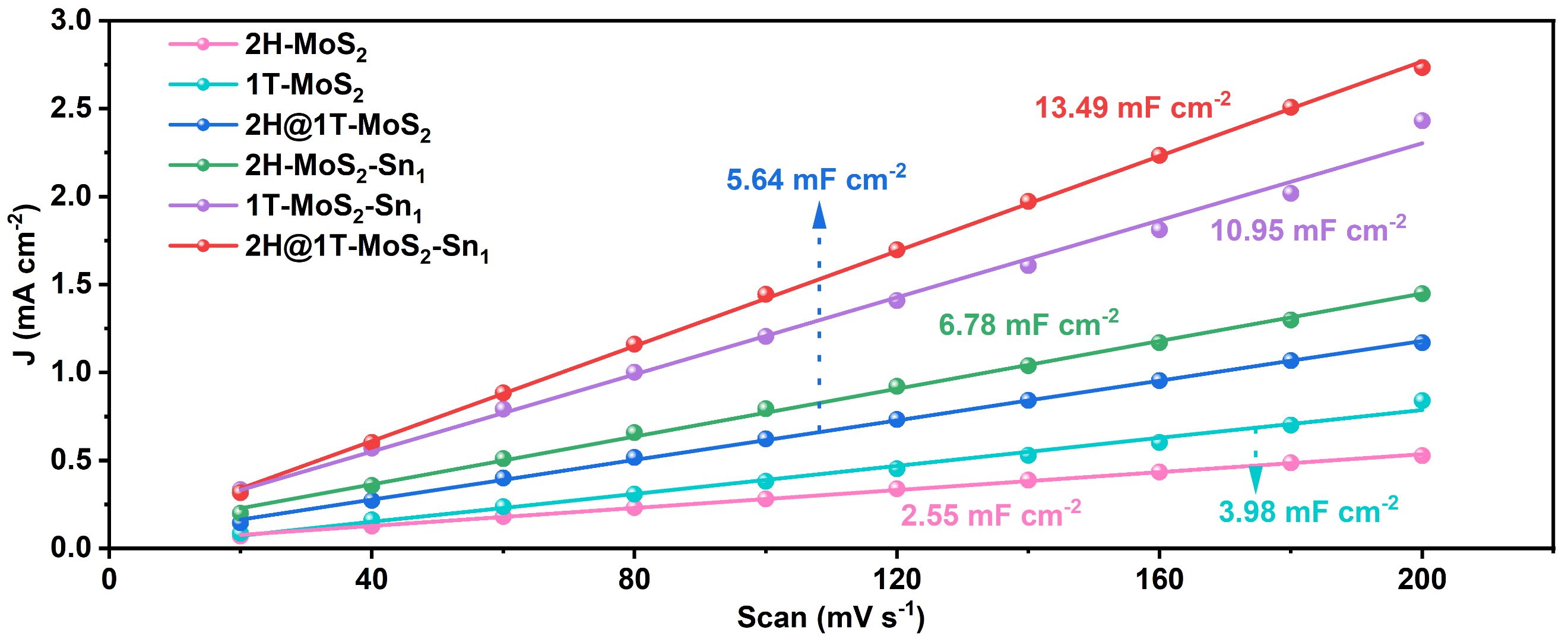


**Figure S38.** The capacitance value (C_dl_) of the 2H@1T-MoS_2_-Sn_1_ nanoreactor.

The 2H@1T-MoS_2_-Sn_1_ nanoreactor demonstrates a notable enhancement compared to other catalysts across varying scanning rates. Specifically, the C_dl_ value for 2H@1T-MoS_2_-Sn_1_ is 13.49 mF cm^-2^, marking an enhancement of 5.3 times over 2H-MoS_2_, 3.4 times over 1T-MoS_2_, 2.4 times over 2H@1T-MoS_2_, and twice the value of 2H-MoS_2_-Sn_1_. Furthermore, it surpasses 1T-MoS_2_-Sn_1_ by 1.23 times, indicating a significant boost in electrocatalytic activity following the anchoring of Sn single atoms.


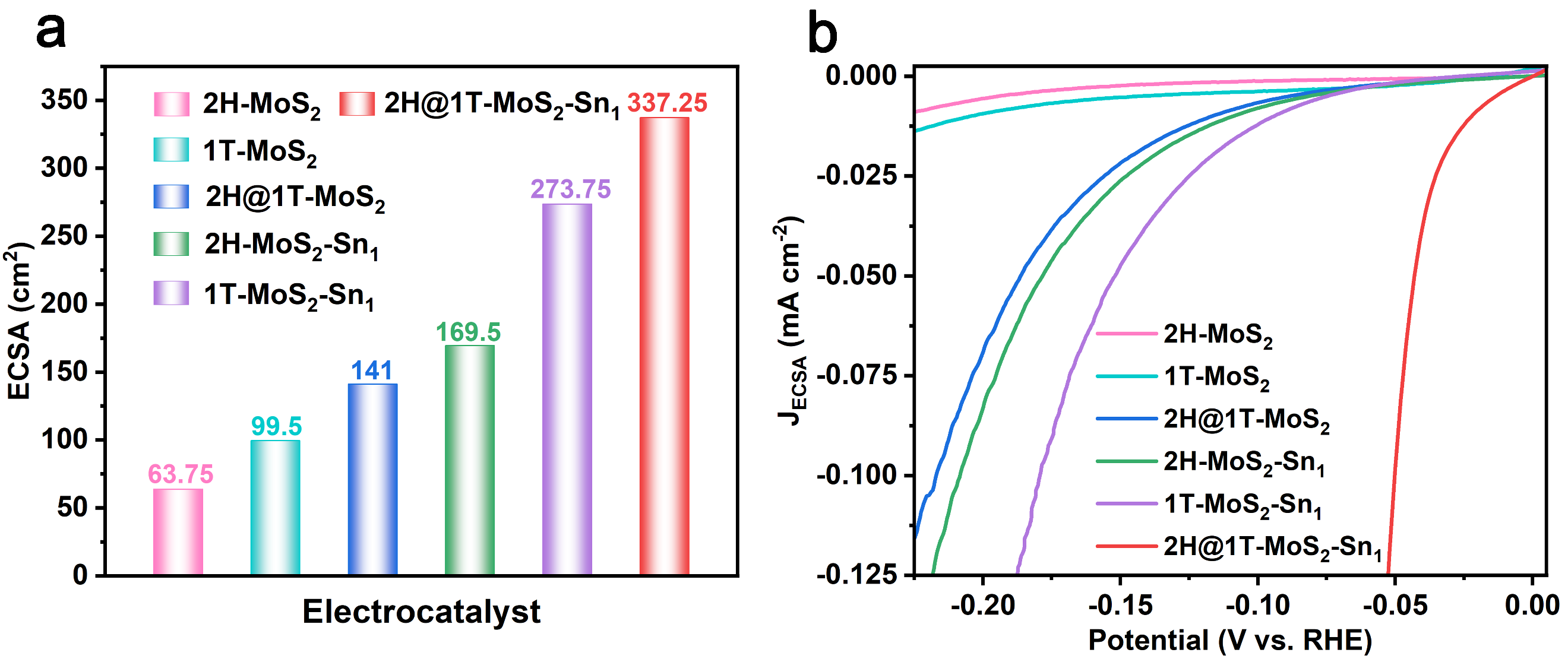


**Figure S39.** (a) ECSA of 2H-MoS_2_, 1T-MoS_2_, 2H@1T-MoS_2_, 2H-MoS_2_-Sn_1_, 1T-MoS_2_-Sn_1_ and 2H@1T-MoS_2_-Sn_1_ in 0.5 M H_2_SO_4_, (b) LSV curves of the as-synthesized catalysts normalized by ECSA.

The ECSA of 2H-MoS_2_, 1T-MoS_2_, 2H@1T-MoS_2_, 2H-MoS_2_-Sn_1_, 1T-MoS_2_-Sn_1_, and 2H@1T-MoS_2_-Sn_1_ are measured at 63.75 cm^2^, 99.5 cm^2^, 141 cm^2^, 169.5 cm^2^, 273.75 cm^2^, and 337.25 cm^2^, respectively. Through a comparison of the synthesized catalysts, it is found that the normalized 2H@1T-MoS_2_-Sn_1_ catalyst displayed the highest performance.


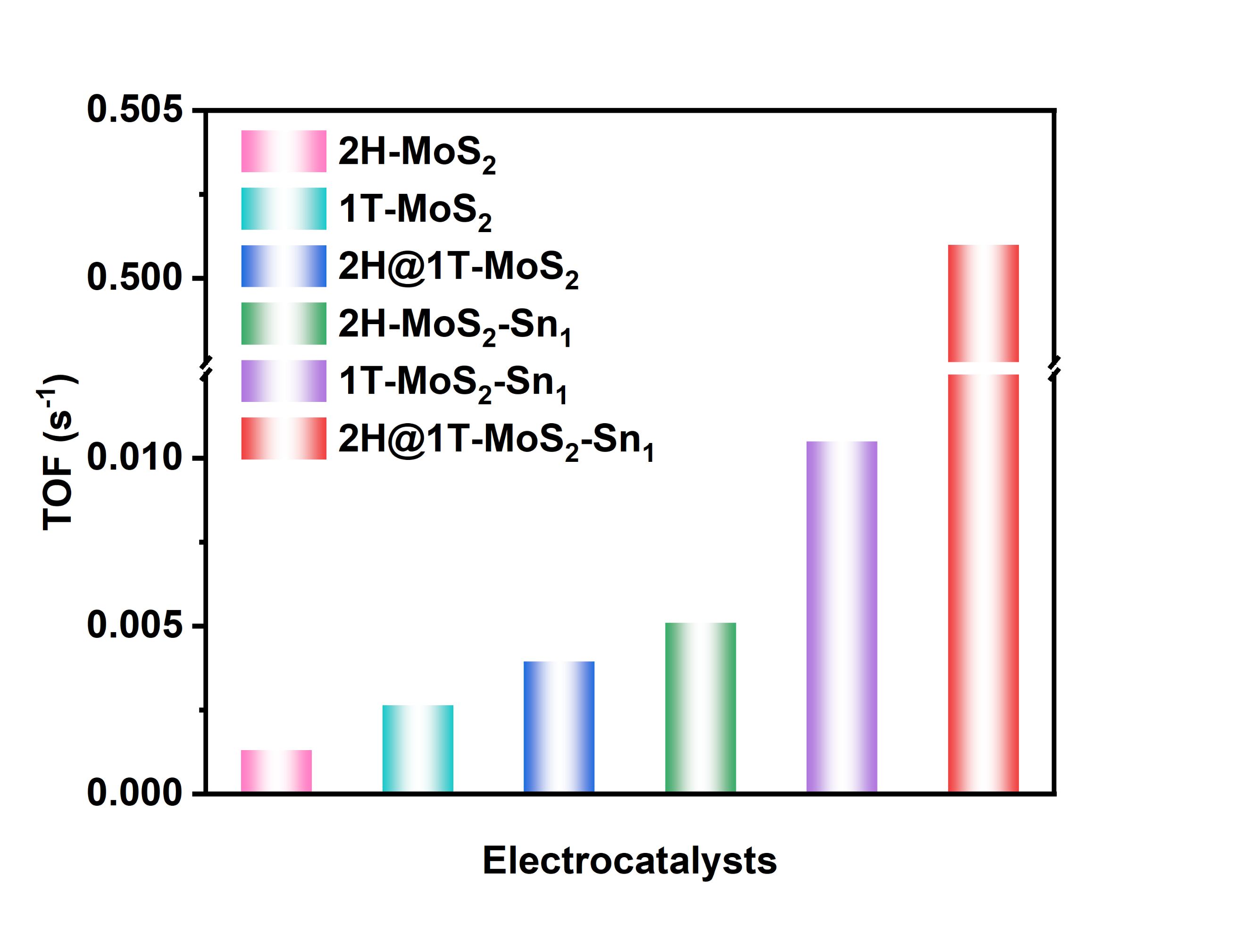


**Figure S40.** The TOF values for 2H-MoS_2_, 1T-MoS_2_, 2H@1T-MoS_2_, 2H-MoS_2_-Sn_1_, 1T-MoS_2_-Sn_1_, and 2H@1T-MoS_2_-Sn_1_ at 50 mV are respectively 0.0013 s^-1^, 0.00265 s^-1^, 0.00395 s^-1^, 0.0051 s^-1^, 0.0105 s^-1^, and 0.50 s^-1^.

The 2H@1T-MoS_2_-Sn_1_ nanoreactor exhibited a TOF value of 0.50 s^-1^ in acidic conditions, representing a 50-fold increase compared to 1T-MoS_2_-Sn_1_ and a 126-fold increase compared to 2H@1T-MoS_2_, showcasing significantly enhanced catalytic activity following the introduction of Sn single atoms.


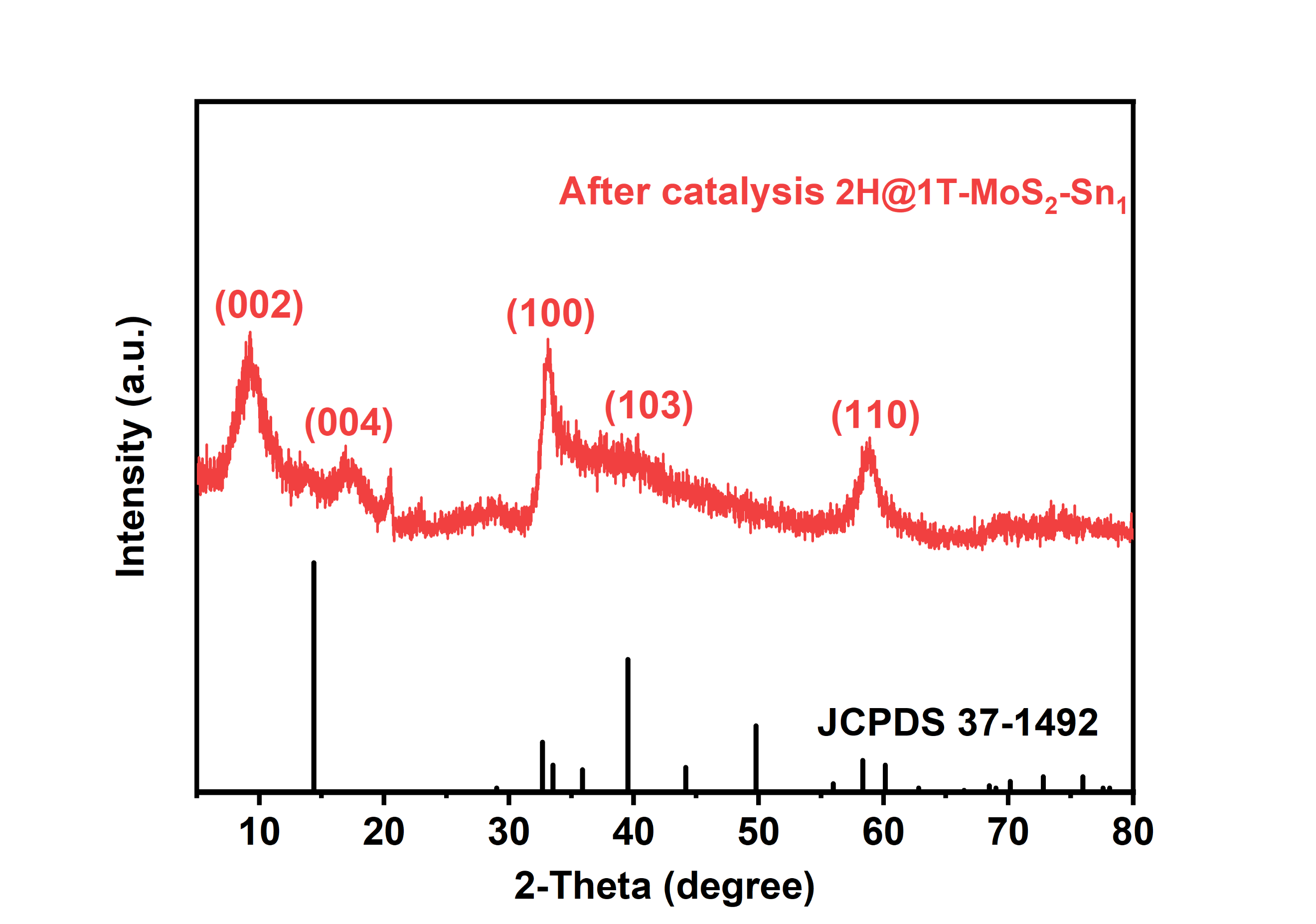


**Figure S41.** The XRD of after 2H@1T-MoS_2_-Sn_1_ reaction. (JCPDS: 37-1492).

After conducting tests on the electrocatalytic reaction process of the nanoreactor, it is observed that there are no significant changes in the intensity of the main vibration peaks at 9.16°, 32.6°, and 58.3°, indicating the stability of nanoreactor properties.


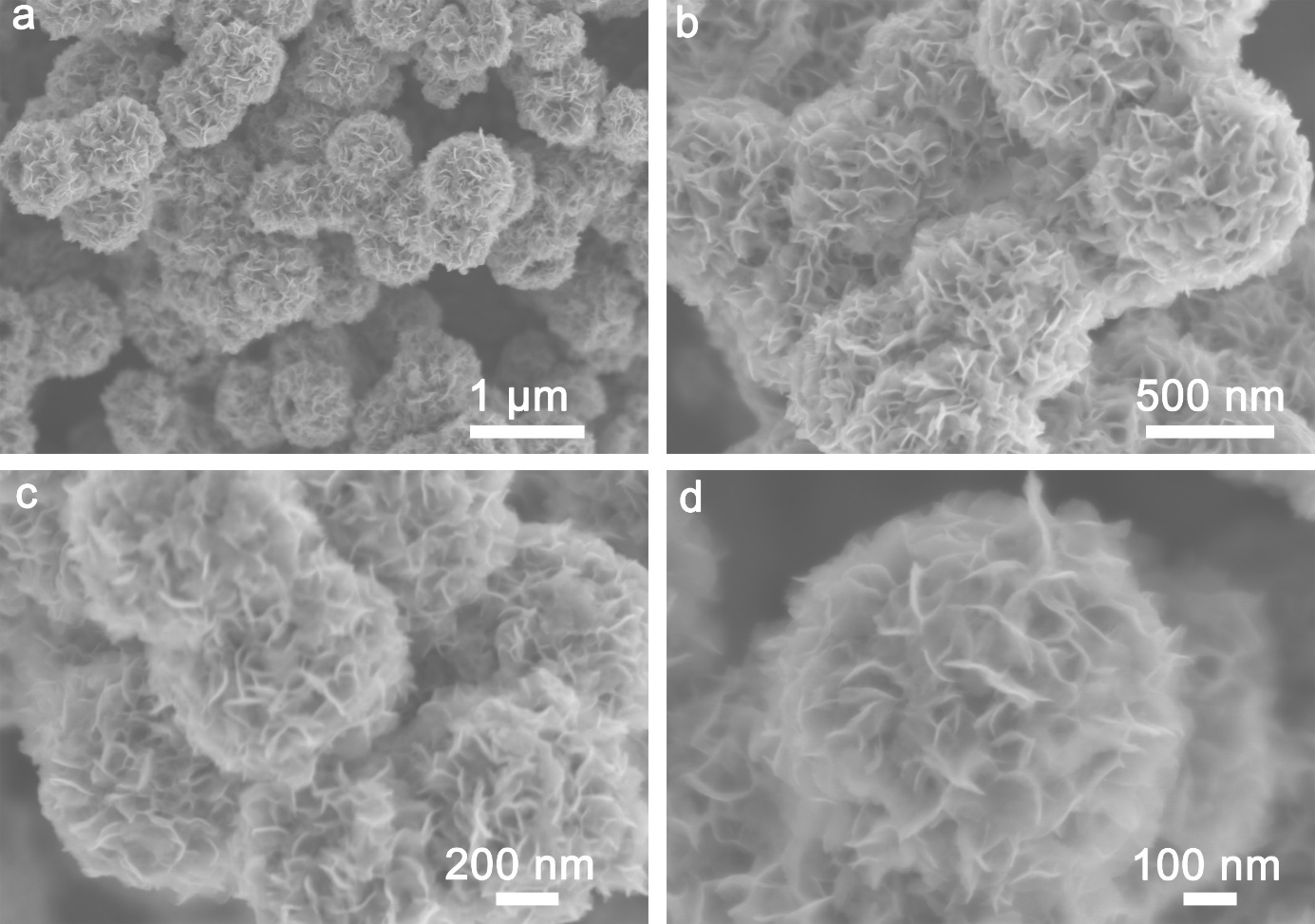


**Figure S42.** (a-d) SEM images of the 2H@1T-MoS_2_-Sn_1_ nanoreactor at various magnifications reveal that its morphology remained intact after the reaction.


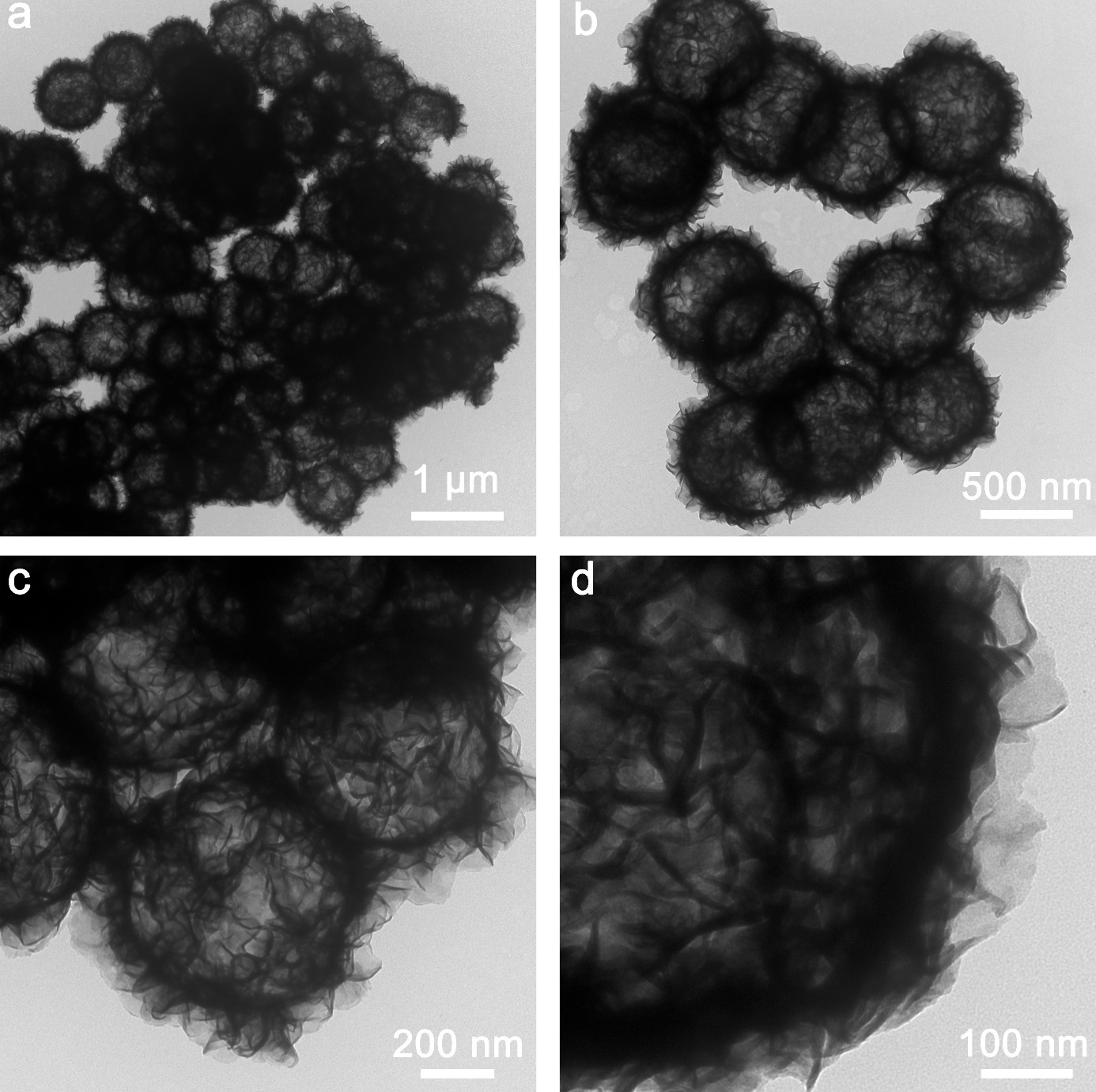


**Figure S43.** (a-d) TEM images of the 2H@1T-MoS_2_-Sn_1_ nanoreactor at various magnifications reveal that its morphology remained intact after the reaction.


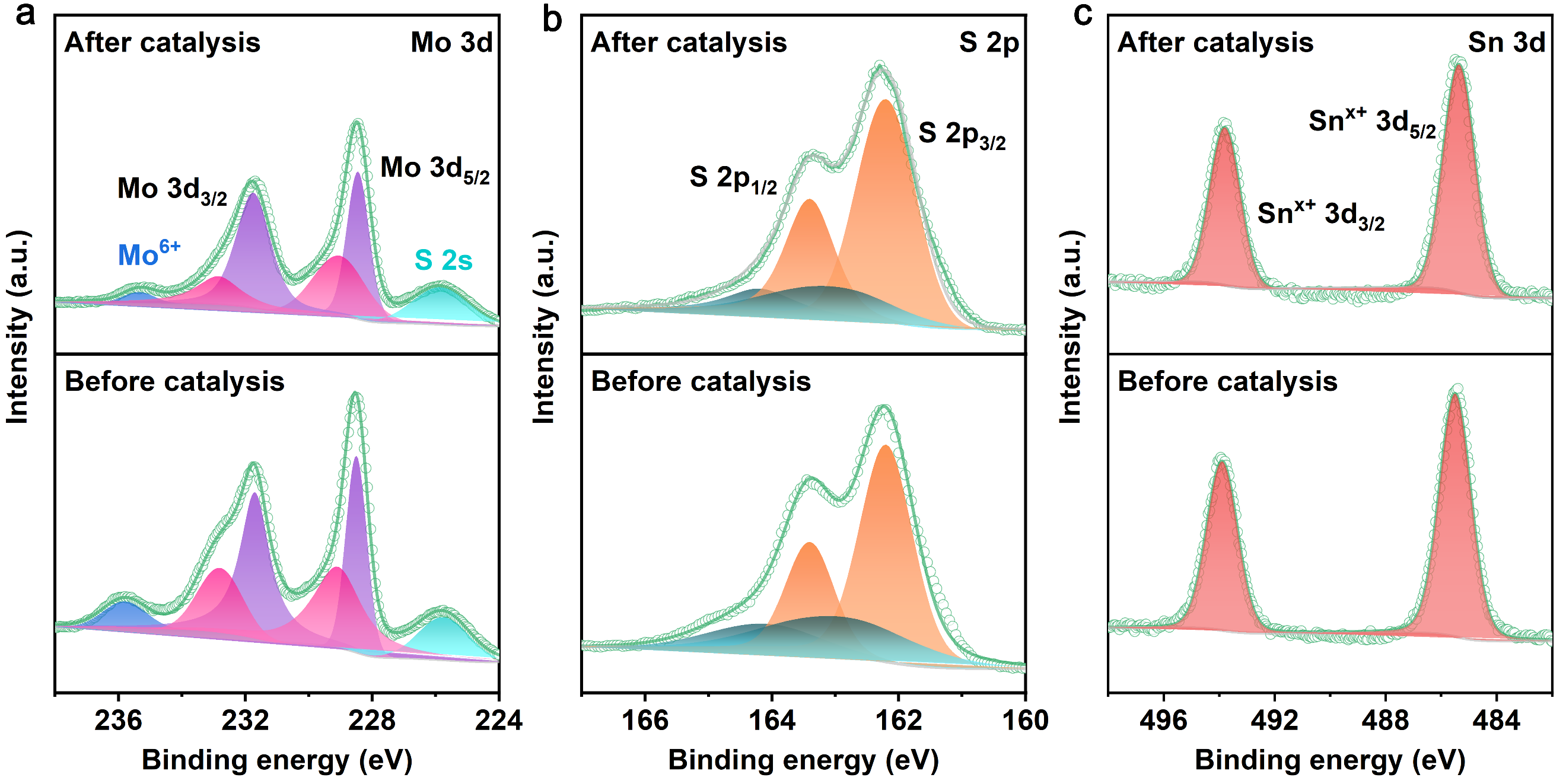


**Figure S44.** (a) The XPS of Mo 3d, (b) S 2p, (c) Sn 3d.

The stability of the 2H@1T-MoS_2_-Sn_1_ nanoreactor structure is demonstrated by the unchanged peak positions before and after the reaction.


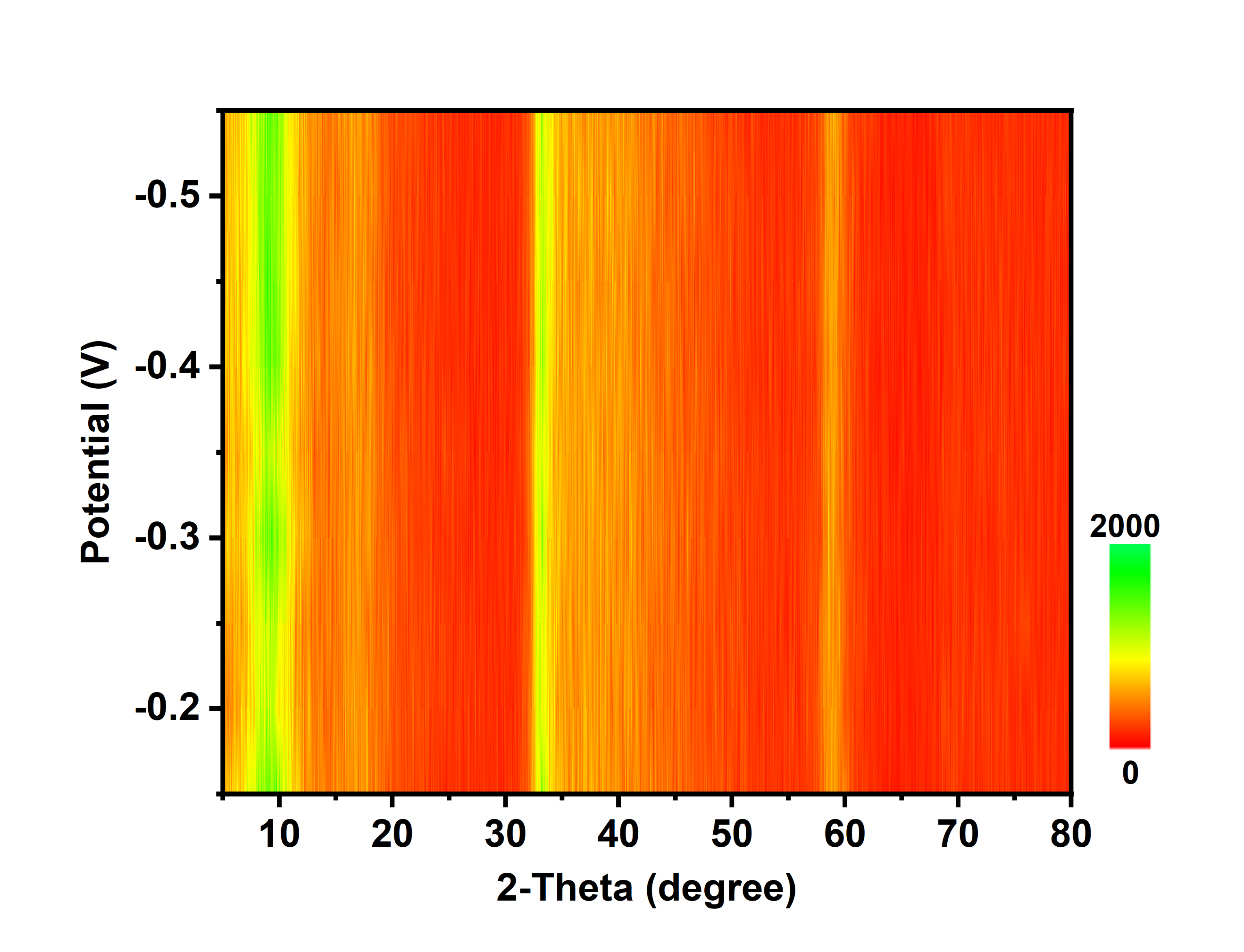


**Figure S45.** In situ XRD of 2H@1T-MoS_2_-Sn_1_ nanoreactor.

During the electrocatalytic process, no significant change in the intensity of the main vibration peaks positioned at 9.16°, 32.6°, and 58.3° is observed at different voltages, signifying the stable nature of the nanoreactor.


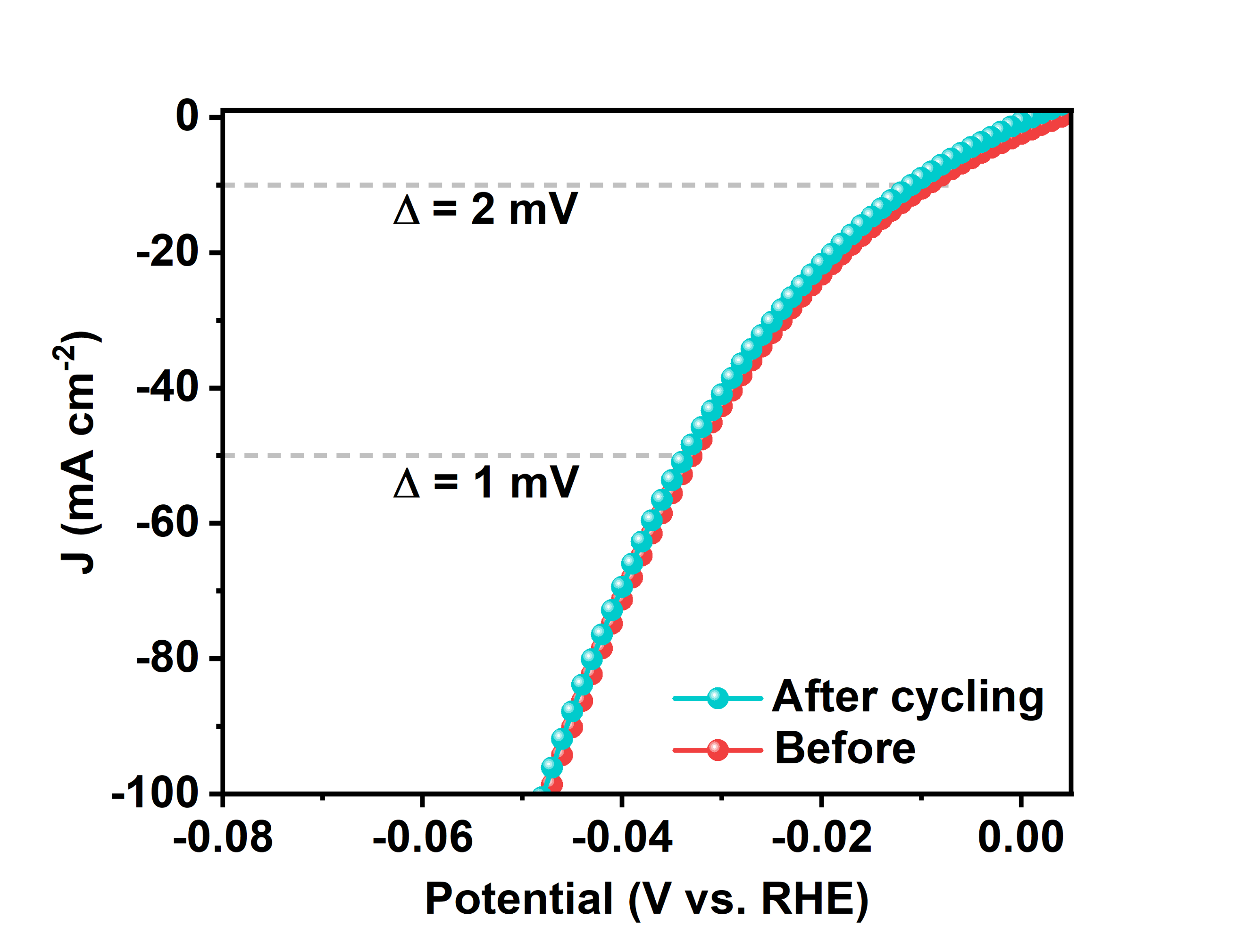


**Figure S46.** The LSV polarization curves of the 2H@1T-MoS_2_-Sn_1_ nanoreactor before and after cycling test.

The results clearly demonstrate the outstanding stability of our electrocatalyst under acidic HER conditions. Specifically, the overpotential shift at 10 mA cm^-2^ is merely 2 mV after cycling, while the difference at 50 mA cm^-2^ is also limited to 1 mV. Even at a high current density of 100 mA cm^-2^, the overpotential remains nearly identical to the initial value, confirming the structural integrity and electrochemical robustness of the 2H@1T-MoS_2_-Sn_1_ catalyst during prolonged operation.

1.11 **Tables 1-5**

**Table S1.** The ICP-MS data of Sn content in the 2H@1T-MoS_2_-Sn_1_ nanoreactor before and after reaction.

| Samples [(4 mg/50 mL) in 500 mL water+HNO_3_] | Concentrations (mg L^-1^) | Weight percentage (%) |
| --- | --- | --- |
| 2H@1T-MoS_2_-Sn_1_ (before reaction) | 0.1278 | 1.60 |
| 2H@1T-MoS_2_-Sn_1_ (after reaction) | 0.1257 | 1.57 |

**Table S2.** The comparison of the overpotentials and Tafel slopes for MoS_2_-based electrocatalysts. (acidic solutions)

| MoS_2_-based | η_10_ (mV) | Tafel (mV dec^-1^) | Ref. |
| --- | --- | --- | --- |
| 2H@1T-MoS_2_-Sn_1_ | 9 | 16.3 | This work |
| C-Co-MoS_2_ SACs | 17 | 32 | ^[1]^ |
| Pd, O@MoS_x_ | 23 | 18 | ^[2]^ |
| Co/Se-MoS_2_ | 104 | 67 | ^[3]^ |
| Co@MoS_2_ | 56 | 32 | ^[4]^ |
| Co-1T-MoS_2_ | 84 | 47 | ^[5]^ |
| Co-1T-MoS_2_ SACs | 42 | 32 | ^[6]^ |
| Fe@MoS_2_ | 88 | 75 | ^[7]^ |
| Cu-SACs/1T-MoS_2_ | 131 | 51 | ^[8]^ |
| Ni@MoS_2_ | 82 | 47.5 | ^[9]^ |
| Ni-MoS_2_ SACs | 110 | 74 | ^[10]^ |
| Zn@MoS_2_ | 194 | 78 | ^[11]^ |
| P-1T-MoS_2_ | 153 | 43 | ^[12]^ |
| Ni-Co-MoS_2_ | 155 | 51 | ^[13]^ |
| 1T-MoS_2_ QDs | 92 | 44 | ^[14]^ |
| Pd, Co@MoS_2_ | 49.3 | 43.2 | ^[15]^ |
| Pd, Cu@MoS_2_ | 93 | 77 | ^[16]^ |
| Pd@MoS_2_ | 78 | 32 | ^[17]^ |
| Pt-SAs/MoS_2_ | 44 | 34.83 | ^[18]^ |
| Pt/MoS_2_ | 88 | 55 | ^[19]^ |
| Rh-MoS_2_ SACs | 67 | 54 | ^[20]^ |
| Ru-SAs/2H-MoS_2_ | 167 | 77.5 | ^[21]^ |
| Ru@MoS_2_ | 25 | 46 | ^[22]^ |
| s-Pt/1T′-MoS_2_ | 19 | / | ^[23]^ |
| Pt_1%_–CoMoS_2_/C | 118 | 68 | ^[24]^ |
| Pd_x_S_y_/1T-MoS_2_ | 78 | 39.8 | ^[25]^ |
| 1T-MoS_2_/SWNT | 108 | 36 | ^[26]^ |
| Pt-doped MoS_2_ | 140 | 96 | ^[27]^ |
| P-doped MoS_2_ | 43 | 34 | ^[28]^ |
| MoS_2_/N-RGO | 56 | 41.3 | ^[29]^ |
| N-Carbon/MoS_2_ | 180 | 61 | ^[30]^ |
| Au/MoS_2_ | 130 | 94 | ^[31]^ |
| MSOR1 | 197 | 75.8 | ^[32]^ |
| Co-MoS_2_@CC | 130 | 92 | ^[33]^ |
| Pt/FeMn-MoS_2_ | 55.3 | / | ^[34]^ |
| Co@MoS_2_-S_V_ | 97 | 49 | ^[35]^ |
| MoO_2_/MoS_2_/C | 77 | 41 | ^[36]^ |
| Pd-MoS_2_ | 89 | 59 | ^[37]^ |
| P-BMS | 60 | 76 | ^[38]^ |

**Table S3.** The comparison of the overpotentials and Tafel slopes for MoS_2_-based electrocatalysts. (neutral solutions)

| MoS_2_-based | η_10_ (mV) | Tafel (mV dec^-1^) | Ref. |
| --- | --- | --- | --- |
| MSOR1 | 183 | 63.8 | ^[32]^ |
| Pt/FeMn-MoS_2_ | 301 | / | ^[34]^ |
| Co@MoS_2_-S_V_ | 117 | 55 | ^[35]^ |
| MoO_2_/MoS_2_/C | 97 | 53 | ^[36]^ |
| N, Mn co-doped MoS_2_ | 70 | 65 | ^[39]^ |
| MoS_2_@CoS_2_ | 164 | 42 | ^[40]^ |
| MoS_2_/MoP@NC | 103 | 87.4 | ^[41]^ |
| Ni-CoS_2_@MoS_2_ | 196 | 93.3 | ^[42]^ |
| MoP_2_/MoS_2_ | 89.83 | 73.79 | ^[43]^ |

**Table S4.** The comparison of the overpotentials and Tafel slopes for MoS_2_-based electrocatalysts. (alkaline solutions)

| MoS_2_-based | η_10_ (mV) | Tafel (mV dec^-1^) | Ref. |
| --- | --- | --- | --- |
| MSOR1 | 43 | 63.1 | ^[32]^ |
| Pt/FeMn-MoS_2_ | 34.1 | 51.1 | ^[34]^ |
| Co@MoS_2_-S_V_ | 36 | 33 | ^[35]^ |
| MoO_2_/MoS_2_/C | 91 | 49 | ^[36]^ |
| N, Mn co-doped MoS_2_ | 60 | 55 | ^[39]^ |
| O-MoS_2_ | 120 | 87 | ^[44]^ |
| Co-MoS_2_@CC | 56.6 | 62.6 | ^[45]^ |
| Co-MoS_2_ | 92 | 82 | ^[46]^ |
| Re-MoS_2_-Vs | 99 | 89 | ^[47]^ |
| MoS_2_-MoP/NC | 35 | 30 | ^[48]^ |
| Ni-1T-MoS_2_ | 91 | 52.7 | ^[49]^ |

**Table S5.** Summarized parameters of EIS for the MoS_2_-based electrocatalysts.

| Samples | R_CT_ (Ω cm^2^) | C_CT_ (F cm^-2^) |
| --- | --- | --- |
| 2H-MoS_2_ | 38.04 | 1.6264E^-4^ |
| 1T-MoS_2_ | 26.75 | 8.978E^-5^ |
| 2H@1T-MoS_2_ | 20.94 | 1.0087E^-4^ |
| 2H-MoS_2_-Sn_1_ | 15.44 | 4.74E^-5^ |
| 1T-MoS_2_-Sn_1_ | 10.97 | 4.5155E^-5^ |
| 2H@1T-MoS_2_-Sn_1_ | 1.42 | 1.0302E^-3^ |

References

[1] F. Gong, Y. Liu, Y. Zhao, W. Liu, G. Zeng, G. Wang, Y. Zhang, L. Gong, J. Liu; Universal Sub-Nanoreactor Strategy for Synthesis of Yolk-Shell MoS_2_ Supported Single Atom Electrocatalysts Toward Robust Hydrogen Evolution Reaction. *Angew. Chem. Int. Ed.* **2023**, *62*, e202308091.

[2] Y. Zhan, X. Zhou, H. Nie, X. Xu, X. Zheng, J. Hou, H. Duan, S. Huang, Z. Yang; Designing Pd/O co-Doped MoS_x_ for Boosting the Hydrogen Evolution Reaction. *J. Mater. Chem. A* **2019**, *7*, 15599-15606.

[3] Z. Zheng, L. Yu, M. Gao, X. Chen, W. Zhou, C. Ma, L. Wu, J. Zhu, X. Meng, J. Hu, Y. Tu, S. Wu, J. Mao, Z. Tian, D. Deng; Boosting Hydrogen Evolution on MoS_2_ via co-Confining Selenium in Surface and Cobalt in Inner Layer. *Nat. Commun.* **2020**, *11*, 3315.

[4] Q. Jin, N. Liu, C. Dai, R. Xu, B. Wu, G. Yu, B. Chen, Y. Du; H_2_-Directing Strategy on in situ Synthesis of Co-MoS_2_ with Highly Expanded Interlayer for Elegant HER Activity and its Mechanism. *Adv. Energy Mater.* **2020**, *10*, 2000291.

[5] W. Qiao, W. Xu, X. Xu, L. Wu, S. Yan, D. Wang; Construction of Active Orbital via Single-Atom Cobalt Anchoring on the Surface of 1T-MoS_2_ Basal Plane Toward Efficient Hydrogen Evolution. *ACS Appl. Energy Mater.* **2020**, *3*, 2315-2322.

[6] K. Qi, X. Cui, L. Gu, S. Yu, X. Fan, M. Luo, S. Xu, N. Li, L. Zheng, Q. Zhang; Single-Atom Cobalt Array Bound to Distorted 1T-MoS_2_ with Ensemble Effect for Hydrogen Evolution Catalysis. *Nat. Commun.* **2019**, *10*, 5231.

[7] D. Vikraman, S. Hussain, K. Karuppasamy, A. Kathalingam, E.-B. Jo, A. Sanmugam, J. Jung, H.-S. Kim; Engineering the Active Sites Tuned MoS_2_ Nanoarray Structures by Transition Metal Doping for Hydrogen Evolution and Supercapacitor Applications. *J. Alloys Compd.* **2022**, *893*, 162271.

[8] L. Ji, P. Yan, C. Zhu, C. Ma, W. Wu, C. Wei, Y. Shen, S. Chu, J. Wang, Y. Du; One-Pot Synthesis of Porous 1T-phase MoS_2_ Integrated with Single-Atom Cu Doping for Enhancing Electrocatalytic Hydrogen Evolution Reaction. *Appl. Catal., B* **2019**, *251*, 87-93.

[9] S. A. Shah, X. Shen, M. Xie, G. Zhu, Z. Ji, H. Zhou, K. Xu, X. Yue, A. Yuan, J. Zhu; Nickel@ Nitrogen-Doped Carbon@MoS_2_ Nanosheets: an Efficient Electrocatalyst for Hydrogen Evolution Reaction. *Small* **2019**, *15*, 1804545.

[10] Q. Wang, Z. L. Zhao, S. Dong, D. He, M. J. Lawrence, S. Han, C. Cai, S. Xiang, P. Rodriguez, B. Xiang, Z. Wang, Y. Liang, M. Gu; Design of Active Nickel Single-Atom Decorated MoS_2_ as a pH-Universal Catalyst for Hydrogen Evolution Reaction. *Nano Energy* **2018**, *53*, 458-467.

[11] W. Wu, C. Niu, C. Wei, Y. Jia, C. Li, Q. Xu; Activation of MoS_2_ Basal Planes for Hydrogen Evolution by Zinc. *Angew. Chem. Int. Ed.* **2019**, *58*, 2029-2033.

[12] Y. Yin, J. Han, Y. Zhang, X. Zhang, P. Xu, Q. Yuan, L. Samad, X. Wang, Y. Wang, Z. Zhang; Contributions of Phase, Sulfur Vacancies, and Edges to the Hydrogen Evolution Reaction Catalytic Activity of Porous Molybdenum Disulfide Nanosheets. *J. Am. Chem. Soc.* **2016**, *138*, 7965-7972.

[13] X.-Y. Yu, Y. Feng, Y. Jeon, B. Guan, X. W. Lou, U. Paik; Formation of Ni-Co-MoS_2_ Nanoboxes with Enhanced Electrocatalytic Activity for Hydrogen Evolution. *Adv. Mater.* **2016**, *28*, 9006-9011.

[14] W. Chen, J. Gu, Q. Liu, R. Luo, L. Yao, B. Sun, W. Zhang, H. Su, B. Chen, P. Liu; Quantum Dots of 1T Phase Transitional Metal Dichalcogenides Generated via Electrochemical Li Intercalation. *ACS nano* **2018**, *12*, 308-316.

[15] W. Yang, S. Zhang, Q. Chen, C. Zhang, Y. Wei, H. Jiang, Y. Lin, M. Zhao, Q. He, X. Wang; Conversion of Intercalated MoO_3_ to Multi-Heteroatoms-Doped MoS_2_ with High Hydrogen Evolution Activity. *Adv. Mater.* **2020**, *32*, 2001167.

[16] D. Han, Z. Luo, Y. Li, N. Gao, J. Ge, C. Liu, W. Xing; Synergistic Engineering of MoS_2_ via Dual-Metal Doping Strategy Towards Hydrogen Evolution Reaction. *Appl. Surf. Sci.* **2020**, *529*, 147117.

[17] Z. Luo, Y. Ouyang, H. Zhang, M. Xiao, J. Ge, Z. Jiang, J. Wang, D. Tang, X. Cao, C. Liu; Chemically Activating MoS_2_ via Spontaneous Atomic Palladium Interfacial Doping Towards Efficient Hydrogen Evolution. *Nat. Commun.* **2018**, *9*, 2120.

[18] J. Zhu, Y. Tu, L. Cai, H. Ma, Y. Chai, L. Zhang, W. Zhang; Defect-Assisted Anchoring of Pt Single Atoms on MoS_2_ Nanosheets Produces High-Performance Catalyst for Industrial Hydrogen Evolution Reaction. *Small* **2022**, *18*, 2104824.

[19] Y. Li, Q. Gu, B. Johannessen, Z. Zheng, C. Li, Y. Luo, Z. Zhang, Q. Zhang, H. Fan, W. Luo; Synergistic Pt Doping and Phase Conversion Engineering in Two-Dimensional MoS_2_ for Efficient Hydrogen Evolution. *Nano Energy* **2021**, *84*, 105898.

[20] X. Meng, C. Ma, L. Jiang, R. Si, X. Meng, Y. Tu, L. Yu, X. Bao, D. Deng; Distance Synergy of MoS_2_-Confined Rhodium Atoms for Highly Efficient Hydrogen Evolution. *Angew. Chem. Int. Ed.* **2020**, *132*, 10588-10593.

[21] J. Wang, W. Fang, Y. Hu, Y. Zhang, J. Dang, Y. Wu, B. Chen, H. Zhao, Z. Li; Single Atom Ru Doping 2H-MoS_2_ as Highly Efficient Hydrogen Evolution Reaction Electrocatalyst in a Wide pH Range. *Appl. Catal., B* **2021**, *298*, 120490.

[22] Y. Xu, X. Jiang, G. Shao, H. Xiang, S. Si, X. Li, T. S. Hu, G. Hong, S. Dong, H. Li; Interface Effect of Ru-MoS_2_ Nanoflowers on Lignin Substrate for Enhanced Hydrogen Evolution Activity. *Energy Environ. Mater.* **2021**, *4*, 117-125.

[23] Z. Shi, X. Zhang, X. Lin, G. Liu, C. Ling, S. Xi, B. Chen, Y. Ge, C. Tan, Z. Lai, Z. Huang, X. Ruan, L. Zhai, L. Li, Z. Li, X. Wang, G. H. Nam, J. Liu, Q. He, Z. Guan, J. Wang, C. S. Lee, A. R. J. Kucernak, H. Zhang; Phase-Dependent Growth of Pt on MoS_2_ for Highly Efficient H_2_ Evolution. *Nature* **2023**, *621*, 300-305.

[24] L. A. Zavala, K. Kumar, V. Martin, F. Maillard, F. Maugé, X. Portier, L. Oliviero, L. Dubau; Direct Evidence of the Role of Co or Pt, Co Single-Atom Promoters on the Performance of MoS_2_ Nanoclusters for the Hydrogen Evolution Reaction. *ACS Catal.* **2023**, *13*, 1221-1229.

[25] H. D. Mai, S. Jeong, G. N. Bae, N. M. Tran, J. S. Youn, C. M. Park, K. J. Jeon; Pd Sulfidation-Induced 1T-Phase Tuning in Monolayer MoS_2_ for Hydrogen Evolution Reaction. *Adv. Energy Mater.* **2023**, *13* 2300183.

[26] Q. Liu, Q. Fang, W. Chu, Y. Wan, X. Li, W. Xu, M. Habib, S. Tao, Y. Zhou, D. Liu; Electron-Doped 1T-MoS_2_ via Interface Engineering for Enhanced Electrocatalytic Hydrogen Evolution. *Chem. Mater.* **2017**, *29*, 4738-4744.

[27] J. Deng, H. Li, J. Xiao, Y. Tu, D. Deng, H. Yang, H. Tian, J. Li, P. Ren, X. Bao; Triggering the Electrocatalytic Hydrogen Evolution Activity of the Inert Two-Dimensional MoS_2_ Surface via Single-Atom Metal Doping. *Energy Environ. Sci.* **2015**, *8*, 1594-1601.

[28] P. Liu, J. Zhu, J. Zhang, P. Xi, K. Tao, D. Gao, D. Xue; P Popants Triggered New Basal Plane Active Sites and Enlarged Interlayer Spacing in MoS_2_ Nanosheets Toward Electrocatalytic Hydrogen Evolution. *ACS Energy Lett.* **2017**, *2*, 745-752.

[29] Y. J. Tang, Y. Wang, X. L. Wang, S. L. Li, W. Huang, L. Z. Dong, C. H. Liu, Y. F. Li, Y. Q. Lan; Molybdenum Disulfide/Nitrogen-Doped Reduced Graphene Oxide Nanocomposite with Enlarged Interlayer Spacing for Electrocatalytic Hydrogen Evolution. *Adv. Energy Mater.* **2016**, *6*, 1600116.

[30] X. Dai, K. Du, Z. Li, M. Liu, Y. Ma, H. Sun, X. Zhang, Y. Yang; Co-Doped MoS_2_ Nanosheets with the Dominant CoMoS Phase Coated on Carbon as an Excellent Electrocatalyst for Hydrogen Evolution. *ACS Appl. Mater. Interfaces* **2015**, *7*, 27242-27253.

[31] D. Vikraman, K. Akbar, S. Hussain, G. Yoo, J.-Y. Jang, S.-H. Chun, J. Jung, H. J. Park; Direct Synthesis of Thickness-Tunable MoS_2_ Quantum Dot Thin Layers: Optical, Structural and Electrical Properties and Their Application to Hydrogen Evolution. *Nano Energy* **2017**, *35*, 101-114.

[32] Y. Zhang, T. Yang, J. Li, Q. Zhang, B. Li, M. Gao; Construction of Ru, O co-Doping MoS_2_ for Hydrogen Evolution Reaction Electrocatalyst and Surface-Enhanced Raman Scattering Substrate: High-Performance, Recyclable, and Durability Improvement. *Adv. Funct. Mater.* **2023**, *33*, 2210939.

[33] Q. Wei, D. Wang, L. Zhang, L. Zhao, B. Zhang, G. Zhou, Y. Zhao; Fabrication of Co Doped MoS_2_ Nanosheets with Enlarged Interlayer Spacing as Efficient and pH-Universal Bifunctional Electrocatalyst for Overall Water Splitting. *Ceram. Int.* **2021**, *47*, 24501-24510.

[34] X. Ding, X. Li, X. Lv, Y.-Z. Zheng, Q. Wu, H. Ding, J. Wu, R. Li, X. Tao; Composition Engineering-Triggered Bifunctionality of Free-Standing Coral-Like 1T-MoS_2_ for Highly Efficient Overall Water Splitting. *Energy Technol.* **2020**, *8*, 2000268.

[35] P. A. Koudakan, C. Wei, A. Mosallanezhad, B. Liu, Y. Fang, X. Hao, Y. Qian, G. Wang; Constructing Reactive Micro-Environment in Basal Plane of MoS_2_ for pH-Universal Hydrogen Evolution Catalysis.*Small* **2022**, *18*, 2107974.

[36] F. Gong, M. Liu, S. Ye, L. Gong, G. Zeng, L. Xu, X. Zhang, Y. Zhang, L. Zhou, S. Fang; All-pH Stable Sandwich‐Structured MoO_2_/MoS_2_/C Hollow Nanoreactors for Enhanced Electrochemical Hydrogen Evolution. *Adv. Funct. Mater.* **2021**, *31*, 2101715.

[37] J. Gupta, D. Das, P. H. Borse, B. V. Sarada; In situ Pd-Doped MoS2 Nanosheets as an HER Electrocatalyst for Enhanced Electrocatalytic Water Splitting. *Sustainable Energy Fuels* **2024**, *8*, 1526-1539.

[38] Y. Qian, J. Yu, Z. Lyu, Q. Zhang, T. H. Lee, H. Pang, D. J. Kang; Durable Hierarchical Phosphorus-Doped Biphase MoS_2_ Electrocatalysts with Enhanced H* Adsorption. *Carbon Energy* **2024**, 6, e376.

[39] T. Sun, J. Wang, X. Chi, Y. Lin, Z. Chen, X. Ling, C. Qiu, Y. Xu, L. Song, W. Chen, C. Su; Engineering the Electronic Structure of MoS_2_ Nanorods by N and Mn Dopants for Ultra-Efficient Hydrogen Production. *ACS Catal.* **2018**, *8*, 7585-7592.

[40] Y.-H. Gu, M.-F. Shao, J. Zhang, R. Li, N. Huang, Q. Liu, J.-G. Zhao, W.-Y. Zhang, X.-H. Zhang, F. Peng, W.-q. Li, J. Li; Interfacial Engineering of MoS_2_@CoS_2_ Heterostructure Electrocatalysts for Effective pH-Universal Hydrogen Evolution Reaction. *Langmuir* **2024**, *40*, 10518-10525.

[41] M. Zhu, L. Yu, S. Sha, R. Ge, C. Cheng, L. Dai, S. Li, B. Liu, Z. Qu, W. Li; Highly Efficient Nanosized MoS_2_/MoP Heterocatalyst for Enhancing Hydrogen Evolution Reaction Over a Wide pH Range. *Sustainable Mater.Technol.* **2024**, *41*, e01090.

[42] X. Qian, J. Wu, Y. Yang, W. Zhang, H. Zheng, J. Xia, M. Chen, W. Chen; Ni-CoS_2_@MoS_2_ Hollow Nanorod Array in-situ Synthesized on Ti Foil as Pt-Free Self-Supporting Electrode for Efficient Wide-pH Hydrogen Evolution. *Appl. Surf. Sci.* **2024**, *655*, 159629.

[43] P. Yu, F. Luo, S. Chen; Porous MoP_2_/MoS_2_ Hierarchical Nanowires for Efficient Hydrogen Evolution Reaction in Full pH Range. *J. Alloys Compd.* **2024**, *985*, 174024.

[44] J. Ge, D. Zhang, J. Jin, X. Han, Y. Wang, F. Zhang, X. Lei; Oxygen Atoms Substituting Sulfur Atoms of MoS_2_ to Activate the Basal Plane and Induce the Phase Transition for Boosting Hydrogen Evolution. *Mater. Today Energy* **2021**, *22*, 100854.

[45] X. Yang, Z. Wu, Z. Xing, C. Yang, W. Wang, R. Yan, C. Cheng, T. Ma, Z. Zeng, S. Li, C. Zhao; IrPd Nanoalloy-Structured Bifunctional Electrocatalyst for Efficient and pH-Universal Water Splitting. *Small* **2023**, *19*, 2208261.

[46] B. Wang, Y. Liu, J. Hao, J. Zhong, F. Yu, K. Zhang, H. Shen, B. Mao; Synergetic Optimization via Composition-Dependent Nanostructuring in Co-Mo-S Electrocatalysts for Efficient Hydrogen Evolution in Alkaline Solution. *Int. J. Electrochem. Sci.* **2018**, *13*, 3501-3515.

[47] Y. Zhang, X. Wang, X. Song, H. Jiang, Collaborative Effect Between Single-Atom Re and S Vacancy on Modulating Localized Electronic Structure of MoS_2_ Catalysts for Alkaline Hydrogen Evolution. *Nano Res.* **2024**, *17*, 9507-9517.

[48] X. Huang, H. Xu, D. Cao, D. Cheng; Interface Construction of P-Substituted MoS_2_ as Efficient and Robust Electrocatalyst for Alkaline Hydrogen Evolution Reaction. *Nano Energy* **2020**, *78*, 105253.

[49] G. Wang, G. Zhang, X. Ke, X. Chen, X. Chen, Y. Wang, G. Huang, J. Dong, S. Chu, M. Sui; Direct Synthesis of Stable 1T-MoS_2_ Doped with Ni Single Atoms for Water Splitting in Alkaline Media. *Small* **2022**, *18* 2107238.
